# CD5^+^ dendritic cell robots mediated in situ immunocyte activation

**DOI:** 10.1126/sciadv.aee5305

**Published:** 2026-08-12

**Authors:** Chuanhua Li, Weiwei Zhang, Xuyang Chen, Shixiao Ding, Lin Wang, Qian Wang, Songlin Yu, Qiran Zhu, Jiawen Niu, Ying Cui, Mengmeng Sun, Jie Zhao, Tianlong Li

**Affiliations:** ^1^ State Key Laboratory of Robotics and System, Harbin Institute of Technology, Harbin 150001, China.; ^2^ Suzhou Research Institute of HIT, Suzhou 215104, China.; ^3^ School of Mechanical and Power Engineering, Zhengzhou University, Zhengzhou 450001, China.; ^4^ Department of Radiation Oncology, Harbin Medical University Cancer Hospital, Harbin 150081, China.; ^5^ Department of Orthopedic Surgery, The Second Affiliated Hospital of Harbin Medical University, Harbin 150086, China.; ^6^ Department of Biomedical Engineering, National University of Singapore, Singapore 117583, Singapore.

## Abstract

Immune cell microbots with self-propelling and navigating capabilities have become an exciting field of research. Isolation, activation, and adoptive transfer of cytotoxic immune cells in vitro are some of the most common technical methods. However, the ex situ activation and adoptive transfer of cytotoxic immune cells carry the risk of systemic inflammatory responses and functional exhaustion. To address this bottleneck, we report a CD5^+^ dendritic cell microbot (CD5^+^DC robot) constructed by engineering natural CD5^+^ dendritic cells to phagocytose magnetic nanoparticles coated with tumor cell membranes. The PD-L1 on the surface of CD5^+^ cells is preblocked to enhance immune activation capacity. Under switched exogenous rotating/conical magnetic fields, CD5^+^DC robots form chain-like clusters for upstream motion or ribbon-like clusters for downstream motion within the vasculature, enabling precise aggregation at intestinal targets. Then, they navigate along tumor chemokine gradients, penetrating the extracellular matrix barrier via positive chemotaxis to infiltrate deep into tumor tissues and trigger cascading activation of resident immune cells. Distinct from traditional cellular microbot therapies that rely on self-mediated cytotoxicity, this CD5^+^DC robot system activates durable antitumor immunity by reprogramming innate immune cells within the tumor microenvironment. This strategy offers a novel pathway toward precision-targeted therapy with excellent biocompatibility and functional stability.

## INTRODUCTION

In the realm of clinical application, swimming microrobots with autonomous propulsion and targeted navigation capabilities are emerging as transformative tools in precision medicine (*1*–*10*). Inspired by microbial motility, these devices, particularly magnetically actuated variants, leverage external magnetic fields to navigate complex bodily fluids (*11*–*20*). This enables access to anatomically inaccessible tissues for applications ranging from targeted drug delivery to immune modulation (*21*, *22*). While initial research focused on single-robot control, recent advancements have enabled coordinated swarm dynamics (*23*–*25*), facilitating tasks such as gastrointestinal retention (*26*–*30*), ocular/brain drug targeting (*31*–*34*), and cellular membrane manipulation (*35*–*38*).

Despite substantial advancements, the current system still exhibits critical limitations. Most inorganic or polymeric microrobots are constrained in terms of immunocompatibility, deep tissue penetration, and active remodeling of the immune tumor microenvironment (TME) (*39*–*41*), hindering their ability to generate sufficient, sustained, and controllable effects within the tumor core. As an emerging approach, cell-hybrid microrobots combine the innate chemotactic capabilities, immune camouflage, and biocompatibility of living cells with the controllable actuation and payload capacities of engineered carriers (*10*, *42*–*44*). Dual-responsive neutrophil microrobots, for example, have been applied in postoperative glioma therapy and exhibited excellent drug targeted delivery efficiency (*45*). However, some reported cell-based platforms are prone to functional exhaustion and insufficient in situ activation within the strongly immunosuppressive TME or carry risks of systemic inflammation during adoptive transfer (*46*–*49*). Thus, achieving both efficient in situ immune activation in strongly immunosuppressive environments and circumvention of inflammatory risks during systemic navigation of cell-hybrid microrobots remains a key scientific and engineering challenge for clinical translation.

Dendritic cells (DCs) are recognized as one of the most potent antigen-presenting cells, playing a crucial role in initiating immune responses and activating immune cells (*50*, *51*). A recent study has found that merely reprogramming tumor cells into DCs is sufficient to remodel the tumor immunosuppressive microenvironment, thereby inducing tumor regression (*52*). Taking advantage of these properties, DCs have been developed as a therapeutic target for tumor therapy (*53*–*55*). Notably, CD5 expression on DCs correlates directly with effector T cell priming and patient prognosis and markedly enhances response to immunosuppressants (*56*). Compared with conventional CD5^−^DCs, CD5^+^DCs represent a specialized subset with extraordinary immunostimulatory potency. They inherently exhibit enhanced secretion of pro-inflammatory cytokines and superior efficiency in effector T cell priming, enabling effective penetration of the immunosuppressive barrier in the TME and fundamentally overcoming the functional exhaustion of conventional DCs at tumor sites. This unique attribute renders them highly promising as ideal carriers for microrobotic applications. In contrast to conventional DC-based or other cell-hybrid microrobots, CD5^+^DC microrobots enable sustained and robust in situ immune activation deep within solid tumors. Moreover, by leveraging the specificity of antigen presentation, these microrobots strictly confine inflammatory responses to tumor lesions, circumventing the systemic inflammation risks associated with adoptive transfer, and thus provide a transformative technological route for precision cancer immunotherapy. In light of this, we propose the following concept: Through the robotic engineering of CD5^+^DCs, efficient activation of in situ immune cells can be achieved. This strategy not only enables targeted counteraction against the tumor immunosuppressive microenvironment but also effectively circumvents the risks of systemic inflammation associated with adoptive transfer.

**Fig. 1.**
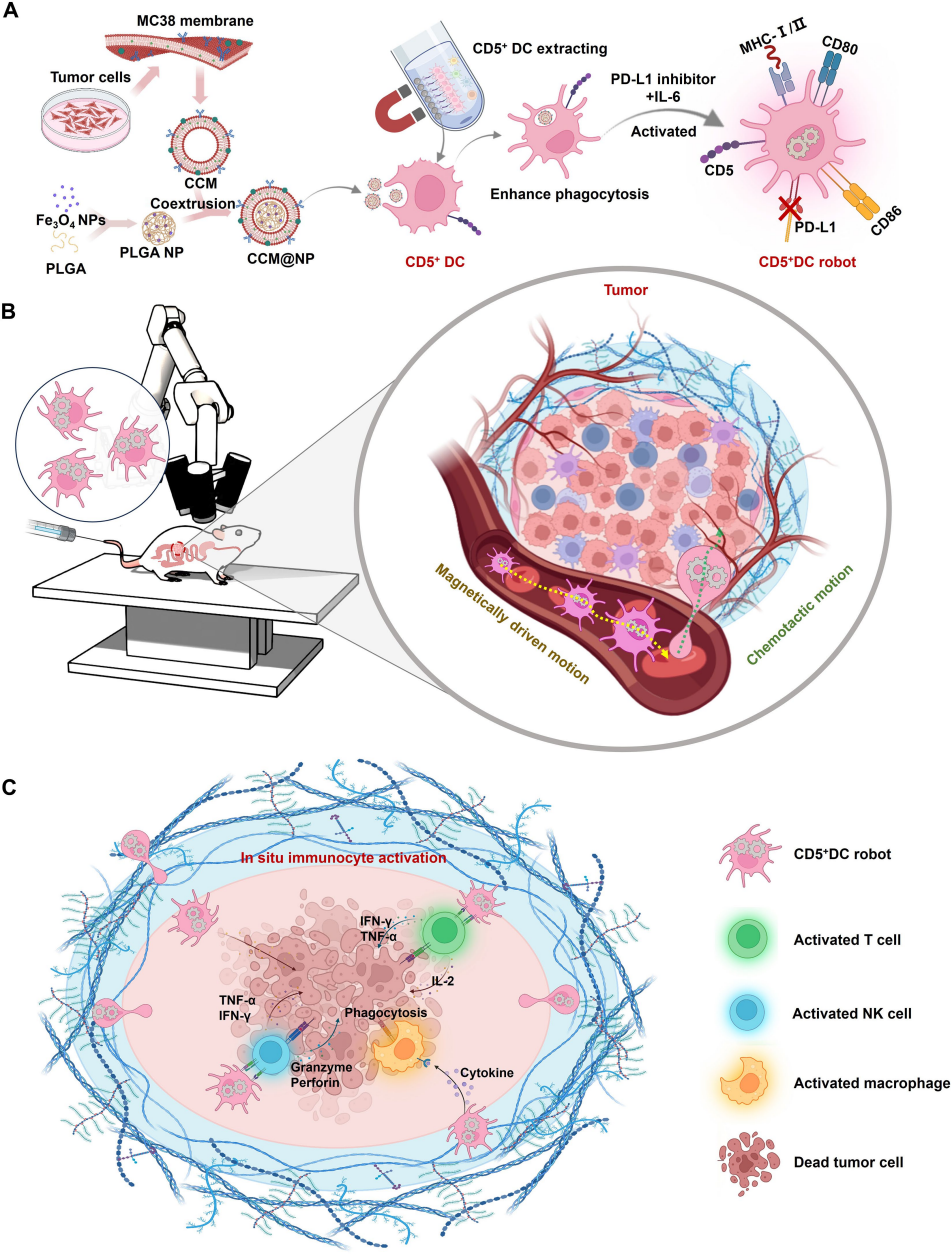
Schematic of CD5^+^DC robots mediating in situ immunocyte activation for tumor killing in vivo. (**A**) Schematic of the three-step fabrication of the CD5^+^DC robots. First, PLGA NPs are synthesized via an emulsion/solvent evaporation method. Second, CCM@NPs are prepared using a coextrusion technique. Third, CD5^+^DC robots are constructed, where primary CD5^+^DCs (sorted by CD5^+^ antibody–conjugated magnetic beads) internalize CCM@NPs through phagocytosis. (**B**) Schematic of the active targeted delivery of CD5^+^DC robots to malignant colorectal tumors: The robots first actively accumulate at tumor sites under an external magnetic field, followed by chemotactic migration along chemokine concentration gradients. (**C**) CD5^+^DC robot–mediated in situ activation of cytotoxic immune cells for in vivo tumor killing. Created in BioRender. C. Li (2026), https://biorender.com/nsxplha.

Here, we report an engineered CD5^+^DC microbot, called a CD5^+^DC robot, which can effectively migrate into the interior of tumor tissues and mediate in situ immune activation within the tumor. The CD5^+^DC robots express CD5 on their surface and have blocked surface programmed death-ligand 1 (PD-L1) (Fig. 1A): This design enhances CD5^+^DC robot function, relieves immunosuppressive signals, and directly boosts the CD5^+^DC robots’ capacity to activate tumor-killing immune cells in situ. Internally, the CD5^+^DC robots phagocytose poly(lactic-*co*-glycolic acid) magnetic nanoparticles (PLGA NPs) coated with cancer cell membranes (CCM@NPs). The cancer cell membrane (CCM) camouflage enhances magnetic cargo internalization through homologous adhesion molecules (e.g., integrins and cadherins) that mediate specific binding between CCM and CD5^+^DCs, which boosts the robots’ magnetic responsiveness. More critically, the CCM camouflage retains a full repertoire of tumor-associated antigens. After phagocytosis, these tumor-associated antigens are degraded in endolysosomes, processed into peptide fragments, and loaded onto major histocompatibility complex class I/II (MHC-I/II) molecules. This cascade concurrently up-regulates costimulatory molecules (e.g., CD86) on CD5^+^DCs, driving their maturation and robust activation of antigen-specific effector immune cells such as CD8^+^ cytotoxic T cells. Under conical magnetic fields, CD5^+^DC robots assemble into ribbon-like clusters. This form allows them to move efficiently downstream motion within the vasculature. Under rotating magnetic fields (RMFs), CD5^+^DC robots assemble into chain-like clusters. The chain structure reduces fluid resistance. Then, they adhere to the vascular wall to achieve upstream motion against blood flow. Under exogenous magnetic fields (conical/rotating), CD5^+^DC robots efficiently accumulate at tumor sites. Then, they penetrate deep into tumors via chemotaxis, enabling sustained, robust in situ immune activation (Fig. 1, B and C). By shifting the therapeutic paradigm from exogenous cell delivery to endogenous in situ immune activation, this strategy resolves the functional exhaustion of adoptively transferred killer immune cell robots when encountering the tumor immunosuppressive microenvironment. In addition, the CD5^+^DC robots’ swarm locomotion capability and antigen presentation–mediated highly specific cytotoxicity confine inflammation to the tumor site, greatly mitigating systemic toxicity risks. The CD5^+^DC robots developed in this study exhibited the capability to harness the natural capabilities of CD5^+^DCs (figs. S1 to S6) that would otherwise be difficult to replicate. Such CD5^+^DC robots may advance the realization of sustained in situ immune activation and durable precision cancer immunotherapies.

## RESULTS

### Preparation and characterization of CD5^+^DC robots

**Fig. 2.**
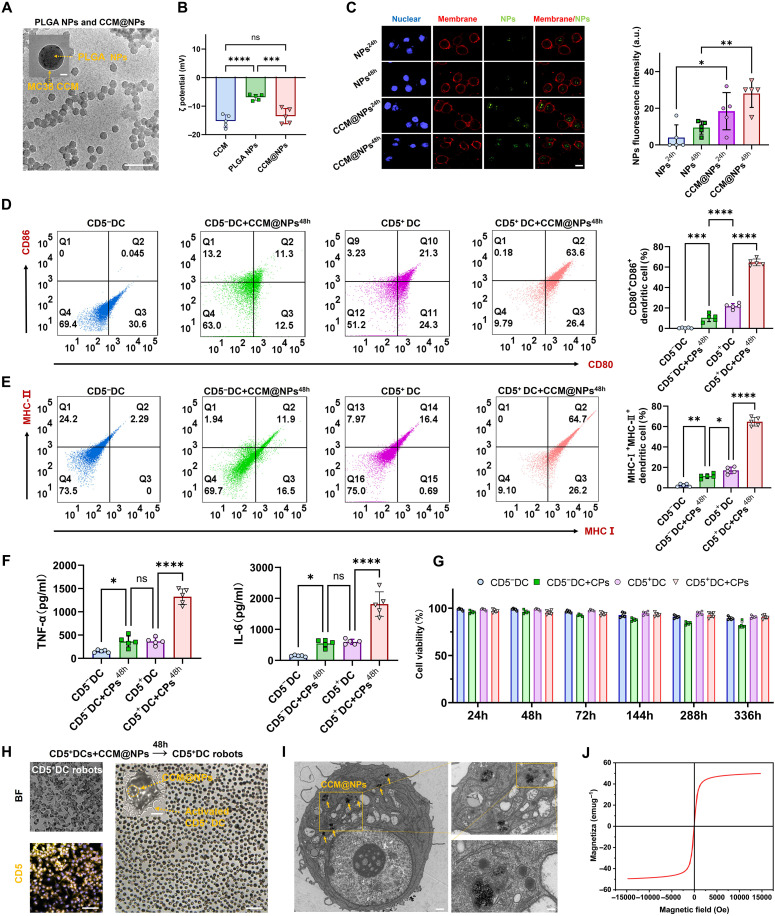
Construction of CD5^+^DC robots. (**A**) TEM images of PLGA NPs and CCM@NPs. Scale bars, 500 nm. (**B**) ζ potential of CCM, PLGA NPs, and CCM@NPs. (**C**) CLSM images of CD5^+^DCs phagocytosing NPs or CCM@NPs. Scale bar, 10 μm. a.u., arbitrary units. (**D** and **E**) Flow cytometry plots of CD5^−^DC, CD5^−^DC + CCM@NPs^48h^, CD5^+^DC, and CD5^+^DC + CCM@NPs^48h^ groups showing high expression of MHC-I, MHC-II, and costimulatory molecules CD80 and CD86 before and after coculture with CCM@NPs. (**F**) ELISA quantitative analysis of TNF-α and IL-6 cytokine levels in CD5^−^DC, CD5^−^DC + CCM@NPs^48h^, CD5^+^DC, and CD5^+^DC + CCM@NPs^48h^ groups. (**G**) Statistical results of live/dead staining for CD5^−^DCs and CD5^+^DCs before and after co-incubation with CCM@NPs for 24, 48, 72, 144, 288, and 336 hours (h). (**H**) Characterization of CD5 expression in adherent CD5^+^DC robots (left) and their bright-field (BF) morphology after digestion (right). Scale bar (top), 5 μm. Scale bar (bottom), 90 μm. (**I**) TEM image of CD5^+^DC robots. Scale bar (left), 2 μm. Scale bar (right), 150 nm. Yellow arrows: CCM@NPs. (**J**) Magnetization (M) loop from SQUID analysis of CD5^+^DC robots. *P < 0.05; **P < 0.01; ***P < 0.001; ****P < 0.0001.

The fabrication of CD5^+^DC robots involves three key steps: (i) synthesis of PLGA NPs, (ii) coating of these PLGA NPs with CCM, and (iii) phagocytosis by CD5^+^DCs sorted with CD5^+^ antibody–conjugated magnetic beads (Fig. 1A). PLGA NPs were prepared via an emulsion/solvent evaporation method, incorporating magnetic nanoparticles to enable external magnetic control. Subsequently, membranes derived from MC38 cancer cells were reconstituted into vesicles and coated onto the nanoparticles through coextrusion using 200-nm polycarbonate membranes. Transmission electron microscopy (TEM) showed that the resulting CCM@NPs exhibited a spherical core-shell structure with an average diameter of 156 nm (Fig. 2A). Zeta potential measurements in Fig. 2B confirmed successful membrane coating, with surface charges shifting from −6.88 ± 1.047 mV (naked PLGA nanoparticles) to −13.51 ± 2.652 mV (CCM@NPs), closely matching the potential of pure CCM vesicles (−15.202 ± 2.132 mV).

The CCM coating strategy effectively promoted the cellular uptake of magnetic nanoparticles by CD5^+^ dendritic cells (CD5^+^DCs). Confocal laser scanning microscopy (CLSM) following the co-incubation of green-fluorescently labeled bare PLGA NPs and CCM-coated PLGA NPs (CCM@NPs) revealed that CCM@NPs were rapidly internalized within 24 hours. By 48 hours, the intracellular fluorescence intensity was further augmented (Fig. 2C). Quantitative analysis of the green fluorescence within CD5^+^DCs demonstrated that the intracellular fluorescence levels in the CCM@NP group were significantly higher than those in the bare PLGA NP group, verifying that the CCM coating facilitated the phagocytosis of the nanoparticles by CD5^+^DCs.

Subsequently, we evaluated the activation and immunomodulatory effects of 48-hour CCM@NP co-incubation on CD5^−^ and CD5^+^DC subsets using flow cytometry and enzyme-linked immunosorbent assay (ELISA). Flow cytometric analysis demonstrated that postincubation, the proportion of the CD80^+^CD86^+^ double-positive subset in CD5^−^DCs increased from 0.045 to 11.3%, while the MHC-I^+^MHC-II^+^ double-positive subset increased from 2.29 to 11.9%. In stark contrast, CD5^+^DCs exhibited a much more robust activation response: Upon co-incubation, the CD80^+^CD86^+^ double-positive subset surged from 21.3 to 63.6%, and the MHC-I^+^MHC-II^+^ subset increased from 16.4 to 64.7% (Fig. 2, D and E). Mechanistically, the up-regulation of MHC-I/II—the primary signal for antigen presentation—directly confirms that the tumor antigens delivered by CCM@NPs were efficiently taken up, processed, and presented by DCs, establishing the core molecular foundation for the in situ immune activation function of CD5^+^DCs. Furthermore, the high expression of CD80/CD86, acting as the costimulatory secondary signal for T cell activation, endows DCs with a potent capability to stimulate immune cells. These results explicitly confirm that CD5^+^DCs inherently have a substantially higher baseline activation level than CD5^−^DCs, and following CCM@NP stimulation, the extent of their activation (double-positive subset proportion) vastly outperforms that of CD5^−^DCs. This disparity stems from the intrinsic immune activation potential of CD5^+^DCs, fully highlighting their natural advantage as immune activation vectors and the efficacy of CCM@NPs in promoting CD5^+^DC activation.

To further validate the activation status of the DCs, we measured the secretion levels of core pro-inflammatory cytokines, tumor necrosis factor–α (TNF-α) and interleukin-6 (IL-6), in the culture supernatants. Results indicated that upon CCM@NP stimulation, the secretion of TNF-α and IL-6 by CD5^−^DCs exhibited only a mild increase (both *P* < 0.05). Notably, the baseline pro-inflammatory cytokine secretion of CD5^+^DCs was already comparable to that of activated CD5^−^DCs [no statistical difference (ns)]. However, following stimulation, TNF-α and IL-6 secretion by CD5^+^DCs spiked highly significantly (both *P* < 0.0001), reaching levels 3.7-fold and 3.3-fold higher than those of the stimulated CD5^−^DC group, respectively (Fig. 2F). From an immunoregulatory perspective, TNF-α and IL-6 serve as the tertiary signal for immunological amplification in DC-mediated immune cell activation, synergistically potentiating antitumor immunity. In particular, TNF-α can directly induce tumor cell apoptosis while simultaneously activating innate immune cells, such as natural killer (NK) cells and macrophages, thereby rapidly initiating local antitumor immune responses. Meanwhile, IL-6 promotes the proliferation and differentiation of CD4^+^ helper T cells and CD8^+^ cytotoxic T cells, enhances B cell antibody secretion, and recruits immune cell infiltration into the TME, providing sustained momentum for in situ immune activation.

Ultimately, we fabricated CD5^+^DC robots in bulk by co-incubating CCM@NPs with CD5^+^DCs for 48 hours (fig. S7). CCM@NPs exhibited excellent biocompatibility with CD5^+^DCs. Quantitative live/dead staining data revealed that even after a 336-hour co-incubation, the viability of CD5^+^DCs remained above 90% (Fig. 2G), a critical characteristic enabling the CD5^+^DC robots to exert long-acting immune activation. The ubiquitous surface expression of CD5 was confirmed via CLSM and flow cytometry (Fig. 2H, left). Following trypsinization, typical dendritic synapse structures characteristic of activated DCs were clearly observed under bright-field microscopy (Fig. 2H, right) and scanning electron microscopy (fig. S8). High-resolution TEM of the bulk-prepared CD5^+^DC robots revealed intracellular phagosomes containing CCM@NPs, alongside scattered magnetic nanoparticles within the cytosol—attributed to the lysosomal degradation and subsequent release of PLGA NPs (Fig. 2I). Quantitative loading assays (fig. S9) indicated a significantly higher magnetic nanoparticle loading capacity in the CD5^+^DC group compared to the CD5^−^DC group, driven by the higher activation state and consequently stronger phagocytic capability of CD5^+^DCs. The internalized magnetic nanoparticles endowed the CD5^+^DC robots with high saturation magnetization. Superconducting quantum interference device (SQUID) magnetometry at 298 K demonstrated that the fabricated CD5^+^DC robots exhibited typical superparamagnetic behavior with a saturation magnetization (*M*_s_) of 49.8 emu/g (Fig. 2J and fig. S6), thereby enabling precise magnetic navigation under external magnetic field actuation.

### Magnetic motion and chemotaxis of CD5^+^DC robots

DC robots require dual responsiveness to achieve precise targeting of colorectal tumors. The first stage involves magnetic responsiveness, enabling highly efficient and controllable directional migration that facilitates robust accumulation of CD5^+^DC robots near tumor sites. This magnetic guidance not only enhances enrichment efficiency but also reduces off-target distribution to healthy tissues and organs. The second stage triggers chemotactic amoeboid motility in response to tumor-released chemokines. Although chemotaxis-driven movement is slower than magnetically controlled locomotion, the innate amoeboid properties of DCs allow CD5^+^DC robots to penetrate the extracellular matrix (ECM) barrier of solid tumors, enabling activation of intratumoral immune cell populations.

**Fig. 3.**
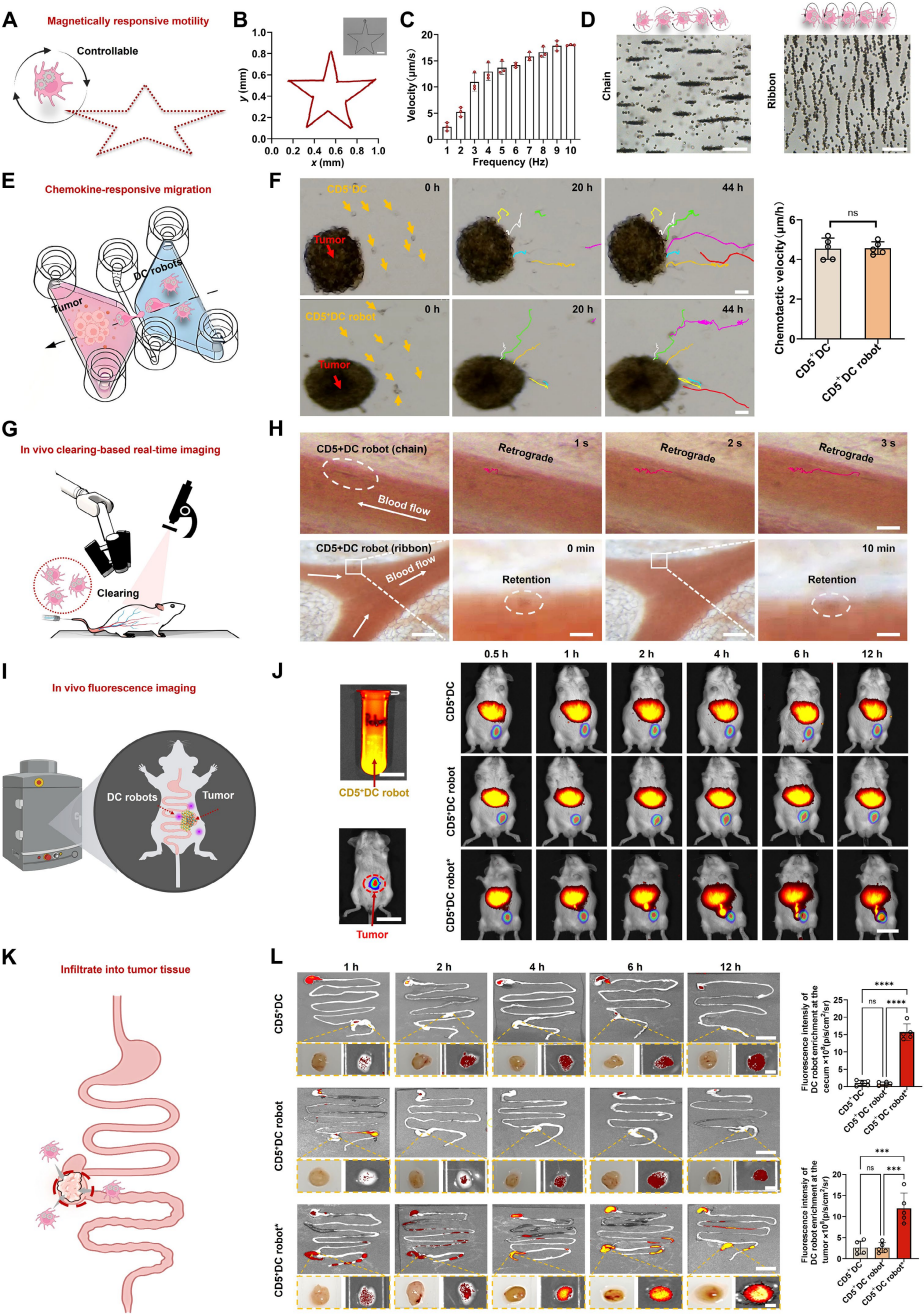
Magnetic motion and chemotaxis of CD5^+^DC robots. (**A**) Schematic of CD5^+^DC robot’s motion along a programmed path. (**B**) Time-lapse image and trajectory of the controllable magnetic propulsion of CD5^+^DC robot in a predefined star-shaped trajectory. Scale bar, 30 μm. (**C**) Variable speed of CD5^+^DC robots while controlling the frequency of movement. (**D**) Microscopic bright-field photographs of CD5^+^DC robots that exhibit “Chain” and “Ribbon” clusters, respectively, under the RMF and the conical magnetic field. Scale bars, 100 μm. (**E**) Schematic of the chemotactic motion of CD5^+^DC robots along a gradient of chemokine. (**F**) Time-lapse images of the chemotactic movement of CD5^+^DCs and CD5^+^DC robots along a chemokine gradient from tumor cells. Scale bars, 20 μm. (**G**) Schematic of magnetic actuation and real-time imaging of CD5^+^DC robots in abdominal intestinal blood vessels of tartrazine-cleared in vivo mice via a stereomicroscope. (**H**) In vivo visualization of CD5^+^DC robots forming chain-like clusters under an RMF (15 mT, 10 Hz) to achieve real-time retrograde blood flow movement and stable parking in living mice. Scale bar, 200 μm. (**I**) Schematic illustration of in vivo fluorescence/bioluminescence imaging of CD5^+^DC robots. (**J**) Active accumulation of CD5^+^DC robots (labeled with DiR) to colorectal cancer (where the luciferase gene, abbreviated as *luc*, is used for bioluminescence) through magnetic propulsion and chemotaxis. Scale bar, 2 cm. (**K**) Schematic illustration of CD5^+^DC robots infiltrating into tumor tissues via magnetic guidance and chemotaxis. (**L**) In vivo fluorescence imaging was used to evaluate robot infiltration into cecal and tumor tissues in the CD5^+^DC, CD5^+^DC robot, and CD5^+^DC robot* groups. Scale bars (top), 2 cm. Scale bars (bottom), 2 mm. ***P < 0.001; ****P < 0.0001. Schematics created in BioRender. J. Niu (2026), https://biorender.com/8e1uq5b.

To enable tumor-directed navigation, we developed a vision-based magnetic navigation system comprising an optical microscope for real-time monitoring, a uniform magnetic field generator, and a closed-loop feedback module for motion planning (fig. S10). By integrating real-time position tracking, autonomous path optimization, and visual feedback control, we achieved programmable trajectory following in CD5^+^DC robots. Figure 3A illustrates the motion schema of magnetically driven CD5^+^DC robots. Time-lapse trajectories in Fig. 3B and movies S1 and S2 demonstrate controlled movement along a pre-set pentagram-shaped path, including right-angle turns in response to frequency-modulated magnetic fields (fig. S11). Under a 10-mT RMF, CD5^+^DC robots exhibited frequency-dependent rolling velocities ranging from 2.4 to 17.9 μm/s as the field frequency increased from 1 to 10 Hz (Fig. 3C). Collective control of CD5^+^DC robots is critical for biomedical applications. Magnetic rotation on substrates induced local hydrodynamic flows, promoting assembly of adjacent CD5^+^DC robots via fluid-structure interactions and dipole-dipole couplings. As shown in Fig. 3D, dispersed CD5^+^DC robot populations (liquid state) self-organized into chain-like clusters to reduce fluid resistance and achieve upstream motion against blood flow or ribbon-like clusters to enable efficient downstream motion (movies S3 to S7). Under RMFs and conical magnetic fields, the lengths of chain-like clusters and ribbon-like clusters exhibit differences. When the number of robots in the cluster (Ns) is 6, the chain-like clusters reach the maximum movement speed (fig. S12); when Ns is 12, the ribbon-like clusters achieve the maximum movement speed (fig. S13). Large-scale chain-like and ribbon-like clusters both show magnetic field frequency dependence, with their maximum speeds attained at 50 Hz (figs. S14 and S15). At TMEs, chemokine gradients (generated by MC38 tumor cell–seeded left chambers in microfluidic channels; Fig. 3E) induced directional migration of both CD5^+^DC robots and naive CD5^+^DCs. Time-lapse imaging showed that both cell types preferentially accumulated toward chemokine-rich hydrogel regions containing MC38 tumor cells (Fig. 3F and movies S8 and S9). Quantitative analysis further confirmed no significant difference in chemotactic velocity or chemotactic responsiveness between CD5^+^DC robots and naive CD5^+^DCs, demonstrating that robot fabrication did not compromise the intrinsic chemotactic ability of CD5^+^DCs.

In vivo experiments were conducted to evaluate CD5^+^DC robots’ dual-responsive targeting capability in complex biological environments and their potential for intratumoral immune activation. Orthotopic colorectal cancer models were established by cecal injection of luciferase-expressing MC38 tumor cells. Tumor-bearing mice were randomized into the CD5^+^DC group, CD5^+^DC robot group, and magnetically driven CD5^+^DC robot (CD5^+^DC robot*) group. In vivo magnetic control coils are shown in fig. S16. First, we used tartrazine to perform abdominal clearing on mice, which enhanced the tissue penetration depth of white light and fluorescence. This enabled direct real-time imaging of the abdominal intestinal blood vessels in live mice via a stereomicroscope (Fig. 3G, fig. S17, and movie S10). As shown in fig. S18 and movie S11, under a conical magnetic field, CD5^+^DC robots injected via the tail vein form ribbon-like swarms with controllable motion in the intestinal blood vessels of in vivo mice. By varying the direction of the conical magnetic field, CD5^+^DC robots can be controlled to assemble into ribbon-like clusters either parallel or perpendicular to the vascular wall. DC robots form a ribbon-like structure parallel to the vascular wall and migrate toward and adhere to the vessel wall, thereby realizing controllable retention (Fig. 3H). Under an RMF, CD5^+^DC robots can self-assemble into chain-like swarms, thereby reducing their contact area with high-velocity blood flow and significantly decreasing fluid resistance. Meanwhile, on the basis of the hemodynamic characteristic that the flow velocity near the vascular wall is substantially lower than that in the vascular center, we adopted a strategy of having the chain-like swarms adhere to the vascular wall to achieve their retrograde movement (Fig. 3H, fig. S19, and movies S12 and S13). CD5^+^DC robots assembled into a chain-like swarm can also traverse microvessels—conferring critical advantages such as preserved hydrodynamic mobility to prevent vascular occlusion, enhanced tissue penetration capability, and superior adaptability to the tortuous microvascular network, thereby enabling efficient delivery to distant tumor lesions (fig. S20 and movie S14).

Furthermore, abdominal clearing enhances positional accuracy and real-time imaging speed for in vivo small-animal imaging. CD5^+^DC robots were labeled with DiR (1,1′-dioctadecyl-3,3,3′,3′-tetramethylindotricarbocyanine iodide) for in vivo bioluminescence imaging [in vivo imaging system (IVIS)]. A hybrid actuation strategy integrating conical magnetic fields and RMFs was used. Potassium fluorescein was initially administered to enable real-time tracing and monitoring of the tumor location. Thirty minutes after tail vein injection of CD5^+^DC robots, a conical magnetic field (15 mT, 15 Hz) was applied to induce the robots to assemble into band-like clusters parallel to the vascular wall, which subsequently migrated toward and adhered to the vessel wall. Spatial positions of the CD5^+^DC robots and the tumor were simultaneously visualized via in vivo fluorescence imaging, and the magnetic field direction was calibrated toward the tumor region according to the connecting line between the centroids of their respective fluorescence signals. Thereafter, an RMF (20 mT, 10 Hz) was used to drive the robots to form chain-like clusters for tumor-targeted locomotion. During 0.5 to 12 hours postadministration, in vivo fluorescence imaging was performed every 30 s, and the magnetic field direction was continuously calibrated on the basis of the directional vector from the fluorescence signal of CD5^+^DC robots to that of the tumor tissue. Fluorescence imaging revealed distinct biodistribution patterns (Fig. 3, I and J, and fig. S21). In the CD5^+^DC group and CD5^+^DC robot group, CD5^+^DC or CD5^+^DC robots showed persistent accumulation in the liver over 12 hours, likely attributed to hepatic first-pass metabolism and the sinusoidal vascular architecture. In the CD5^+^DC robot* group, initial hepatic accumulation at 1 hour was followed by progressive reorientation toward tumor sites between 2 and 6 hours postadministration. By 12 hours, CD5^+^DC robot enrichment coincided with tumor localization. Ex vivo fluorescence imaging of the gastrointestinal tract showed that in the CD5^+^DC group and CD5^+^DC robot group, CD5^+^DC or CD5^+^DC robots transiently accumulated in the stomach and rectum at 1 hour, followed by diffuse and low-level signals throughout the gastrointestinal tract from 1 to 12 hours. This pattern suggests unstable retention and systemic clearance. In contrast, in the CD5^+^DC robot* group, CD5^+^DC robots exhibited sustained enrichment in the stomach, colon, cecum, and rectum, with progressively increasing signals from 2 to 12 hours, demonstrating magnetic field–stabilized localization and time-dependent accumulation in target gastrointestinal regions. Quantitative analysis of excised tumors revealed a 4.6-fold increase in CD5^+^DC robot accumulation in the CD5^+^DC robot* group compared to the CD5^+^DC group and CD5^+^DC robot group (Fig. 3, K and L). These results confirm that the combination of external magnetic guidance and intrinsic chemotactic navigation significantly enhances intratumoral enrichment. Overall, this dual-responsive strategy enables spatiotemporal control of tumor targeting, integrating exogenous magnetic actuation with endogenous chemokine-driven migration to overcome physiological barriers in vivo.

### CD5^+^DC robots promote expansion and the activation of tumor-related immune cells in vitro

**Fig. 4.**
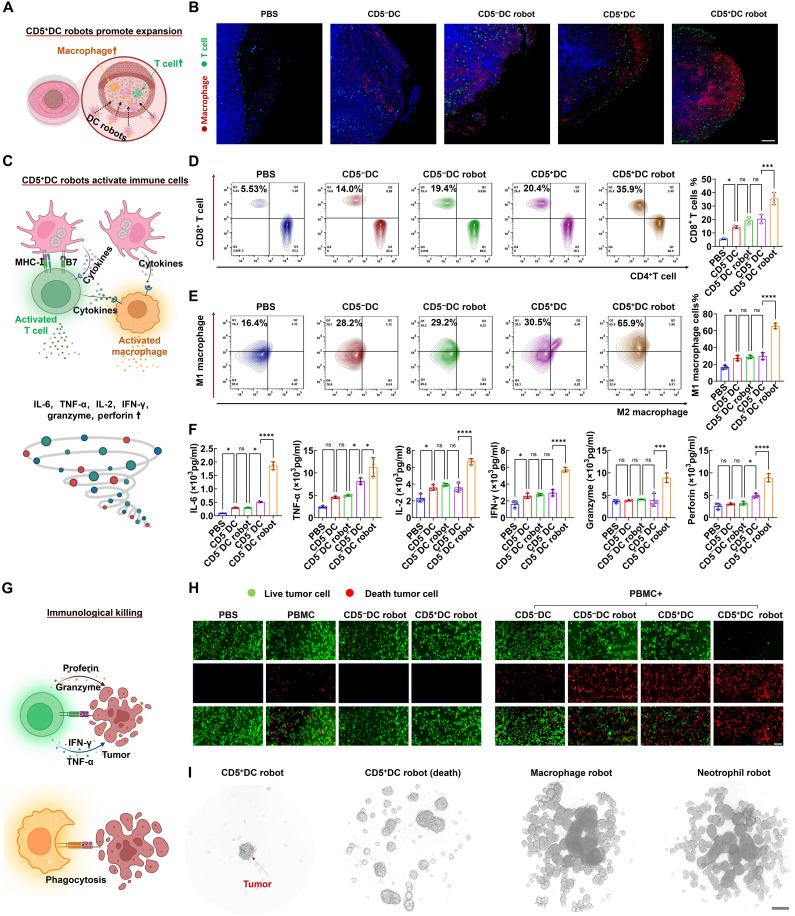
CD5^+^DC robots promote expansion and the activation of tumor–in situ immune cells in vitro. (**A**) Schematic illustration of CD5^+^DC robots infiltrating solid tumor tissues and the in situ expansion of T cells and macrophages. (**B**) Laser confocal micrographs of T cells and macrophages in thin tumor tissue sections in each of the following groups: the PBS group, the CD5^−^DC group, the CD5^−^DC robot group, the CD5^+^DC group, and the CD5^+^DC robot group. Scale bar, 100 μm. (**C**) Schematic diagram of the activation of T cells and macrophages by CD5^+^DC robots. (**D**) Flow cytometric analysis of T cells after co-incubation of tumor tissues with the above groups. (**E**) Flow cytometric analysis of macrophages under the above groups. (**F**) ELISA statistical results of cytokine levels under the above groups. (**G**) Schematic of activated T cells and macrophages mediating killing of MC38 tumor cells. (**H**) Fluorescence microscopy images of cell viability staining after 24-hour co-incubation of tumor cells with each of the following groups: PBS group, PBMC group, CD5^−^DC robot group, CD5^+^DC robot group, PBMC + CD5^−^DC group, PBMC + CD5^−^DC robot group, PBMC + CD5^+^DC group, or PBMC + CD5^+^DC robot group. Note that PI stains dead cells, and annexin V stains live cells. Scale bar, 150 μm. (**I**) Microscopic images of tumor organoid colony formation after co-incubation with the following groups: CD5^+^DC robot group, CD5^+^DC robot (death) group, macrophage robot group, and neutrophil robot group. Scale bar, 100 μm. *P < 0.05; ***P < 0.001; ****P < 0.0001. Schematics created in BioRender. C. Li (2026), https://biorender.com/v2xcb3u.

The TME releases cytokines that suppress the expression of MHC-II molecules and costimulatory molecules CD80/CD86 on DCs. This inhibition disrupts antigen presentation and blocks immune cell activation within the TME, creating an immunosuppressive niche conducive to tumor progression. Previously, we achieved ex vivo activation of CD5^+^DCs and loading of magnetic nanoparticles. CD5^+^DCs, as the most potent APCs, exhibit superior capabilities in antigen presentation and immune cell activation (fig. S22). Following their penetration through the ECM of solid tumors, these CD5^+^DC robots are poised to promote the proliferation and activation of intratumoral immune cells, including T cells and macrophages. To validate this process, we conducted a series of coculture experiments with CD5^+^DC robots in vitro. Solid tumor tissues excised from mice were cocultured with Cy5 (cyanine 5)–labeled CD5^+^DC robots for 24 hours, followed by thin sectioning. Confocal microscopy and quantitative fluorescence analysis revealed that CD5^+^DC robots migrated from the tumor periphery through the ECM into the tumor core, a critical prerequisite for intratumoral immune cell activation (Fig. 4A and fig. S22). Immunostaining tumor sections with T cell– and macrophage-specific fluorescent antibodies showed that CD5^+^DC robot coculture significantly increased the density of in situ T cells and macrophages (Fig. 4B), attributed to both activation-driven proliferation of resident T cells/monocytes and chemotactic recruitment by CD5^+^DC robots.

Tumor tissues were treated for 24 hours with phosphate-buffered saline (PBS), CD5^−^DCs, CD5^−^DC robots, CD5^+^DCs, or CD5^+^DC robots, followed by flow cytometry analysis (Fig. 4C). For T cell profiling (Fig. 4D and fig. S23), the CD5^+^DC robot group exhibited a 35.9% CD8^+^ cytotoxic T cell population—significantly higher than other groups (5.53, 14.0, 19.4, and 20.4% for PBS, CD5^−^DC, CD5^−^DC robot, and CD5^+^DC, respectively)—indicating that CD5^+^DC robots promote the recruitment, proliferation, and activation of cytotoxic effector CD8^+^ T cells, via surface-expressed activated MHC-I, CD80, and CD86. An increased proportion of CD8^+^ T cells facilitates the exertion of antitumor cytotoxic effects. Macrophage analysis (Fig. 4E and fig. S24) showed a 65.9% M1 (CD86^+^ pro-inflammatory) phenotype in the CD5^+^DC robot group, surpassing other groups (16.4, 28.2, 29.2, and 30.5%), demonstrating CD5^+^DC robot–mediated polarization from immunosuppressive M2 to antitumor M1 macrophages. ELISA quantification of intratumoral cytokines (Fig. 4F) revealed that CD5^+^DC robot–treated tissues had 2.46- to 8.87-fold higher levels of IL-2, IL-6, interferon-γ (IFN-γ), TNF-α, IL-1β, and perforin compared to PBS controls, reflecting robust immune activation. Peripheral blood mononuclear cells (PBMCs) were isolated from mouse peripheral blood. MC38 tumor cells were treated with PBS, PBMCs alone (PBMC), CD5^−^DC robot, CD5^+^DC robot, PBMC + CD5^−^DC, PBMC + CD5^−^DC robot, PBMC + CD5^+^DC, or PBMC + CD5^+^DC robot for 24 hours. Propidium iodide (PI)/annexin V staining demonstrated that PBS, CD5^−^DC robots, and CD5^+^DC robots alone exerted no significant direct cytotoxicity toward tumor cells, consistent with the biological feature of DCs as professional APCs lacking intrinsic tumoricidal activity. In coculture systems with PBMCs, the PBMC alone group and the PBMC + CD5^−^DC group showed only weak tumoricidal activity, which was derived from endogenous cytotoxic lymphocytes within PBMCs but remained limited because of insufficient activation. In contrast, the PBMC + CD5^−^DC robot group and the PBMC + CD5^+^DC group exhibited markedly enhanced tumor cell killing. This was attributed to the effective activation of CD5^−^DC robots by CCM@NPs, which promoted the activation of cytotoxic immune cells in PBMCs to eliminate tumor cells. Meanwhile, native CD5^+^DCs had considerable intrinsic immune-stimulatory capacity, yielding a comparable killing effect to the CD5^−^DC robot group (Fig. 4, G and H). Clonogenic assays involving MC38 tumor cells (with co-incubation of PBMC) cocultured with CD5^+^DC robots, CD5^+^DC robots (death), macrophage robots, or neutrophil robots revealed that CD5^+^DC robots retain robust in situ activation capacity, with diminished dependency on cellular life span (Fig. 4I). These findings lay a foundational framework for the achievement of high-efficacy and long-acting immunotherapeutic outcomes.

### CD5^+^DC robot–mediated in vivo immune activation

**Fig. 5.**
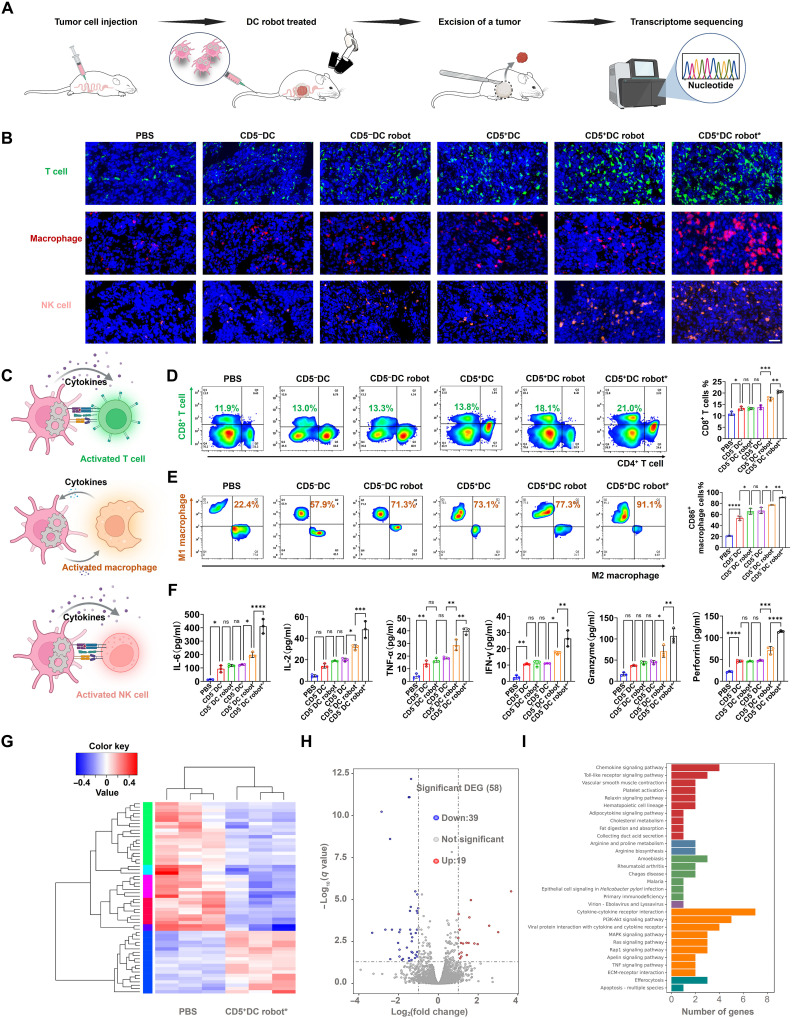
CD5^+^DC robot–mediated in vivo immune activation. (**A**) Schematic diagram of constructing a mouse tumor model and conducting flow cytometry and transcriptome sequencing of tumors after treatment. (**B**) CLSM images of immunofluorescence-stained tumor sections, showing the infiltration of T cells, macrophages, and NK cells into tumor tissues in mice treated via caudal vein injection with PBS, CD5^−^DCs, CD5^−^DC robots, CD5^+^DCs, CD5^+^DC robots without magnetic control, and CD5^+^DC robots with magnetic control. (**C**) Schematic diagram of the in vivo, in situ activation of T cells, macrophages, and NK cells in tumor tissues by CD5^+^DC robots. (**D**) Flow cytometry dot plots and statistical data of CD4^+^ T cells and CD8^+^ T cells induced in mice by caudal vein injection of PBS, CD5^−^DC, CD5^−^DC robots, CD5^+^DC, CD5^+^DC robots without magnetic control, and CD5^+^DC robots with magnetic control. (**E**) Flow cytometry dot plots and statistical data of M1 (CD86^+^) macrophages and M2 (CD206^+^) macrophages induced in mice by caudal vein injection of PBS, CD5^−^DC, CD5^−^DC robots, CD5^+^DC, CD5^+^DC robots without magnetic control, and CD5^+^DC robots with magnetic control. (**F**) Statistical result graph of the cytokines in tumor tissues detected by ELISA after mice were respectively injected via the caudal vein with PBS, CD5^−^DC, CD5^−^DC robots, CD5^+^DC, CD5^+^DC robots without magnetic control, and CD5^+^DC robots with magnetic control. (**G**) Heatmap of differential gene expressions in mouse tumor tissues treated with PBS and CD5^+^DC robots with magnetic control. (**H**) Volcano plots displayed the differentially expressed genes of CD5^+^DC robots with magnetic control compared to the PBS group. (**I**) Results of KEGG pathway analysis concerning the differentially expressed genes. *P < 0.05; **P < 0.01; ***P < 0.001; ****P < 0.0001. Schematics created in BioRender. C. Li (2026), https://biorender.com/326iwkk.

The TME in vivo represents an extremely complex physiological niche. To comprehensively validate the capacity of magnetically controlled CD5^+^DC robots to activate immunity and uncover potential tumor-killing molecular mechanisms, we performed flow cytometry, ELISA, and transcriptomic analysis on live tumor tissues from mice treated with PBS or CD5^+^DC robots (Fig. 5A). Mice were divided into six groups (tail vein injection): PBS, CD5^−^DCs, CD5^−^DC robots, CD5^+^DCs, non–magnetically controlled CD5^+^DC robots (CD5^+^DC robot), and magnetically controlled CD5^+^DC robots (CD5^+^DC robot*). Immunofluorescence staining of tumor tissues was performed to systematically evaluate the levels of tumor-infiltrating immune cells across different treatment groups: Green fluorescence–labeled T cells, red fluorescence–labeled macrophages, orange fluorescence–labeled NK cells, and blue fluorescence corresponded to DAPI (4′,6-diamidino-2-phenylindole)–stained cell nuclei (Fig. 5B). The results demonstrated that the PBS control group exhibited the weakest fluorescent signals and the lowest degree of immune cell infiltration in tumors. While the CD5^−^DC and CD5^−^DC robot groups showed a slight elevation in immune cell infiltration compared to the PBS group, their overall infiltration levels were significantly lower than those of the CD5^+^DC-related treatment groups. The CD5^+^DC and CD5^+^DC robot groups presented further increased infiltration of T cells, macrophages, and NK cells, confirming the superior intrinsic immune activation potential of CD5^+^DCs relative to CD5^−^DCs. Notably, the magnetically guided CD5^+^DC robot* group displayed markedly stronger fluorescent signals for all three types of tumor-infiltrating immune cells than all other experimental groups, fully verifying that the precise magnetic targeting delivery strategy substantially promotes the efficient enrichment of CD5^+^DC robots within tumor tissues, thereby potently activating in situ cytotoxic immune cells (T cells, macrophages, and NK cells) in the TME and eliciting the strongest local antitumor immune response. These data provide direct histological evidence supporting the excellent in vivo antitumor efficacy of CD5^+^DC robots. Figure 5C illustrates the process by which CD5^+^DC robots activate T cells, macrophages, and NK cells. Flow cytometry results (Fig. 5, D and E) showed that CD5^+^DC robots promoted the recruitment, activation, and proliferation of CD8^+^ cytotoxic T cells—the CD5^+^DC robot* group exhibited a 21.0% CD8^+^ T cell population, significantly higher than other groups (11.9, 13.0, 13.3, 13.8, and 18.1% for PBS, CD5^−^DC, CD5^−^DC robot, CD5^+^DC, and CD5^+^DC robot, respectively). This enables CD8^+^ cytotoxic T cells to exert direct tumor cytotoxicity. Macrophage polarization from immunosuppressive M2 to pro-inflammatory M1 phenotypes was also enhanced in the CD5^+^DC robot* group (91.1% versus 22.4, 57.9, 71.3, 73.1, and 77.3% in other groups). This allows M1 macrophages to support antitumor immunity via inflammation and phagocytosis. ELISA quantification of intratumoral cytokines revealed significantly elevated levels of IL-2, IL-6, IFN-γ, TNF-α, IL-1β, and perforin in the CD5^+^DC robot* group compared to other groups (Fig. 5F). This amplifies systemic antitumor responses and inhibits tumor proliferation.

Transcriptomic sequencing of treated tumor tissues identified distinct gene expression profiles (Fig. 5G). CD5^+^DC robot* treatment induced 19 gene up-regulations and 39 down-regulations in colorectal cancer tissues (Fig. 5H). Kyoto Encyclopedia of Genes and Genomes (KEGG) pathway enrichment analysis highlighted significant changes in immune-related pathways (Fig. 5I and fig. S25). The “cytokine-cytokine receptor interaction” pathway was prominently enriched, indicating enhanced cytokine-receptor interactions that facilitate activation of T cells and NK cells for improved tumor recognition. The “TNF signaling pathway” was also enriched, reflecting direct induction of tumor cell apoptosis and augmented immune cell cytotoxicity. Nonimmune pathways critical for tumor proliferation, including Ras, PI3K (phosphatidylinositol 3-kinase)-Akt, and MAPK (mitogen-activated protein kinase) signaling, were dysregulated in CD5^+^DC robot*–treated tissues, likely disrupting abnormal proliferative signals to inhibit tumor growth. The “apoptosis” pathway signature suggested regulation of proapoptotic gene expression, promoting programmed tumor cell death. Enrichment of inflammation-related pathways such as “rheumatoid arthritis” indicated that CD5^+^DC robot* treatment may recruit additional immune cells to the TME via controlled inflammatory responses, enhancing antitumor cytotoxicity.

Gene set enrichment analysis revealed that two key signal cascades related to metabolism and proliferation, specifically the mammalian target of rapamycin (mTOR) pathway and the MYC axis, were strongly negatively correlated with specific gene subsets in colorectal cancer tissues, as clearly demonstrated in fig. S26. This indicates that CD5^+^DC robot* treatment can effectively down-regulate these two signal cascades related to metabolism and proliferation. Once down-regulated, the mTOR pathway and the MYC axis jointly trigger a series of molecular responses, thereby inhibiting the progression of colorectal tumors. Down-regulation of the mTOR pathway, as a core regulatory pathway for cell growth, metabolism, and protein synthesis, effectively disrupts the growth-promoting metabolic program in tumor cells. This leads to a reduction in energy production and protein synthesis, thus impeding the rapid proliferation ability of tumor cells. Meanwhile, the MYC axis plays a crucial role in regulating cell-cycle progression, apoptosis, and gene expression. Its down-regulation further suppresses the proliferation ability of tumor cells. By regulating the expression of genes related to cell cycle–promoting factors and apoptosis-inhibiting factors, the down-regulation of the MYC axis induces growth arrest and apoptosis in colorectal tumor cells. Overall, the down-regulation of these two signal cascades by CD5^+^DC robot* treatment creates an unfavorable environment for colorectal tumor cells. This ultimately leads to the inhibition of tumor growth and promotes tumor cell death, offering a promising therapeutic strategy for colorectal cancer.

The differential gene interaction network diagram (fig. S27) presents the interaction relationships among numerous genes such as *Tnf*, *Ccl* family genes, etc. Each node represents a gene, and the connections between nodes represent interactions. For example, the *Tnf* gene can induce tumor cell apoptosis and enhance the activity of immune cells, and *Ccl* family genes can recruit immune cells to the tumor site. Their interactions can affect the recruitment and activation of immune cells and the survival of tumor cells. It is speculated that after CD5^+^DC robot* treatment, it may regulate the interaction of Tnf and related chemokine genes, enhance the expression and activity of *Tnf*, and promote the recruitment and activation of immune cells to the TME, enabling better recognition and killing of tumor cells.

### Evaluation of the anticancer therapeutic efficacy of CD5^+^DC robots in vivo

**Fig. 6.**
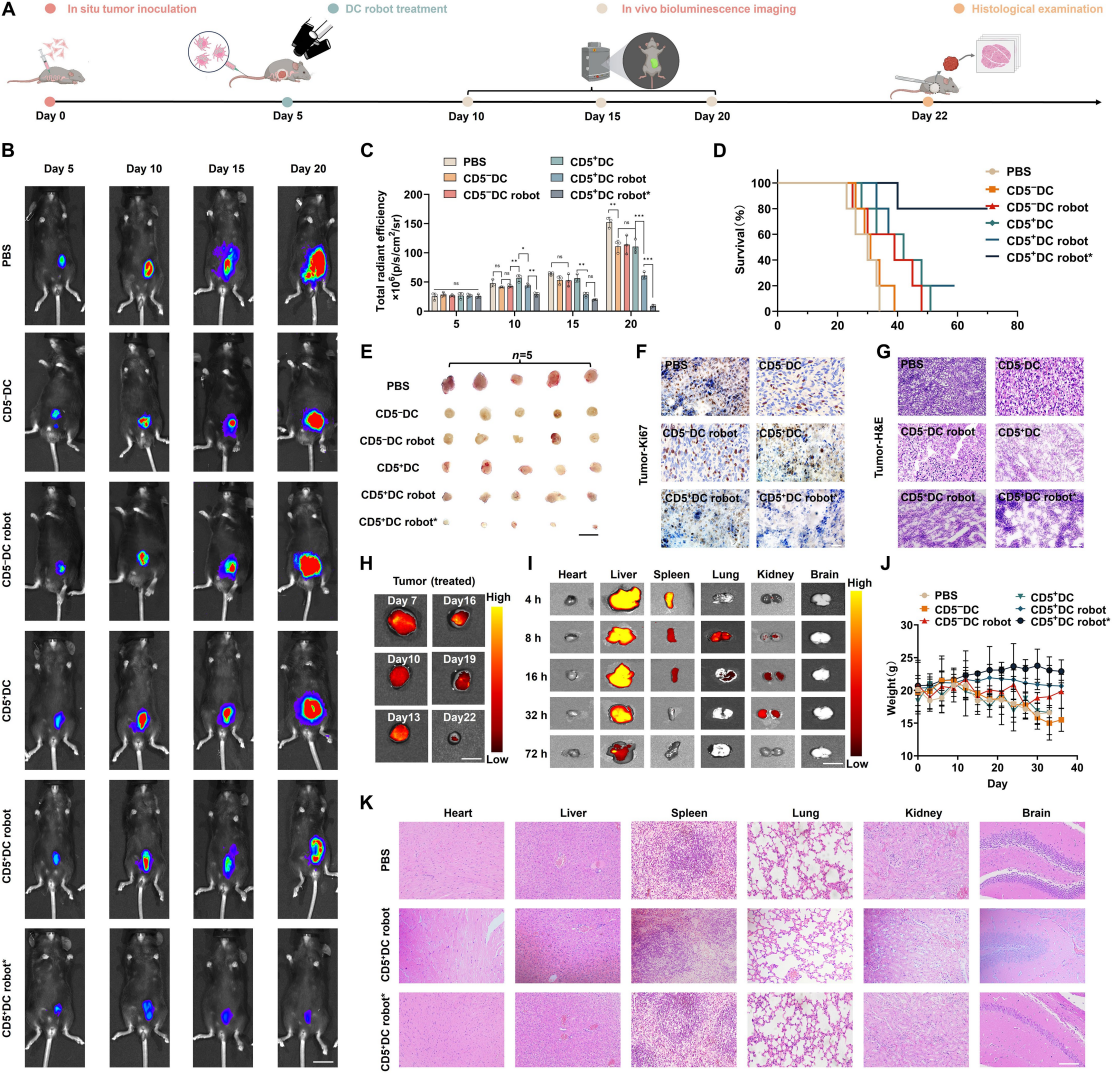
In vivo evaluation of the therapeutic efficacy and biosafety of CD5^+^DC robots using a colorectal tumor model. (**A**) Schematic of the study protocol in establishing orthotopic MC38 colorectal tumors in C57BL/6J mice, followed by treatment and imaging procedures. (**B** and **C**) Representative IVIS images of the luciferase-expressing MC38 colorectal tumor–bearing mice (B) and quantified relative bioluminescence intensity (C) upon various intravenous administrations over the treatment course, including PBS, CD5^−^DC, CD^−^DC robot, CD5^+^DC, CD5^+^DC robot, and CD5^+^DC robot*. Error bars in (C) represent the SD from five mice. Scale bar, 2 cm. (**D**) Survival curve of mice in different groups during the whole treatment process. (**E**) Images of colorectal tumor size at the end of the study for each group. Scale bar, 1 cm. (**F**) At the end of the study, Ki67 immunohistochemical staining was performed on the cross sections of colorectal tumors from MC38 tumor–bearing mice in different groups. Scale bar, 100 μm. (**G**) H&E staining of colorectal tumor cross sections from MC38 tumor–bearing mice of different groups at the end of the study. Scale bar, 150 μm. (**H**) In vivo fluorescence imaging of CD5^+^DC robot accumulation in tumor tissues. Scale bar, 5 mm. (**I**) In vivo fluorescence metabolic diagrams of CD5^+^DC robots in the heart, liver, spleen, lung, kidney, and brain of mice. Scale bar, 1 cm. (**J**) Body weights of mice in different groups during the whole treatment process. (**K**) H&E staining of histological sections of the mice in the PBS group, CD5^+^DC robot group, and CD5^+^DC robot* group. Heart, liver, spleen, lung, and kidney tissues were collected at the end of the study. Scale bar, 100 μm. Schematics created in BioRender. C. Li (2026), https://biorender.com/326iwkk.

An orthotopic murine colorectal cancer model was established by inoculating luciferase-expressing MC38 cells into mice, which took 5 days to develop. This model was designed to evaluate the in vivo therapeutic effects of CD5^+^DC robots. Tumor-bearing mice received a single tail vein infusion on day 5 post–model establishment, with treatment groups including PBS, CD5^−^DC, CD5^−^DC robot, CD5^+^DC, non–magnetically controlled CD5^+^DC robots (CD5^+^DC robot), and magnetically controlled CD5^+^DC robot (CD5^+^DC robot*). Tumor progression was monitored via bioluminescent signals from MC38 cells over a 20-day treatment period (Fig. 6A).

By day 15 after CD5^+^DC robot* treatment initiation, the CD5^+^DC robot* group exhibited a >90% reduction in bioluminescent signals, directly reflecting substantial tumor volume shrinkage. In contrast, groups treated with CD5^−^DC, CD5^−^DC robot, CD5^+^DC, or CD5^+^DC robot showed only moderate signal reductions compared to the PBS control (Fig. 6, B and C). End-point analysis revealed that colon tumors excised from the CD5^+^DC robot* treatment group were the smallest in volume (Fig. 6E), and survival curves demonstrated a significant prolongation of mouse life span in this group (Fig. 6D). Thin sections of excised tumors, stained with hematoxylin-eosin (H&E) and subjected to Ki67 immunohistochemistry, showed marked tumor damage and reduced proliferative capacity in the CD5^+^DC robot* group (Fig. 6, F and G), underscoring their superior antitumor efficacy. To investigate whether the duration of magnetic actuation affects the therapeutic efficacy, CD5^+^DC robots were alternately exposed to an in vitro conical magnetic field (15 mT, 15 Hz) and an RMF (20 mT, 10 Hz) for 0, 12, 24, 36, and 48 hours and then intravenously injected into tumor-bearing mice via the tail vein, followed by 12 hours of targeted magnetic actuation. On day 20, tumor fluorescence signals were detected by small animal in vivo fluorescence imaging. We found that the duration of magnetic actuation did affect the therapeutic efficacy, whereas no significant statistical difference was observed between the 0- and 12-hour in vitro magnetic actuation groups. Accordingly, the total duration of magnetic actuation only needs to be controlled within 24 hours (fig. S28). Longitudinal tracking of CD5^+^DC robot accumulation within tumors on days 7, 10, 13, 16, 19, and 22 post–tumor cell inoculation revealed persistent enrichment in tumor tissues, providing evidence for the long-acting immune-activating and cytotoxic effects of CD5^+^DC robots in vivo (Fig. 6H). Organ distribution kinetics of intravenously infused magnetically controlled CD5^+^DC robots (Fig. 6I) showed initial accumulation in the liver and spleen at 4 hours posttreatment, followed by distribution to the liver, lung, spleen, and kidney at 8 to 16 hours. By 32 hours, CD5^+^DC robots were primarily localized in the liver and kidneys, with trace residues remaining in the liver at 72 hours, indicating rapid metabolic clearance by systemic organs. Body weight analysis (Fig. 6J) revealed no significant weight loss across all groups. Histopathological examination of major organs (heart, liver, spleen, lung, and kidney) showed no obvious tissue damage, inflammatory infiltration, or other adverse reactions in the CD5^+^DC robot group and the CD5^+^DC robot* group, comparable to the PBS control group (Fig. 6K). To demonstrate that CD5^+^DC robots do not induce a severe inflammatory response during adoptive transfer, we divided mice into five groups and administered PBS, M1 macrophages, CD8^+^ T cells, neutrophils, and CD5^+^DC robots, respectively. Subsequently, we collected peripheral blood of mice and performed ELISA to detect IL-2, IL-6, TNF-α, and IFN-γ. The results showed that compared with the other groups, the levels of these inflammatory cytokines remained consistently low in the CD5^+^DC robot group (fig. S29). Collectively, these results validate the safety, tumor-reducing efficacy, biocompatibility, and bioabsorbability of CD5^+^DC robots for in vivo applications. Furthermore, we compared peripheral immune activation and intestinal inflammation in the PBS, CD5^+^DC robot, and magnetically actuated CD5^+^DC robot (CD5^+^DC robot*) groups. The results showed that while CD5^+^DC robots activated peripheral immune cells, magnetic targeted actuation confined inflammation to the tumor tissue in the CD5^+^DC robot* group, with no significant difference in peripheral immune cell proportions or aberrant activation compared with the PBS group (fig. S30). No obvious inflammatory damage was observed in the intestinal tissues of all three groups (fig. S31).

## DISCUSSION

The use of immune cell–based microbots for tumor-targeted therapy faces three critical challenges. First, massive adoptive transfer of ex situ cytotoxic immune cells easily triggers systemic inflammatory responses. Second, ex situ activation of cytotoxic immune cells drives their premature functional exhaustion. Third, ex situ cytotoxic immune cells fail to overcome the immunosuppressive microenvironment. To address these issues, we engineered a microrobot system using CD5^+^DCs. This system leverages natural CD5^+^DCs to actively phagocytose CCM@NPs, and PD-L1 on the surface of these CD5^+^DCs is preblocked (*56*). The CCM coating enhances the loading capacity of CD5^+^DCs for magnetic nanoparticles. Critically, this coating promotes CD5^+^DC activation, inducing the extension of dendritic tentacles and up-regulation of MHC-I/II antigen-presenting molecules and costimulatory molecules CD80/CD86. PD-L1 blocking relieves immunosuppressive and further enhances CD5^+^DC robots’ in situ immune activation capacity. This enables CD5^+^DC robots to activate various autologous immune cells in situ, efficiently mediating tumor cell killing. CD5^+^DC robots optimize immune cell–based microbots for tumor-targeted therapy through three key mechanisms: First, the in situ immune activation for tumor antigen presentation is strictly confined to the tumor site, which greatly reduces the probability of systemic inflammatory responses. Second, CD5^+^DC robot–mediated in situ activation of cytotoxic immune cells circumvents functional exhaustion from their ex vivo modification. Third, endowed with potent in situ immune activation capacity, these CD5^+^DC robots also replace DCs inactivated by the immunosuppressive TME. Both in vivo and in vitro experiments verify the in situ immunocyte activation of CD5^+^DC robots in tumor.

In the in vitro experimental system, CD5^+^DC robots exhibited significantly enhanced phagocytic efficiency and immune activation capacity, enabling efficient uptake of CCM@NPs. During fabrication, PD-L1 inhibitors were used to block PD-L1 on the basis of a previous study demonstrating the synergistic effect between CD5^+^DCs and immunosuppressants (*56*). In vitro motility assays revealed the CD5^+^DC robots’ excellent magnetically driven clustered motility and chemotactic motility—attributes that serve as the locomotor foundation for these robots to achieve in situ tumor activation. Immunofluorescence, flow cytometry, and ELISA results validated the molecular basis for the in situ activation capability of CD5^+^DC robots, namely the high expression of antigen-presenting molecules (MHC-I/II), costimulatory signals (CD80 and CD86), and cytokines. Cellular experiments directly confirmed their potent activation capacity toward T cells and macrophages.

In vivo validation demonstrated that CD5^+^DC robots retained highly efficient in situ immune activation capacity. Under external magnetic guidance and the action of tumor chemokines, CD5^+^DC robots efficiently accumulated near intestinal tumor sites. Flow cytometry analyses confirmed in situ activation of immune cells, mirroring in vitro findings. In vivo experimental results demonstrated that CD5^+^DC robots can remodel the tumor immunosuppressive microenvironment, achieve highly efficient in situ immune activation of T cells and macrophages, and do not induce systemic inflammatory responses. Within 19 days after CD5^+^DC robot treatment, the CD5^+^DC robots consistently exhibited strong fluorescent signals at the tumor site, which lays a solid foundation for their exertion of long-term in situ tumor immune activation. Notably, a single infusion of magnetically controlled CD5^+^DC robots led to significant tumor suppression. This indicates that CD5^+^DC robots induce sustained tumor-killing effects through the activation of in situ cytotoxic immune cells.

In clinical application scenarios, real-time imaging characterization of CD5^+^DC robots may still face technical bottlenecks. In this study, clearing-based real-time imaging technology and an in vivo fluorescence imaging system provided experimental evidence for the active targeted delivery of CD5^+^DC robots in the gastrointestinal tract of mice. This imaging technology enables tracking of the dynamic distribution of CD5^+^DC robots but is limited by spatial resolution and human tissue penetration depth, failing to achieve single-cell-level individual identification. Therefore, developing a high-performance real-time multiscale in vivo imaging platform is of great significance for the functional evaluation of CD5^+^DC robots. Notably, with the increase in clustering scale, the coordinated movement behavior of CD5^+^DC robot swarms regulated by RMFs and conical magnetic fields is expected to achieve in vivo visualization via existing imaging technologies, and this technical approach provides a potential strategy to address the challenge of real-time imaging.

## MATERIALS AND METHODS

### Materials

PLGA [lactide:glycolide = 50:50; MW (molecular weight) = 10,000 to 15,000 Da] was purchased from Sigma-Aldrich. Iron oxide (Fe_3_O_4_) nanoparticles (average diameter, 20 nm) were obtained from Aladdin. MC38 murine colorectal cancer cells were cultured in Dulbecco’s modified Eagle’s medium (Gibco) supplemented with 10% fetal bovine serum (Gibco) and 1% penicillin-streptomycin (Gibco) at 37°C in a 5% CO_2_ incubator. BALB/c and C57BL/6J mice (6 to 8 weeks old) were purchased from Charles River Laboratories and housed under specific pathogen–free conditions. Oxaliplatin, anti-mouse CD5, CD80, CD86, MHC-I, MHC-II, CD4, CD8, CD86 (M1 macrophage marker), and CD206 (M2 macrophage marker) antibodies were obtained from BD Biosciences. ELISA kits for IL-2, IL-6, IFN-γ, TNF-α, IL-1β, and perforin were purchased from R&D Systems.

### Fabrication of CD5^+^DC robots

CD5^+^DC robots were constructed in three sequential steps.

#### Synthesis of magnetic PLGA nanoparticles

Magnetic PLGA nanoparticles loaded with Fe_3_O_4_ were prepared via emulsion/solvent evaporation. Briefly, 100 mg of PLGA and 20 mg of Fe_3_O_4_ nanoparticles were dissolved in 2 ml of dichloromethane. This organic phase was emulsified in 10 ml of 2% polyvinyl alcohol aqueous solution via probe sonication (300 W, 3 min). The emulsion was stirred at 37°C for 4 hours to evaporate the solvent. Nanoparticles were collected by centrifugation (12,000*g*, 15 min), washed three times with distilled water, and lyophilized.

#### Coating with cancer cell membranes (CCM@NPs)

MC38 cells were harvested, and cell membranes were extracted via hypotonic lysis. Membrane vesicles were mixed with magnetic PLGA nanoparticles at a mass ratio of 1:5 and extruded through a 200-nm polycarbonate membrane (Avanti Polar Lipids) using a mini-extruder (LiposoFast) to form CCM-coated nanoparticles (CCM@NPs).

#### Phagocytosis by CD5^+^DCs

Bone marrow–derived DCs were isolated from C57BL/6J mice and cultured in RPMI 1640 (Gibco) supplemented with 10% fetal bovine serum, granulocyte-macrophage colony-stimulating factor (20 ng/ml), and IL-4 (10 ng/ml) for 7 days. CD5^+^DCs were enriched using CD5^+^ magnetic beads (Miltenyi Biotec) to >90% purity. Enriched CD5^+^DCs were cocultured with CCM@NPs (100 μg/ml) in the presence of IL-6 (10 ng/ml) and PD-L1 inhibitor (10 μg/ml) for 48 hours at 37°C and 5% CO_2_. CD5^+^DC robots were collected by centrifugation (300*g*, 5 min) and washed with PBS.

### Characterization of CD5^+^DC robots

#### Morphology and structure

TEM (JEOL JEM-2100) was performed to image PLGA NPs, CCM@NPs, and CD5^+^DC robots (stained with 2% uranyl acetate). CLSM (Zeiss LSM 880) was performed with fluorescein isothiocyanate–labeled CCM@NPs, DAPI-stained nuclei, and 1,1′-dioctadecyl-3,3,3′,3′-tetramethylindocarbocyanine perchlorate (Dil)–labeled DC membranes.

#### Zeta potential

Zeta potential of PLGA NPs, CCM vesicles, and CCM@NPs was measured using a Malvern Zetasizer Nano-ZS at 25°C.

#### Magnetic properties

Saturation magnetization of CD5^+^DC robots was analyzed using a SQUID (Quantum Design MPMS3) at 298 K.

#### Flow cytometry

CD5^+^DC robots were stained with fluorescent antibodies against CD5, CD80, CD86, MHC-I, and MHC-II. Samples were analyzed using a BD FACSCanto II flow cytometer, and data were processed with FlowJo software.

#### Cytokine detection

TNF-α and IL-6 levels in cell supernatants were quantified via ELISA according to the manufacturer’s protocol.

### Magnetic motion and chemotaxis assays

#### Individual locomotion

CD5^+^DC robots were suspended in PBS and subjected to an RMF (1 to 10 Hz, 10 mT) generated by a custom-built system. Motion trajectories were recorded via time-lapse microscopy (Olympus IX73), and velocities were analyzed using ImageJ software.

#### Swarm assembly and coordinated locomotion

To investigate collective behaviors, CD5^+^DC robots at a higher density (2 × 10^6^ cells/ml) were used. Chain-like swarm formation and upstream rolling motion were induced under an RMF (10 mT, 1 to 50 Hz). Ribbon-like swarm formation and downstream surfing motion were induced under a conical magnetic field (10 mT, 1 to 50 Hz). The transition between these swarm states was controlled by switching the field type. The assembly process, structural morphology, and coordinated movement of the swarms were recorded in real time. The velocity of swarms as a function of magnetic field frequency and the number of robots in a cluster (Ns) was quantified. The ability of chain-like swarms to adhere to the channel wall and achieve upstream motion against fluid flow was specifically assessed.

#### Chemotaxis

A microfluidic chamber (Ibidi) was used to establish a gradient of MC38 cell–conditioned medium. CD5^+^DC robot migration was imaged over 10 hours, and the migration distance was quantified using ImageJ.

### In vivo magnetic navigation and real-time imaging of CD5^+^DC robot swarms

#### Animal ethics

All experiments were approved by the Animal Care and Use Committee of Harbin Institute of Technology (approval no. 25YS-092) and conducted in compliance with laboratory animal care guidelines.

#### In vivo magnetic navigation system

A custom-built magnetic navigation system was used for in vivo experiments. The system comprised a pair of orthogonal electromagnetic coils capable of generating programmable RMFs and conical magnetic fields. The fields were controlled by a dual-channel power amplifier (AE-7250, NF Corporation) and a data acquisition card (PCI-6731, National Instruments) driven by a custom LabVIEW (National Instruments) program. This setup allowed for precise control of field strength (up to 20 mT), frequency (1 to 100 Hz), and orientation at the target region within the mouse abdomen.

#### Animal preparation for real-time intravital imaging

To enable high-resolution real-time imaging of CD5^+^DC robots within intestinal vasculature, mice underwent an abdominal clearing procedure. Briefly, anesthetized tumor-bearing mice were intravenously perfused with a tartrazine solution (50 mg/ml in saline) to reduce light scattering and enhance tissue transparency.

#### Real-time imaging setup

The magnetically guided locomotion and retention of CD5^+^DC robot swarms were monitored in real time using a stereomicroscope (SZX16, Olympus) equipped with a high-speed scientific complementary metal-oxide semiconductor camera (ORCA-Flash4.0, Hamamatsu). The anesthetized and cleared mouse was positioned on a custom stage, ensuring that the abdominal region was precisely centered within the magnetic field and the microscope’s focal plane.

#### In vivo locomotion

A hybrid actuation strategy integrating conical magnetic fields and RMFs was used. Potassium fluorescein was initially administered to enable real-time tracing and monitoring of the tumor location. Thirty minutes after tail vein injection of CD5^+^DC robots, a conical magnetic field (15 mT, 15 Hz) was applied to induce the robots to assemble into band-like clusters parallel to the vascular wall, which subsequently migrated toward and adhered to the vessel wall. Spatial positions of the CD5^+^DC robots and the tumor were simultaneously visualized via in vivo fluorescence imaging, and the magnetic field direction was calibrated toward the tumor region according to the connecting line between the centroids of their respective fluorescence signals. Thereafter, an RMF (20 mT, 10 Hz) was used to drive the robots to form chain-like clusters for tumor-targeted locomotion. During 0.5 to 12 hours postadministration, in vivo fluorescence imaging was performed every 30 s, and the magnetic field direction was continuously calibrated on the basis of the directional vector from the fluorescence signal of CD5^+^DC robots to that of the tumor tissue.

### In vitro immune activation assays

#### Tumor infiltration

Cy5-labeled CD5^+^DC robots were cocultured with murine colorectal tumor tissue for 24 hours. CLSM was used to visualize infiltration, and fluorescence intensity was quantified.

#### Immune cell activation

Tumor tissues were treated with PBS, CD5^−^DC, CD5^−^DC robot, CD5^+^DC, or CD5^+^DC robots for 24 hours. Single-cell suspensions were stained with antibodies against CD8 (T cells) and CD86 (M1 macrophages) and analyzed via flow cytometry.

#### Cytotoxicity assay

MC38 cells were cocultured with CD5^+^DC robots + PBMCs for 24 hours. Cell viability was assessed via PI/annexin V staining (Invitrogen) and imaged via fluorescence microscopy.

### In vivo therapeutic and biosafety assessment of CD5^+^DC robots

#### Orthotopic colorectal cancer model

C57BL/6J mice were injected with 1 × 10^6^ luciferase-expressing MC38 cells into the cecum to establish orthotopic tumors (5 days to develop).

#### Treatment groups

Once orthotopic tumor growth was confirmed, mice with matched baseline bioluminescence signals were randomly assigned into six groups (*n* = 5 per group) and received tail vein injections on day 5: PBS, CD5^−^DC, CD5^−^DC robot, CD5^+^DC, non–magnetically controlled CD5^+^DC robots (1 × 10^6^), or magnetically controlled CD5^+^DC robots (1 × 10^6^).

#### Immune profiling and transcriptomics

Tumors were harvested on day 20. Single-cell suspensions were analyzed via flow cytometry for CD8^+^ T cells and M1 macrophages. Total RNA from tumors was sequenced using an Illumina NovaSeq 6000. Differentially expressed genes were identified via DESeq2, and KEGG pathway enrichment was analyzed using Cluster Profiler.

#### Therapeutic efficacy and safety

Tumor progression was monitored via bioluminescence imaging (IVIS Spectrum, PerkinElmer) over 20 days. Survival curves were generated, and tumor volumes were measured at the end point. The body weight was recorded weekly, and major organs (heart, liver, spleen, lung, and kidney) were histologically analyzed via H&E staining. All image acquisition, quantitative measurements, and histological assessments were performed by investigators blinded to group allocation to minimize subjective observer bias.

### Statistical analysis

Data are expressed as the means ± SD from a minimum of three independent biological replicates. For comparisons among multiple groups, a one-way analysis of variance (ANOVA) with Tukey’s post hoc test was applied, assuming that data met the assumption of homogeneity of variances. All statistical tests were conducted using GraphPad Prism software (version 9.0.0). Differences were considered statistically significant at *P* < 0.05.
