# Supplementary material for: CD5+ dendritic cell robots mediated in situ immunocyte activation
Source: Sci Adv. 2026 Aug 12;12(33):eaee5305. doi: 10.1126/sciadv.aee5305 (PMC13464482; doi:10.1126/sciadv.aee5305)
Supplement: Supplementary file 1 [file sciadv.aee5305_sm.pdf]

Science Advances
AAAS

Supplementary Materials for

CD5$^+$ dendritic cell robots mediated in situ immunocyte activation

Chuanhua Li *et al.*

Corresponding author: Ying Cui, cuiying@hrbmu.edu.cn; Mengmeng Sun, mmsun@nus.edu.sg; Jie Zhao, jzhao@hit.edu.cn; Tianlong Li, tianlongli@hit.edu.cn

Sci. Adv. **12**, eaee5305 (2026)
DOI: 10.1126/sciadv.aee5305

The PDF file includes:

Text S1
Figs. S1 to S32
Legends for movies S1 to S14

Other Supplementary Material for this manuscript includes the following:

Movies S1 to S14

SUPPLEMENTARY TEXT

Text S1. External magnetic field setup

Magnetic generation consists of a three degrees of freedom Helmholtz coil, a pair of vertically arranged coils, multifunction data acquisition (DAQ NI-PCI-6259) and four single-channel output power amplifiers. The drive signal generated by DAQ was amplified by the amplifiers and excited the coil to generate magnetic fields. The Helmholtz coil generated a rotating magnetic field to achieve the propulsion and retention of microrobot. The giant three-orthogonal Helmholtz coils pair used the same drive system to navigate the locomotion of microrobot *in vivo*.

SUPPLEMENTARY FIGURES

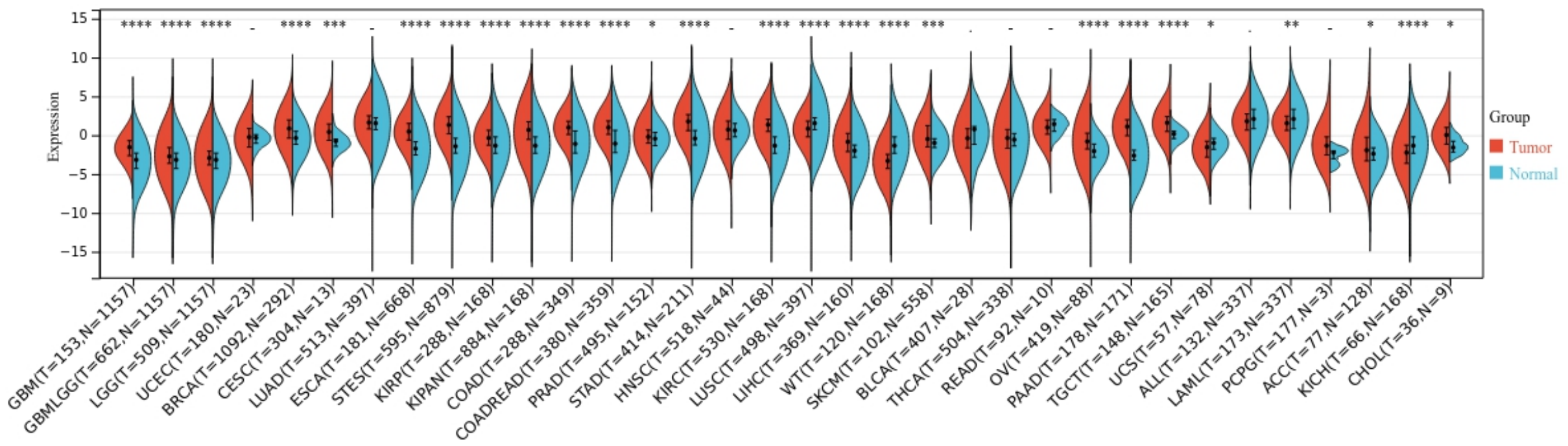

Fig. S1. Expression levels of CD5 in various tumors. Violin plots depict the distribution of CD5 expression in tumor (red) and matched normal (blue) tissues across multiple cancer types from The Cancer Genome Atlas (TCGA). Expression differences were assessed via the Wilcoxon rank-sum test, with significance indicated as P**** < 0.0001, P*** < 0.001, P** < 0.01 and P*< 0.05 (thresholds consistent with TCGA data analytical standards). Sample sizes (N) for each group are listed below the x-axis labels. CD5 expression exhibits substantial heterogeneity across tumor types, with prominent dysregulation in gliomas (GBM, LGG), gastrointestinal cancers (COAD, READ), and urological malignancies (KIRC, UCS), implying context-specific immunomodulatory roles in tumorigenesis.

A

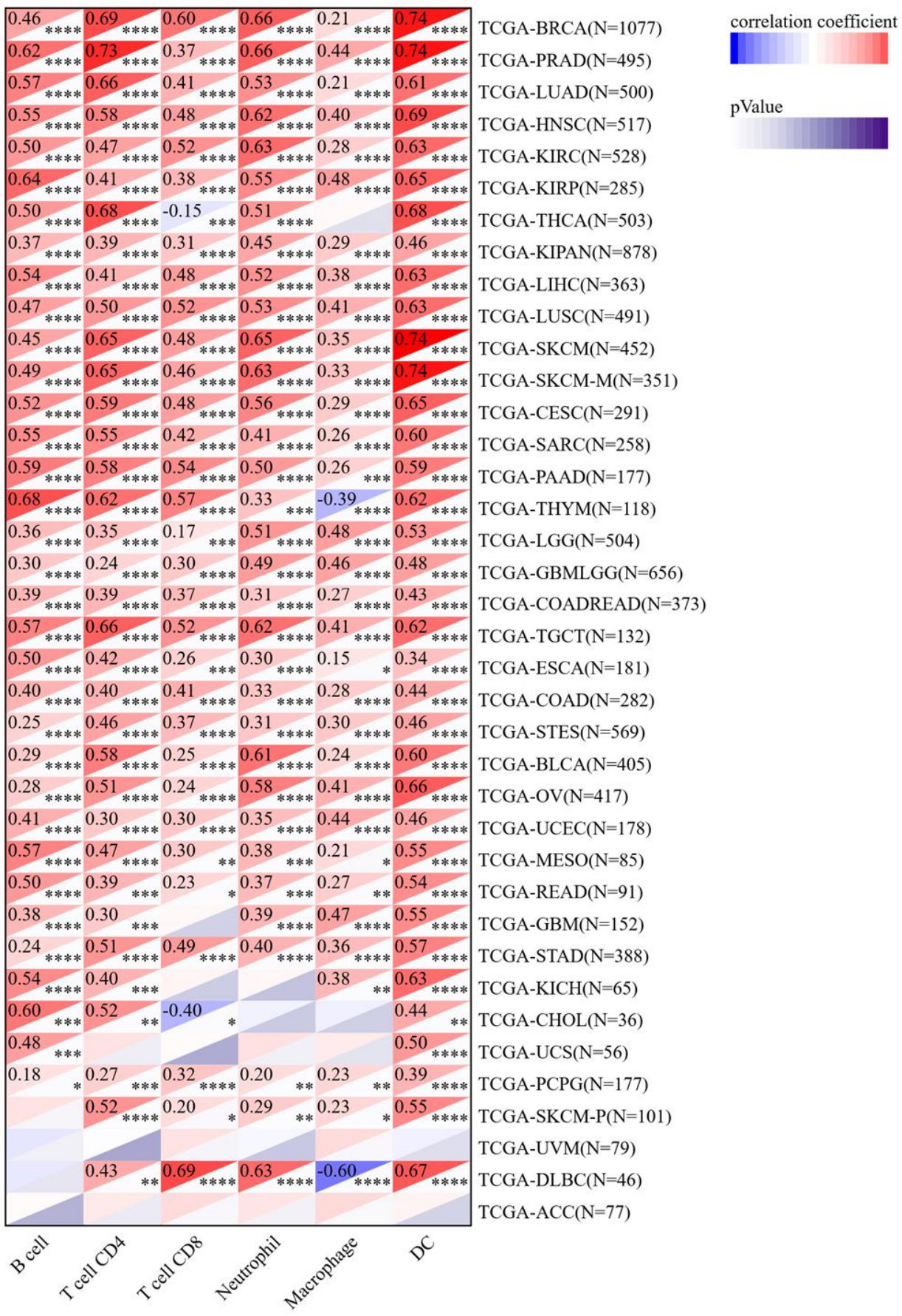

B

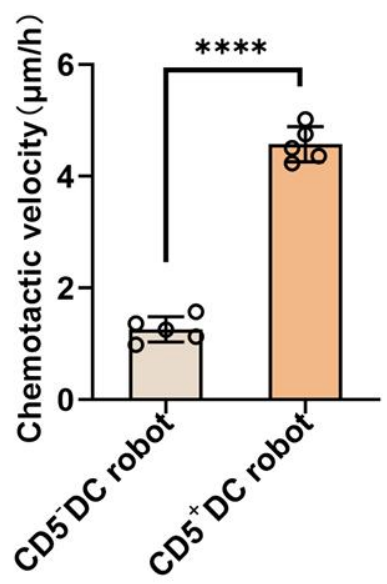

Fig. S2. The correlation between the expression level of CD5 in tumor tissues and immune cell infiltration. **(A)** Correlation heatmap depicts the association between CD5 expression in tumor tissues and infiltration levels of diverse immune cell subsets (including B cells, T cell subsets, macrophages, etc.) across cancer types from The Cancer Genome Atlas (TCGA). Correlation coefficients (r) are color-coded (blue indicates positive correlation, red indicates negative correlation), and statistical significance is marked as * ($P < 0.05$), ** ($P < 0.01$), and *** ($P < 0.001$) based on Spearman's rank correlation test. CD5 expression shows prominent positive correlations with effector T cell subsets (e.g., $CD8^+$ T cells, T follicular helper cells) in breast (BRCA) and lung adenocarcinoma (LUAD), while negative correlations with immunosuppressive populations (e.g., M2 macrophages) are observed in gliomas (GBM, LGG). These findings imply CD5-dependent modulation of tumor immune microenvironments, laying a foundation for understanding $CD5^+$ dendritic cell robots' immunomodulatory mechanisms. **(B)** Experimental verification of the chemotaxis velocity difference between $CD5^-$DC robots and $CD5^+$DC robots. *$P < 0.05$; **$P < 0.01$; ***$P < 0.001$, ****$P < 0.0001$.

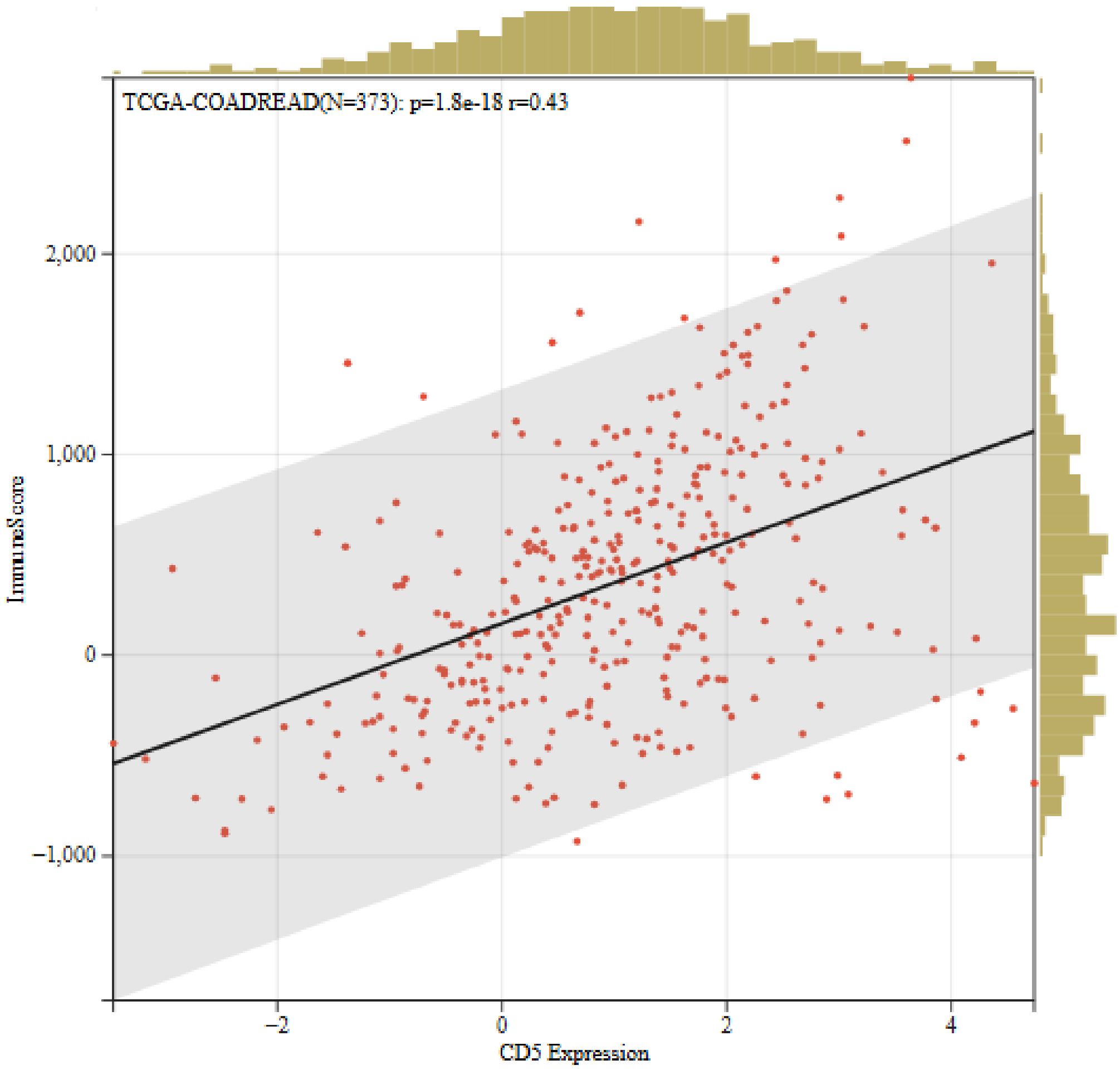

Fig. S3. Scatter plot of the correlation between CD5 and immune score in all types of human tumors. Scatter plot illustrates the correlation between CD5 expression and immune score across multiple tumor types from The Cancer Genome Atlas (TCGA, COAD/READ cohort, N=373). A linear regression model (black line) and 95% confidence interval (gray shading) were used to assess the association, with a Spearman's correlation coefficient (r) of 0.43 and statistical significance of $p=8.1\times10^{-18}$. CD5 expression exhibits a robust positive correlation with immune score, indicating that higher CD5 levels are associated with increased immune cell infiltration across colorectal cancer subtypes. This finding aligns with CD5's role in modulating immune microenvironments and supports the rationale for leveraging $CD5^+$ dendritic cell robots to enhance antitumor immunity.

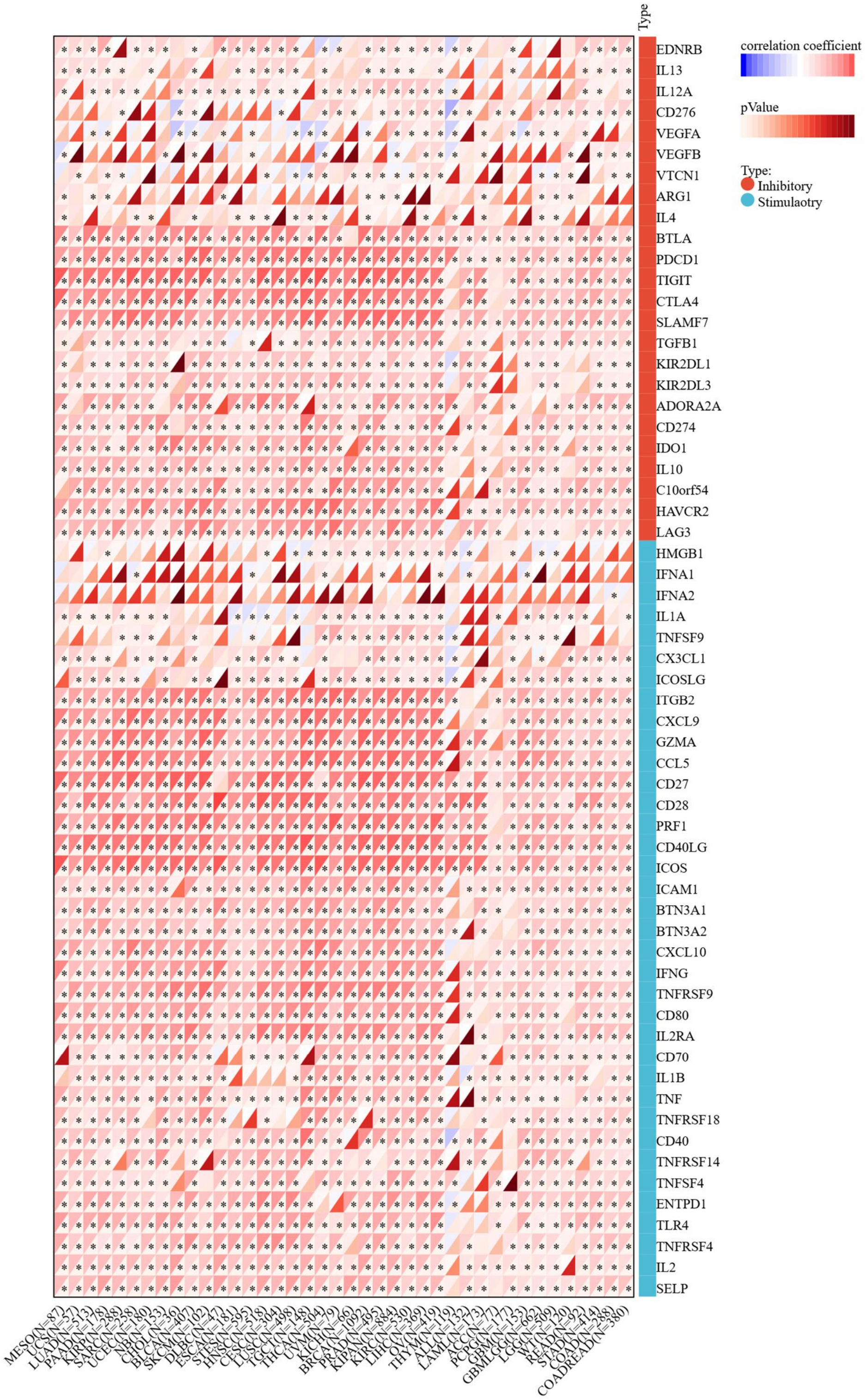

Fig. S4. Heatmap of the correlation between CD5 and immune checkpoints in all types of human tumors. CD5 expression exhibits notable positive correlations with multiple immune checkpoints in gastrointestinal and lung cancers, implying CD5-dependent regulation of immune evasion mechanisms and supporting the potential of combining $CD5^+$ dendritic cell robots with checkpoint blockade therapies.

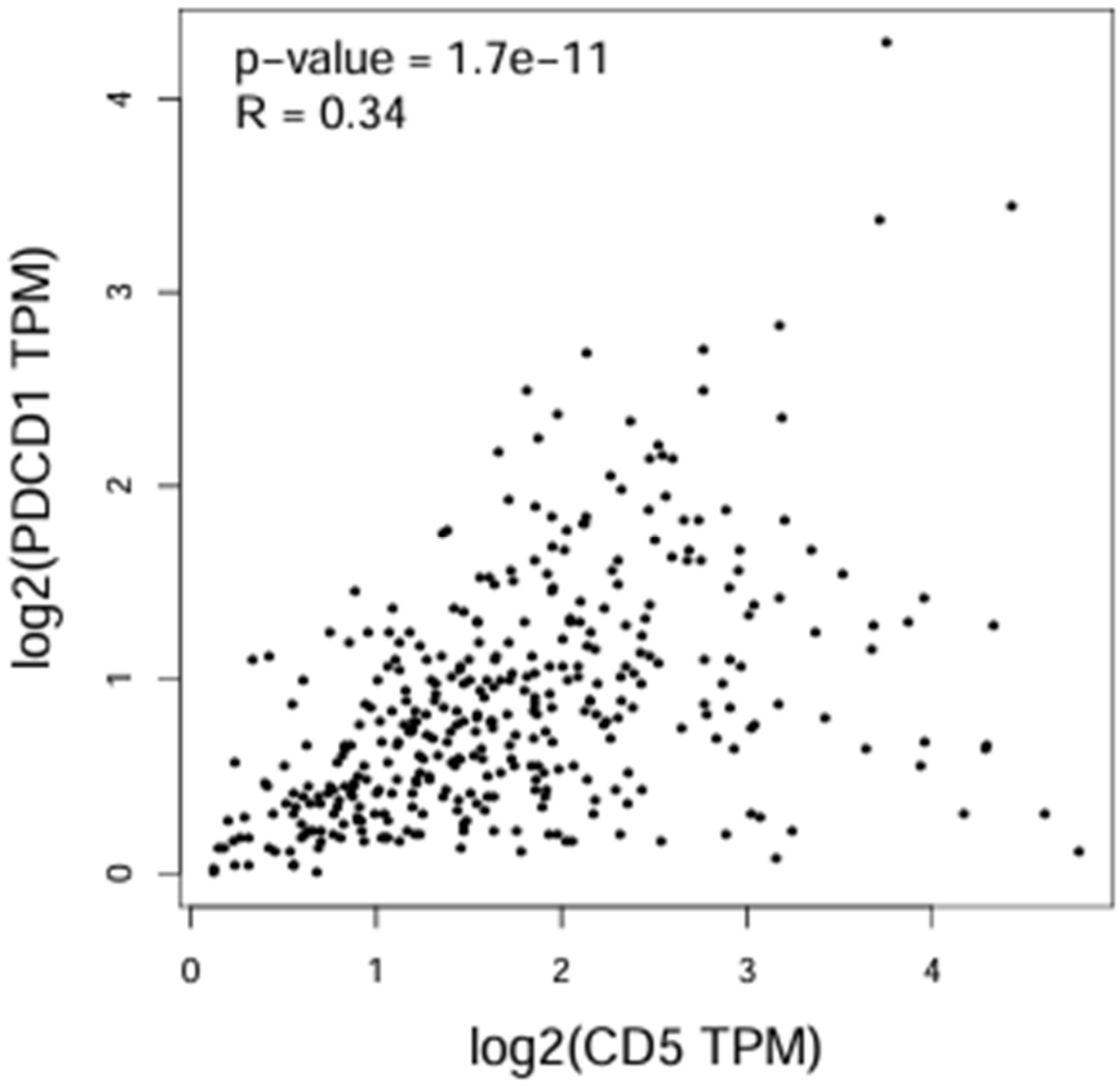

Fig. S5. Analysis of the correlation between the expression level of CD5 gene and PDCD1 (the immune checkpoint gene corresponding to PD-1) in human colorectal cancer. Scatter plot illustrates the correlation between CD5 and PDCD1 (PD-1–encoding gene) expression in colorectal cancer from The Cancer Genome Atlas (TCGA). A Spearman's rank correlation test was performed, yielding a correlation coefficient (R) of 0.34 and statistical significance ($p = 1.7\times10^{-11}$). CD5 expression exhibits a positive correlation with PDCD1, implying $CD5^{+}$ cells are associated with PD-1–mediated immune regulation in colorectal cancer. This finding supports the potential synergy of combining $CD5^{+}$ dendritic cell robots with PD-1 blockade, as CD5-driven immune activation may augment checkpoint inhibition efficacy.

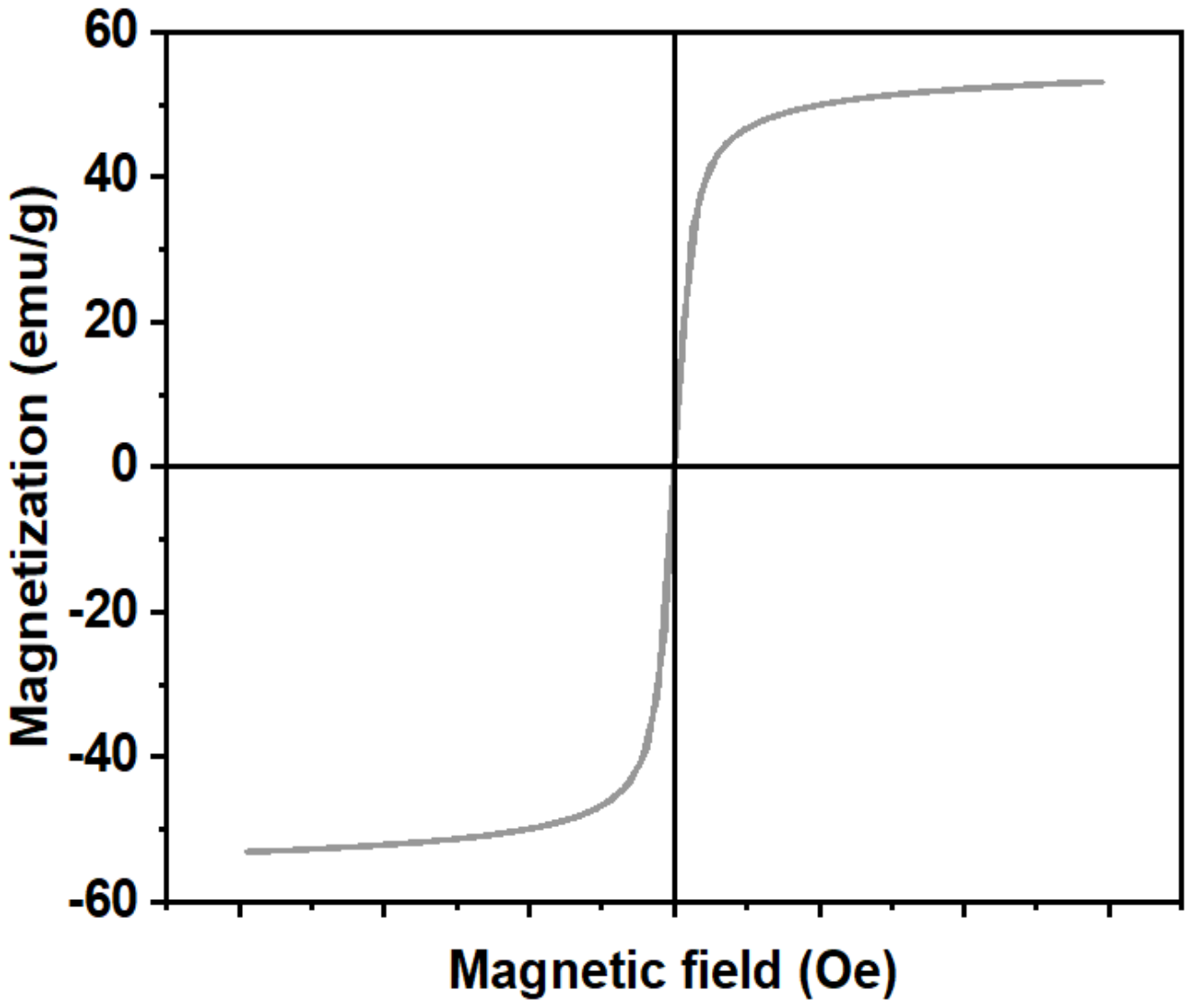

Fig. S6. Hysteresis curves of MC38 CCM@NPs. Magnetic hysteresis loop characterizes the magnetization (emu·g^{-1}) of MC38 tumor cell membrane-coated PLGA nanoparticles (CCM@NPs) as a function of applied magnetic field (Oe). The curve exhibits superparamagnetic behavior, with negligible hysteresis and a saturation magnetization that enables efficient response to external magnetic fields. This magnetic property is critical for the precise magnetic manipulation of CD5^{+}DC robots, supporting their targeted accumulation in tumor tissues as demonstrated by *in vivo* magnetic targeting assays, and thus facilitating the development of magnetically steerable immunotherapeutic agents.

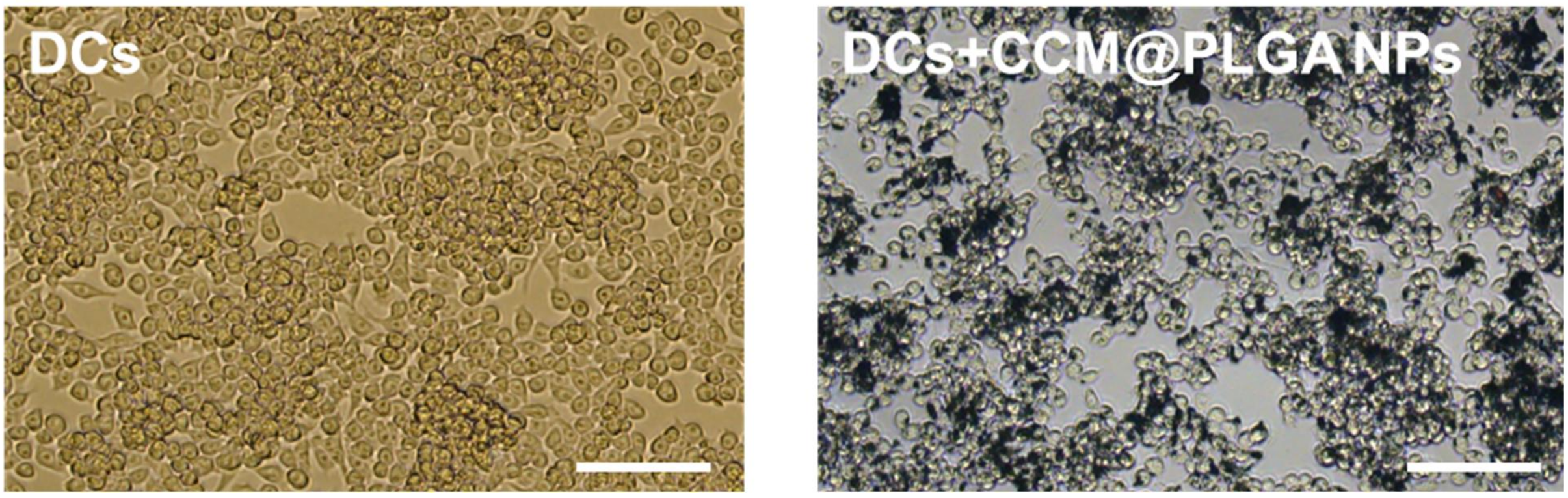

Fig. S7. Brightfield microscope images of CD5$^+$ dendritic cells before and after phagocytosis of CCM@PLGA nanoparticles (NPs). Brightfield microscopy images depict CD5$^+$DCs without (left) and with (right) phagocytosis of MC38 CCM@NPs. After incubation, CD5$^+$DCs exhibit intracellular black particles corresponding to NPs, indicating efficient phagocytic uptake. Scale bars = 100 μm. This result confirms CD5$^+$DCs can effectively engulf CCM@NPs, ensuring antigen loading and subsequent immune activation—an essential prerequisite for the functional design of CD5$^+$DC robots in tumor immunotherapy.

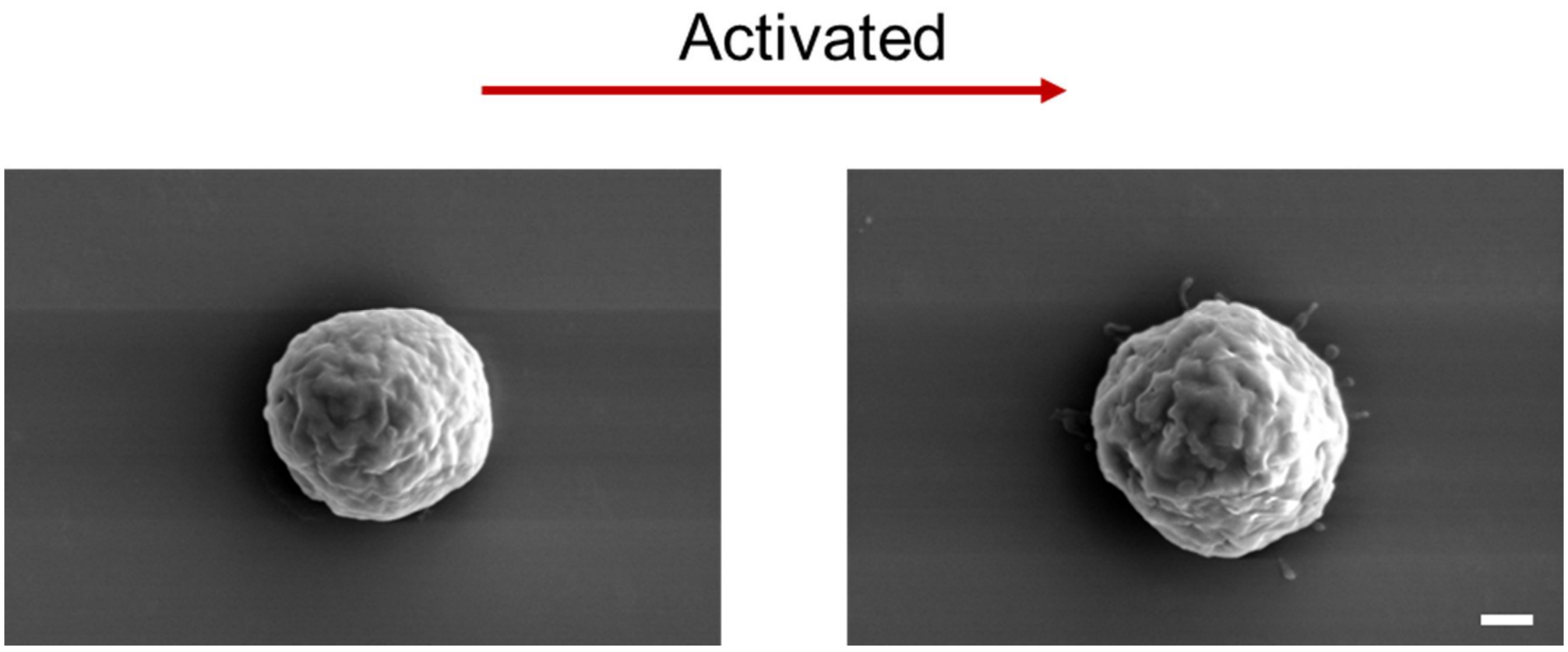

Fig. S8. SEM of CD5$^+$ dendritic cells before(right) and after activation(left). Scanning electron microscopy (SEM) images illustrate CD5$^+$DCs in resting (left) and activated (right) states. Activated CD5$^+$DCs display abundant dendritic protrusions, while resting CD5$^+$DCs show a relatively smooth surface. This morphological shift signifies DC maturation, where expanded dendritic structures promote antigen presentation and T cell engagement—critical for the immunostimulatory capacity of CD5$^+$DC robots in tumor immunotherapy. The structural change aligns with upregulated expression of co-stimulatory molecules (e.g., CD80, CD86) and MHC-II (as confirmed by flow cytometry, consistent with Fig. 1), highlighting the functional maturation of DCs upon activation. Scale bar,1 μm.

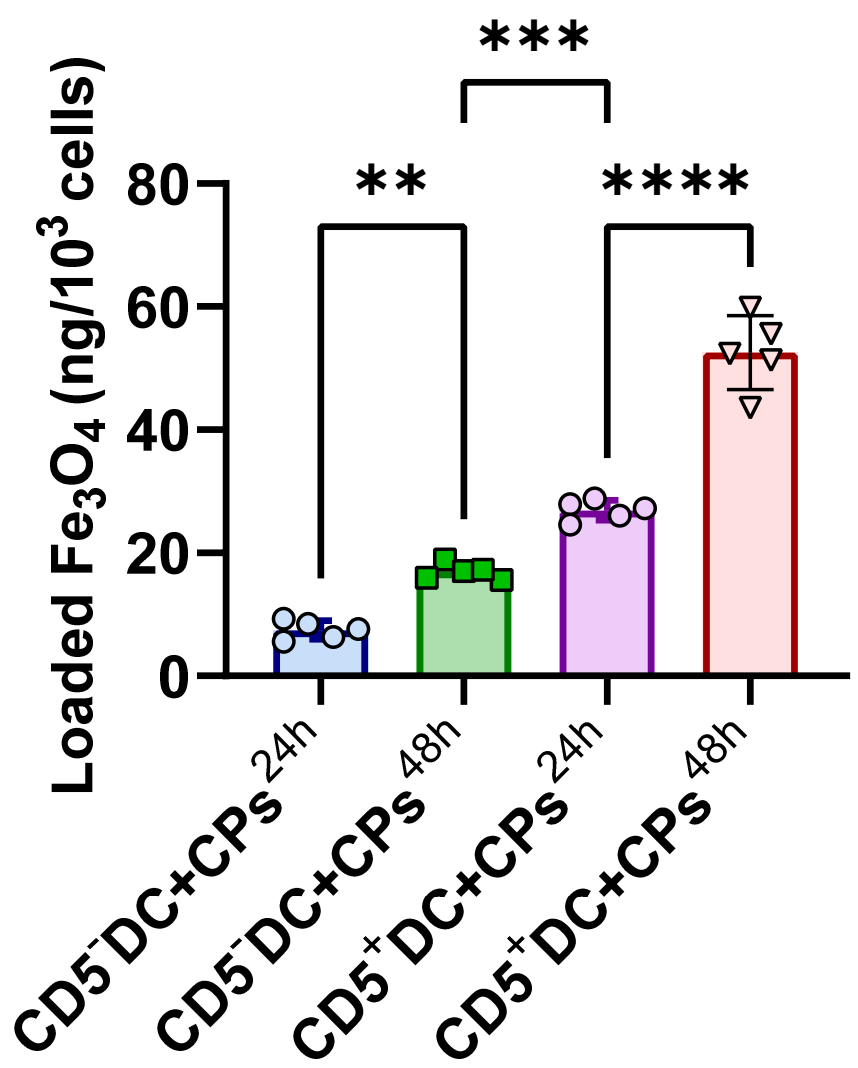

Fig. S9. Fe_3O_4 loading capacity in 10^3 $CD5^{-}DCs$ or $CD5^{+}DCs$ after co-incubation with CCM@NPs for 24 h and 48 h, respectively. *$P < 0.05$; **$P < 0.01$; ***$P < 0.001$, ****$P < 0.0001$.

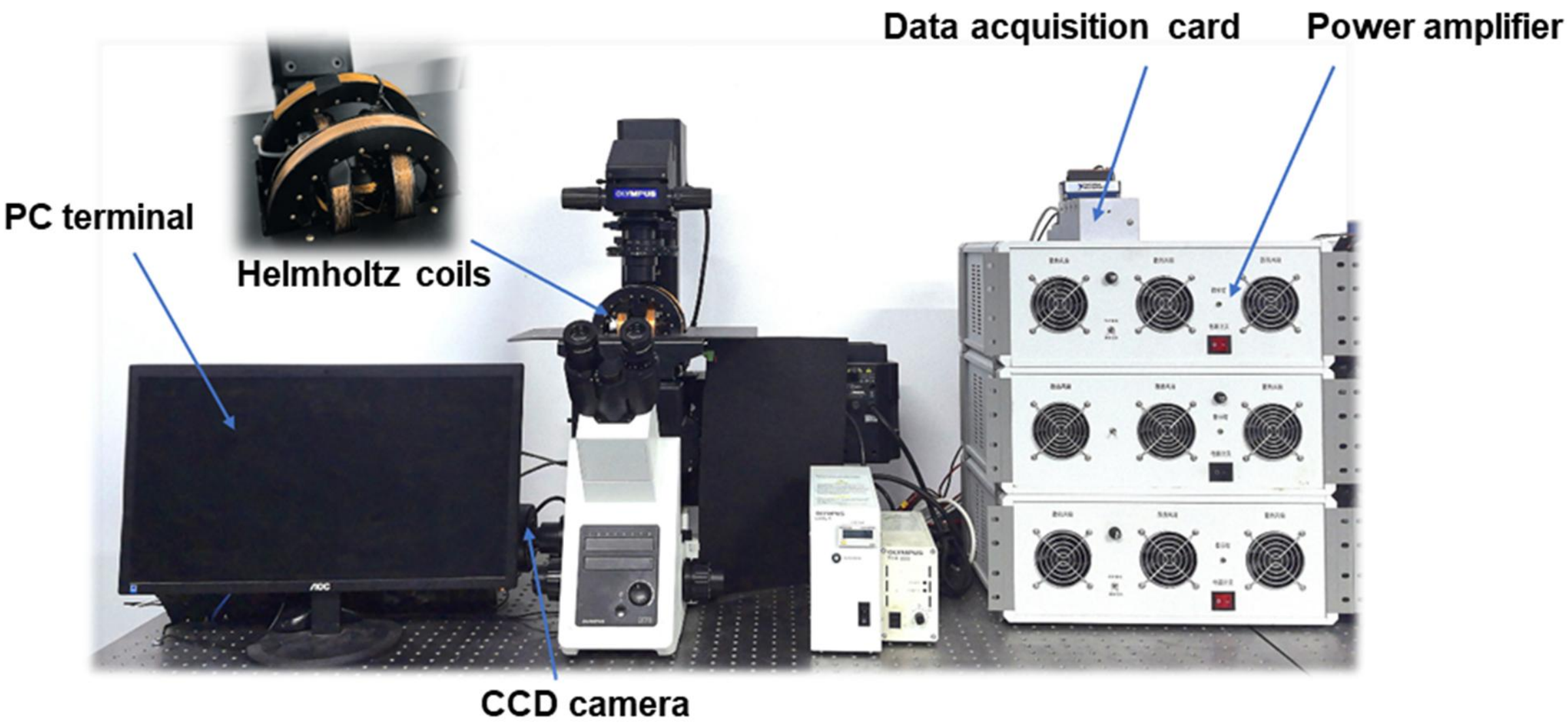

Fig. S10. Integral experimental system. The system containing PC terminal, Helmholtz coils, CCD camera, Data acquisition card and Power amplifier. The system comprises a PC terminal (for control and data processing), Helmholtz coils (for generating tunable magnetic fields), a CCD camera (for real-time imaging of $CD5^{+}$DC robot motion), a data acquisition card, and a power amplifier (for magnetic field signal conversion and amplification). This integrated platform enables precise magnetic steering of $CD5^{+}$DC robots (via adjustable field frequency/amplitude) and synchronous visualization of their dynamic behaviors, which is critical for optimizing the magnetic targeting strategy of $CD5^{+}$DC robots in *in vitro* and *in vivo* studies (consistent with the magnetic control assays in Fig. 3).

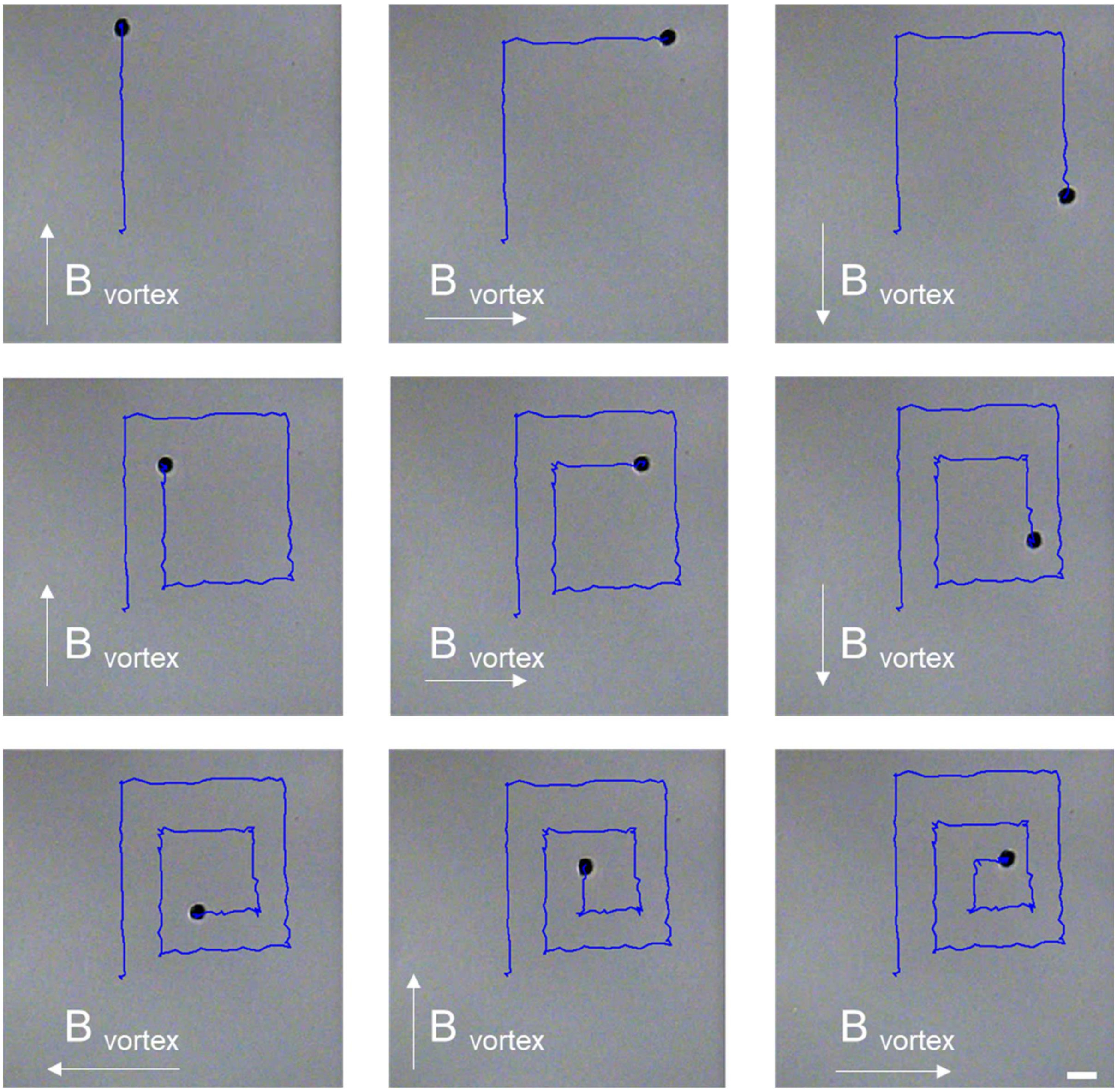

Fig. S11. Time-lapse bright-field images of the CD5^{+}DC robot's movement along a preset polyline trajectory under a rotating magnetic field. Time-lapse bright-field images depict the programmable motion of CD5^{+}DC robots under a rotating magnetic field (B vortex, direction indicated by arrows). The CD5^{+}DC robot traverses preset polyline trajectories (blue lines), showcasing stepwise navigation via magnetic field tuning. This assay validates the magnetic programmability of CD5^{+}DC robots—they can be precisely guided along custom trajectories by adjusting the magnetic field's rotation direction and frequency. Such controllability is critical for navigating biological barriers *in vivo*, enabling targeted delivery of CD5^{+}DC robots to tumor sites. The capacity to follow preset paths also underpins potential applications in spatially precise immunotherapy, where CD5^{+}DC robots could be directed to specific tumor subregions for localized immune activation. Scale bar, 20 μm.

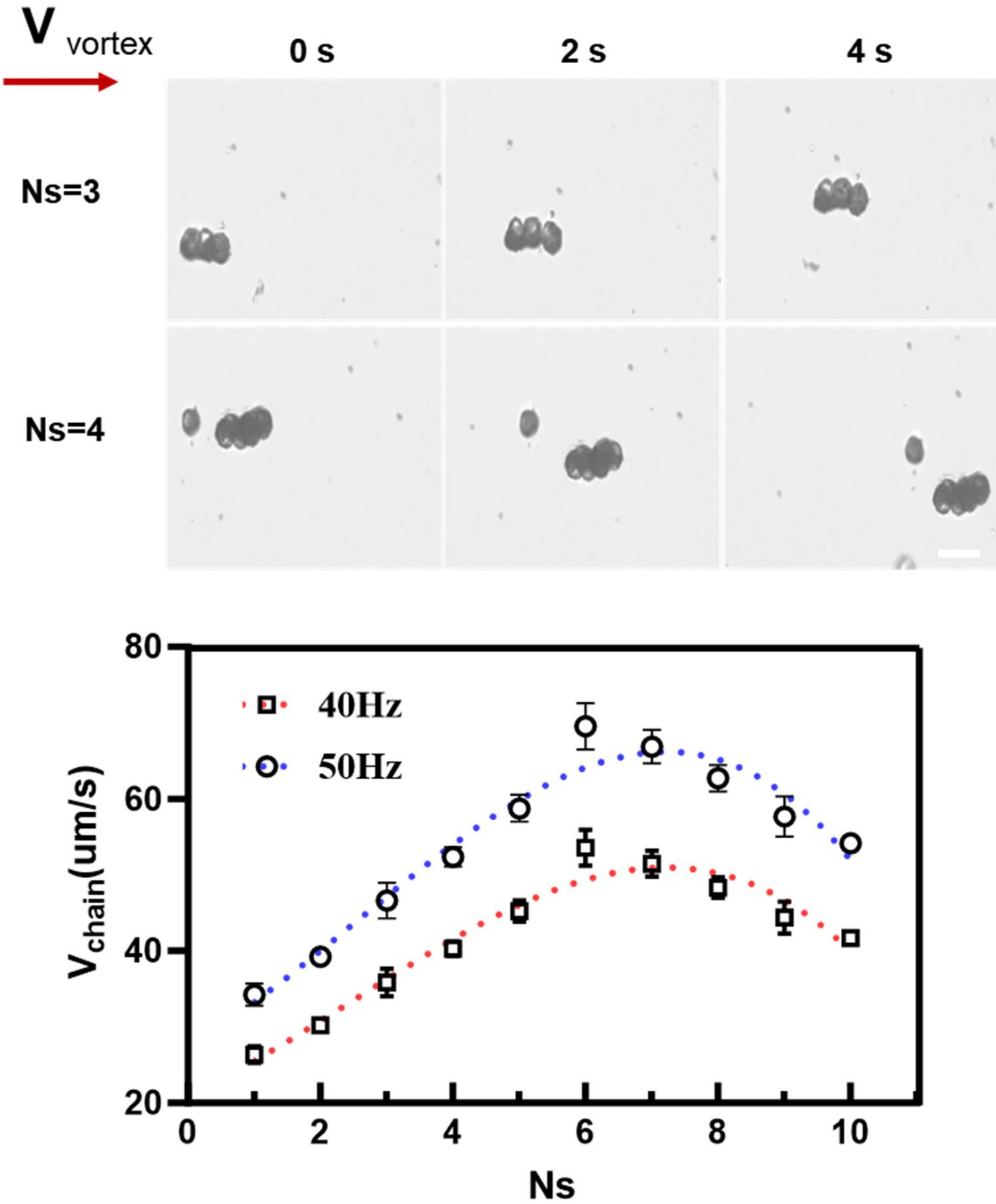

Fig. S12. Bright-field images of the movement of chain-like clusters of different lengths under a rotating magnetic field and their velocity statistics. This analysis elucidates the tunability of CD5$^+$DC robot cluster motion: chain velocity is synergistically regulated by cluster length and magnetic field frequency. The peak velocity at intermediate Ns (6–8) arises from a balance between magnetic torque transmission and hydrodynamic drag, enabling optimized *in vivo* targeting efficiency. Scale bar, 20 μm.

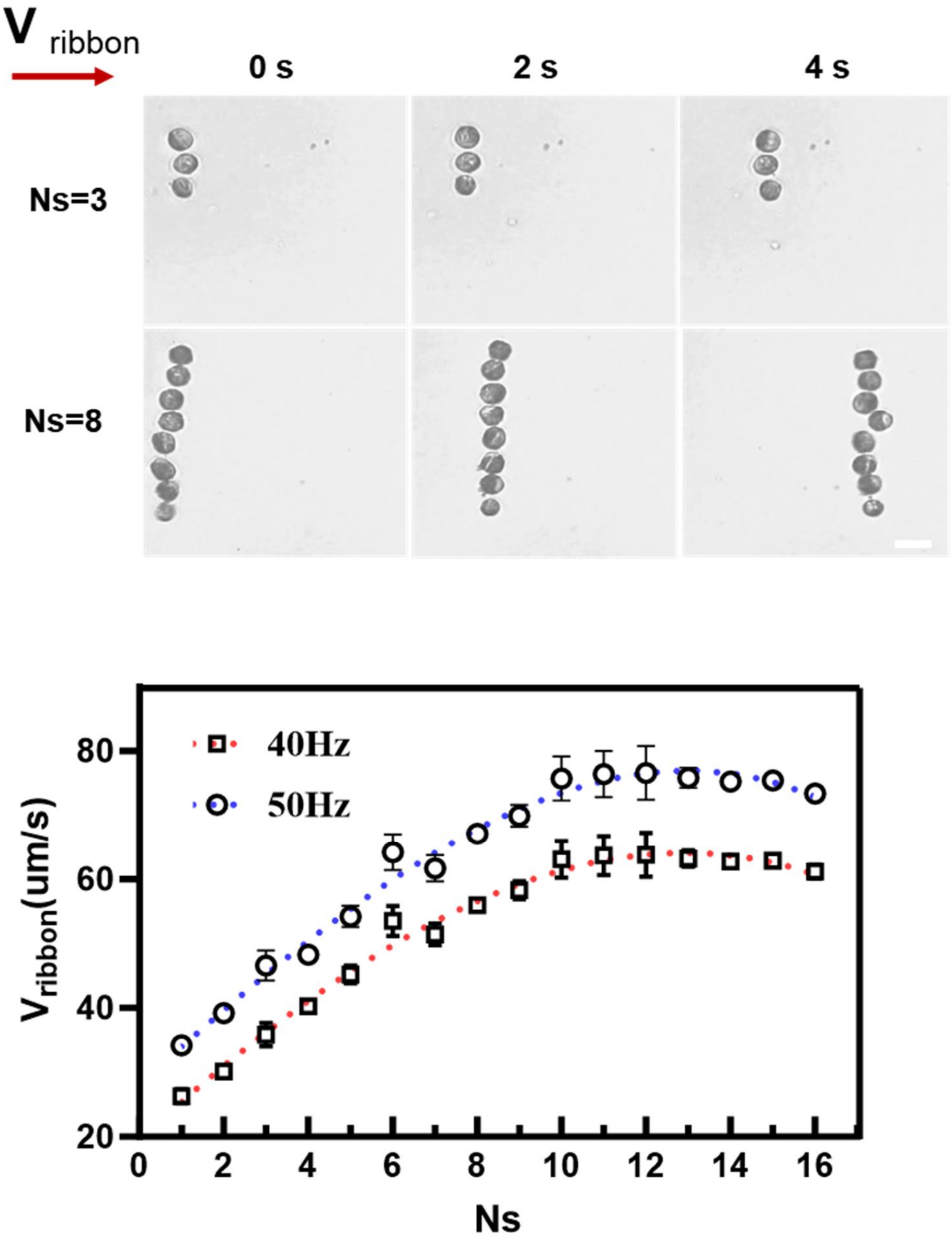

Fig. S13. Bright-field images of the movement of ribbon-like clusters of different lengths under a conical magnetic field and their velocity statistics. This analysis reveals the tunable motion dynamics of ribbon-like $CD5^{+}DC$ robot clusters: their velocity is positively correlated with cluster length and magnetic field frequency, a behavior rooted in the conical field's torque distribution across the ribbon structure. Compared to chain-like clusters, ribbon-like configurations enable higher sustained velocities, making them advantageous for traversing wide interstitial spaces in vascular. This morphological and dynamical versatility supports the rational design of $CD5^{+}DC$ robots for diverse *in vivo* microenvironments. Scale bar, 20 μm.

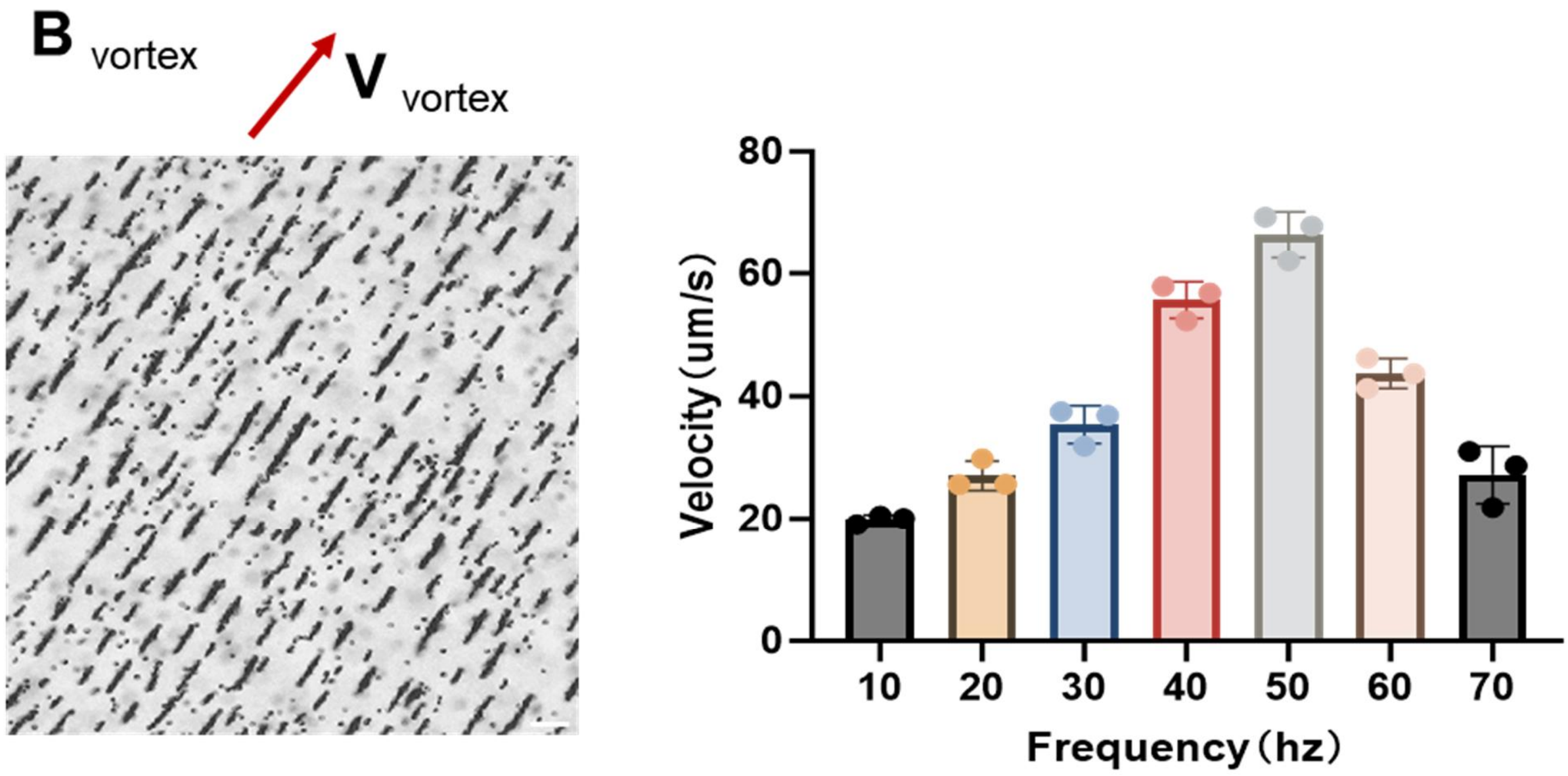

Fig. S14. Bright-field images of the movement of large-scale chain-like clusters under a rotating magnetic field and velocity statistics at different frequencies. This analysis illustrates the frequency-dependent motion of large-scale CD5^{+}DC robot clusters: velocity increases with magnetic field frequency up to 50 Hz and then decreases at higher frequencies. The scalability of chain-like clusters under rotating magnetic fields supports their potential for *in vivo* applications, where large-scale immune cell recruitment and tumor penetration are needed. This finding highlights the tunability of CD5^{+}DC robot motion for precision immunotherapy. Scale bar, 100 μm.

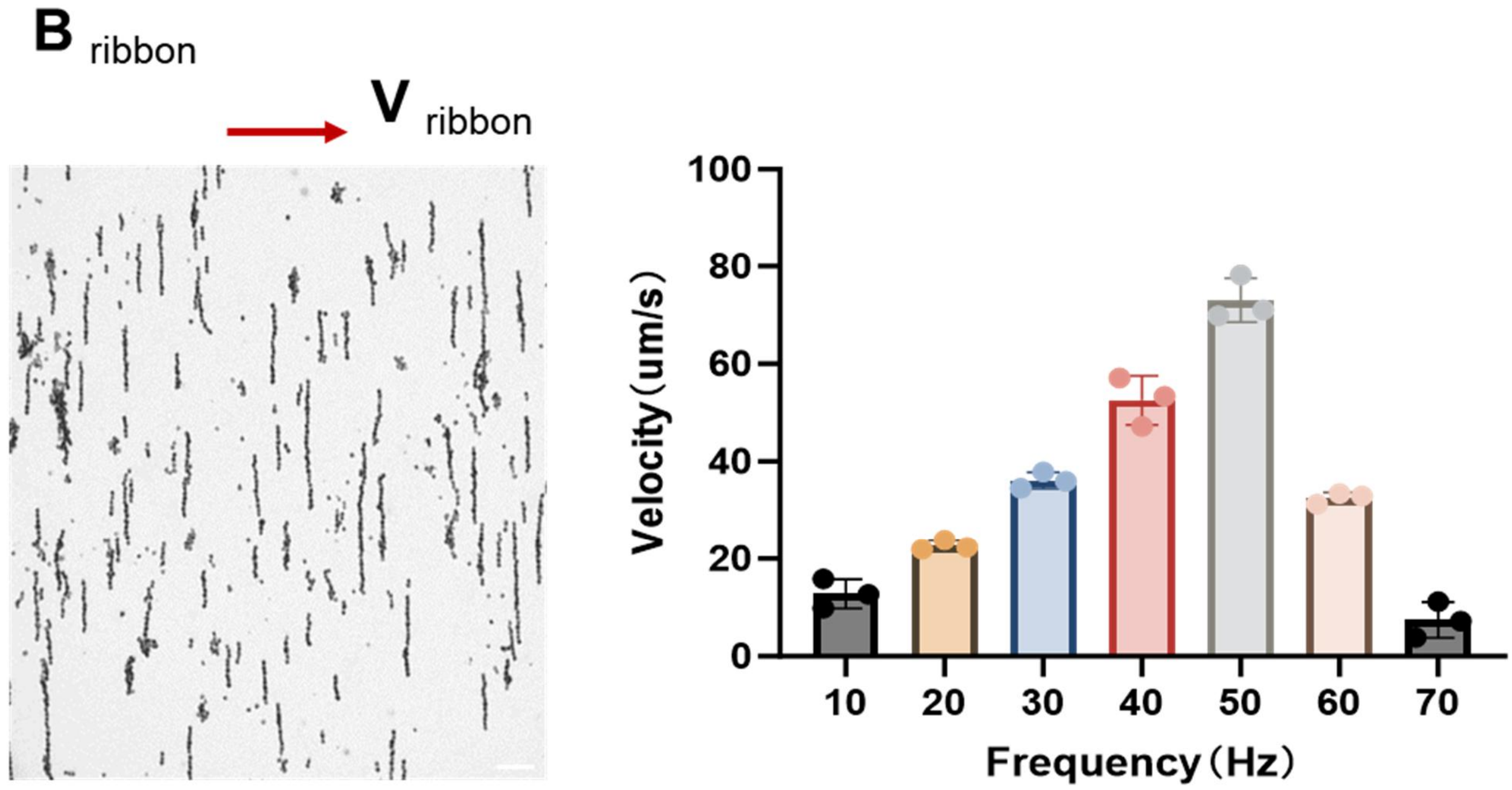

Fig. S15. Bright-field images of the movement of large-scale ribbon-like clusters under a conical magnetic field and velocity statistics at different frequencies. This analysis illustrates the frequency-dependent motion of large-scale CD5$^+$DC robot ribbon-like clusters: velocity increases with magnetic field frequency up to 50 Hz and then decreases at higher frequencies. The scalability of ribbon-like clusters under conical magnetic fields supports their potential for *in vivo* applications. This finding complements the length-dependent dynamics of ribbon-like clusters (Fig. S13) and highlights the tunability of CD5$^+$DC robot motion for precision immunotherapy, enabling rational design of magnetic control strategies tailored to diverse tumor microenvironments. Scale bar, 100 μm.

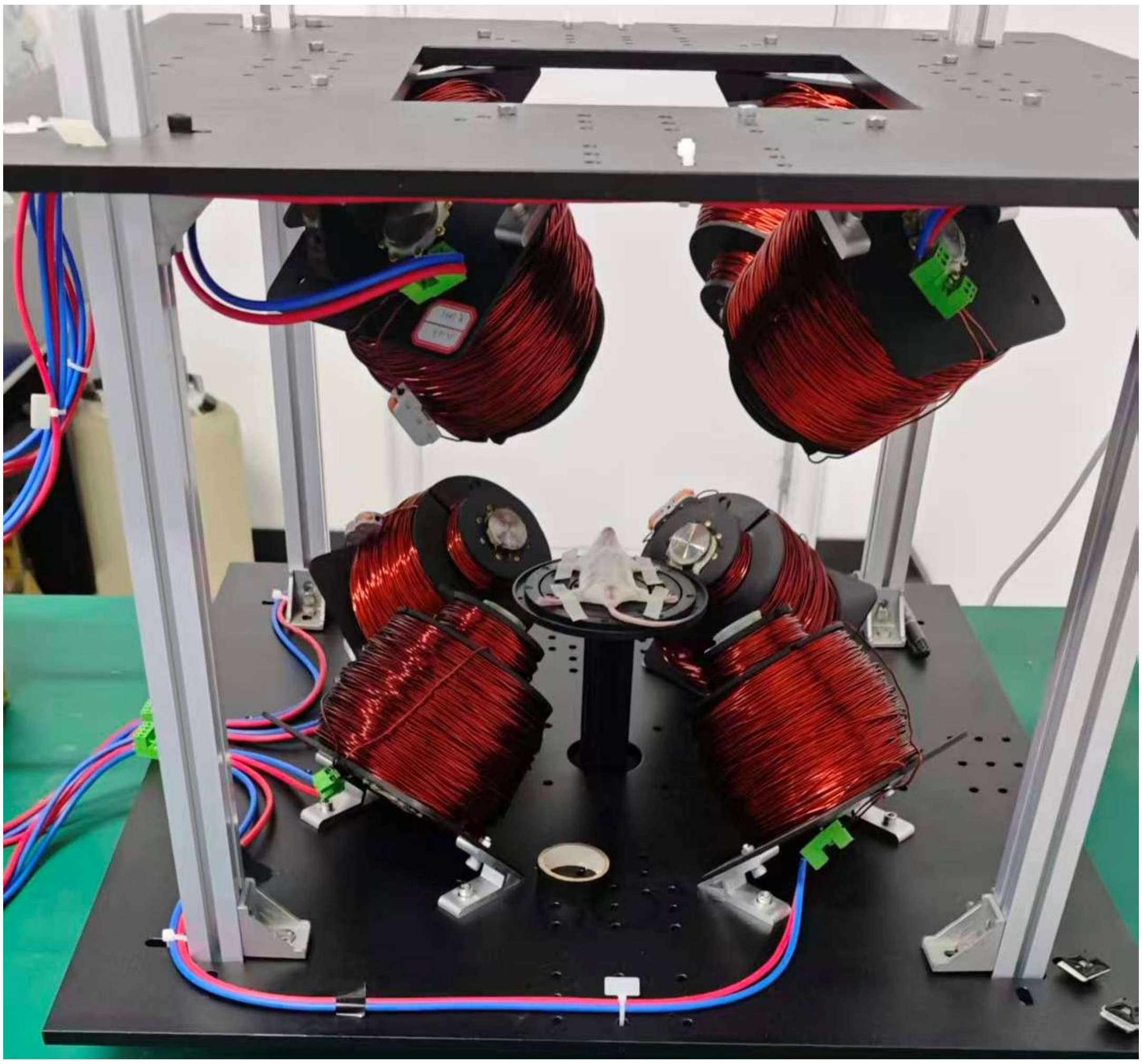

Fig. S16. ***In vivo* magnetic control coils for small animals.**

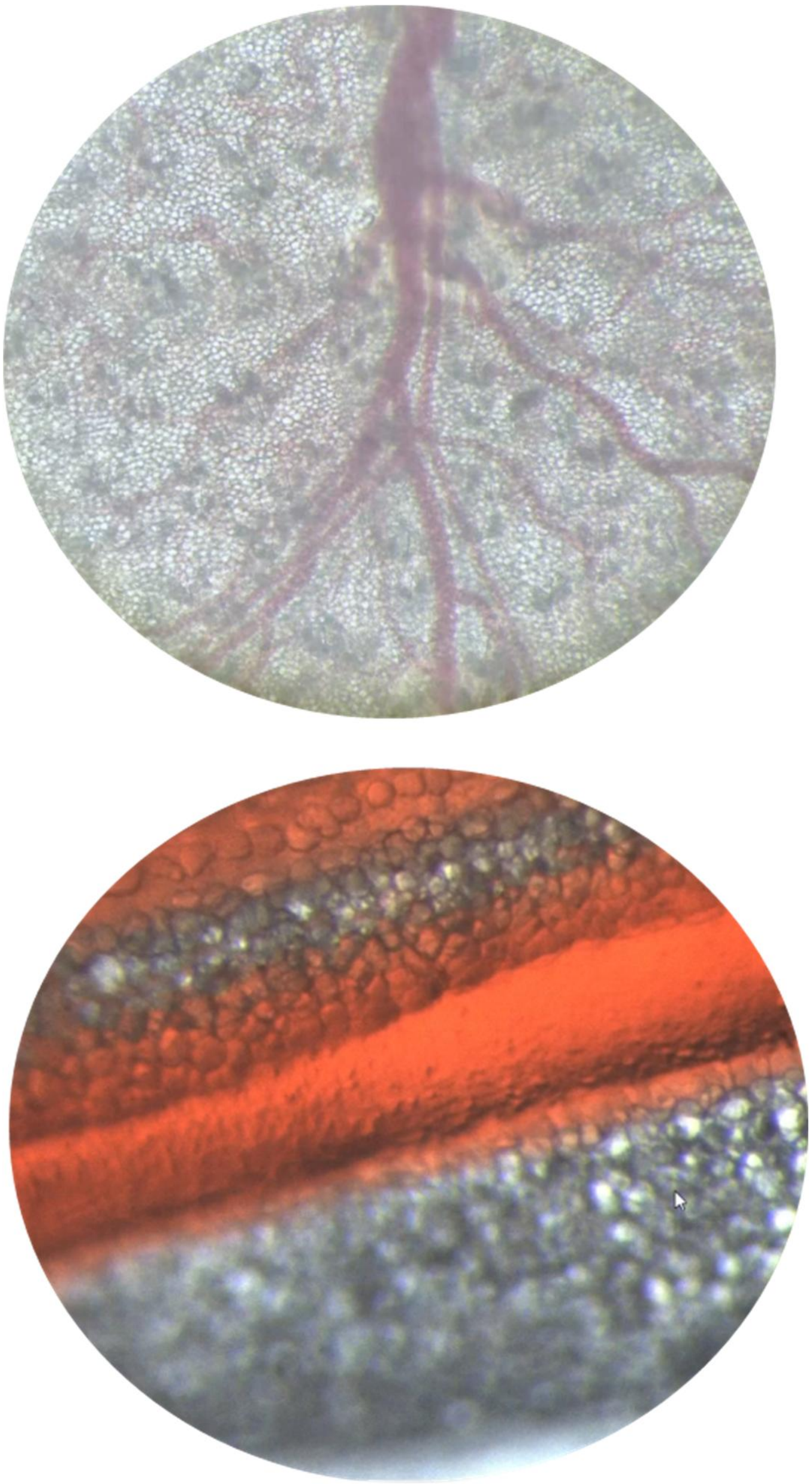

Fig. S17. Stereomicroscopic images of the epithelial blood vessels and intestinal blood vessels in mice after tartrazine-based clearing. A concise protocol for tartrazine-based clearing of mouse abdominal tissues involves the following key steps: First, prepare 1%-5% (w/v) filtered tartrazine aqueous solution and sterile saline. Anesthetize SPF-grade C57BL/6 mice (20-25 g) with intraperitoneal 1% pentobarbital sodium (50 mg/kg), then expose abdominal intestinal tissues via midline skin dissection. Next, infiltrate the exposed tissues with 50-100 μL tartrazine solution, incubate at 37℃ for 15-30 min (supplementing solution every 5min), and rinse 3 times with saline to remove residuals. Finally, verify tissue transparency using a stereomicroscope for clear intestinal vessel visualization, followed by real-time imaging if required.

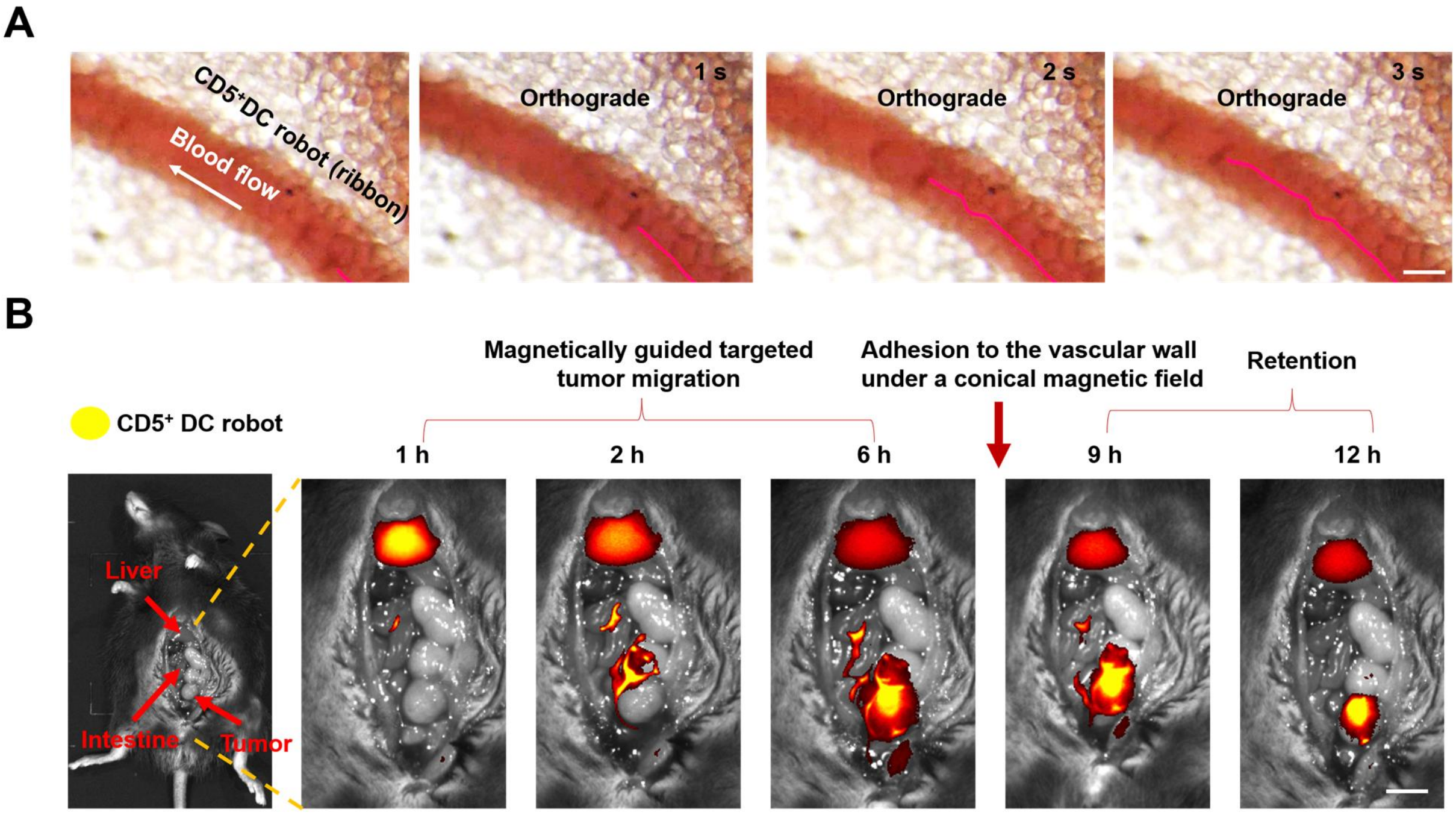

Fig. S18. CD5$^+$DC robots exhibit downstream locomotion and retention under a conical magnetic field. (A) Time-lapse images of ribbon-like clusters formed perpendicular to the vascular wall under a conical magnetic field (15 mT, 15 Hz) moving orthogradely with blood flow. Scale bar, 200 µm. **(B)** Time-lapse images of CD5$^+$DC robots accumulated in tumor blood vessels *in vivo* showing stable retention for over 6 hours under intravital fluorescence imaging.

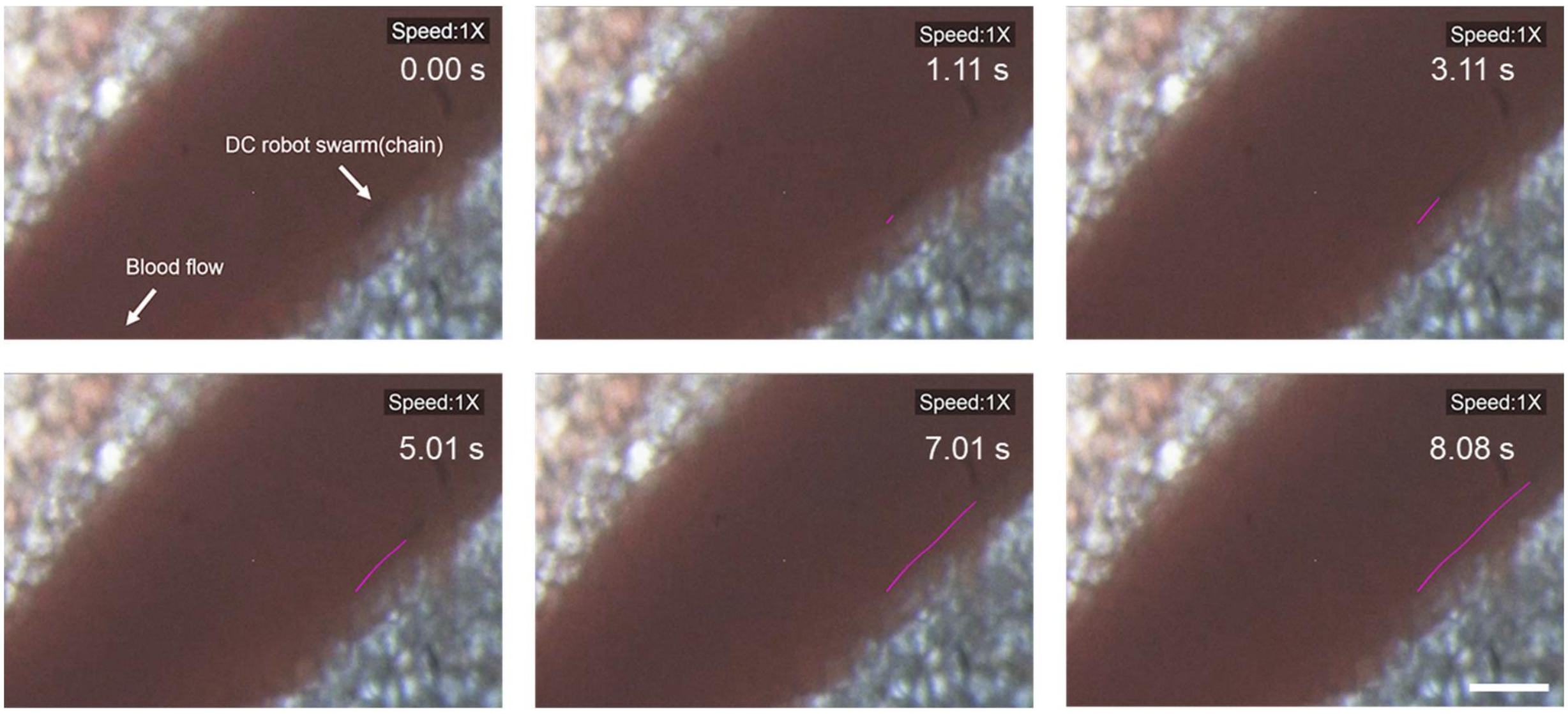

Fig. S19. Stereomicroscopic time-lapse images of chain-like CD5$^+$DC robot swarms adhering to the vascular wall for retrograde movement in *in vivo* cleared mice. Scale bar, 400 μm.

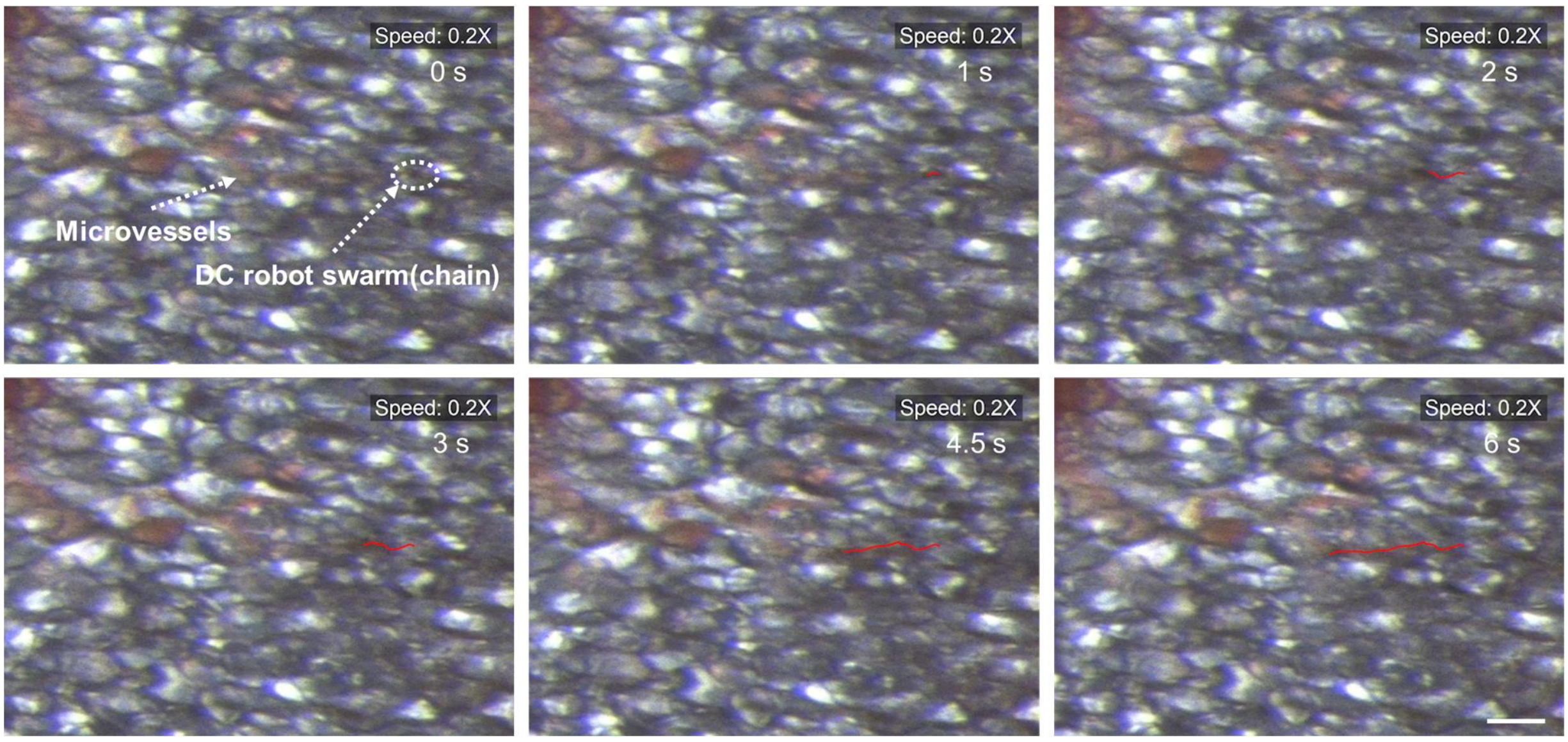

Fig. S20. Chain-like CD5$^+$DC robot swarm can traverse narrow microvessels (approximately 20 µm in width) without causing blockage, enabling them to reach distant tumor tissues via the systemic circulatory system. Scale bar, 100 µm.

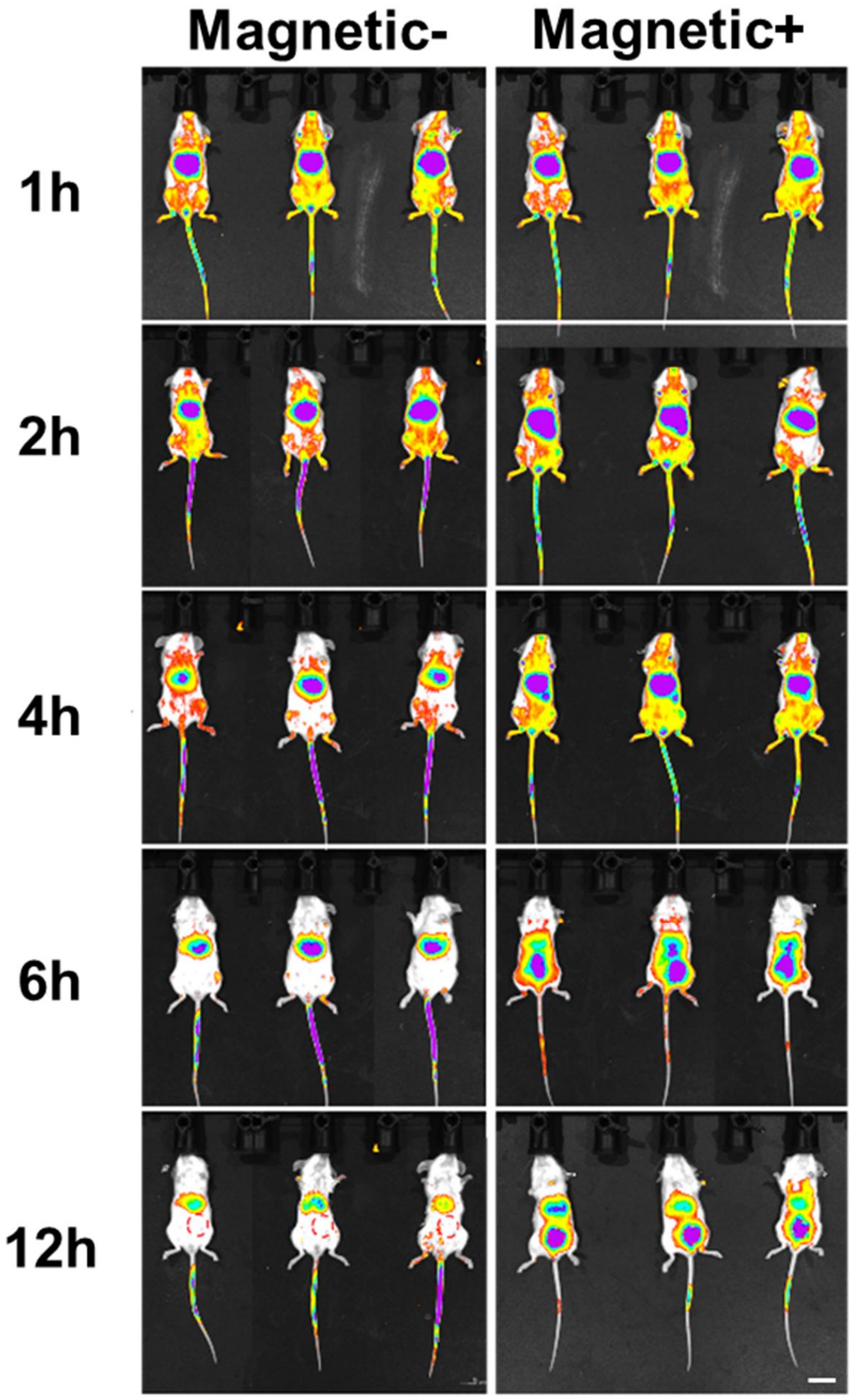

Fig. S21. Active accumulation of CD5⁺DC robots (labeled with DiR) to CRC through magnetic propulsion. *In vivo* fluorescence images illustrate the temporal distribution of DiR-labeled CD5⁺DC robots in mice bearing colorectal cancer (CRC) under magnetic propulsion (Magnetic⁺ group) versus without magnetic control (Magnetic⁻ group) at 1 h, 2 h, 4 h, 6 h, and 12 h post-injection. This analysis verifies the magnetic propulsion-mediated active accumulation of CD5⁺DC robots in CRC tumors. In the Magnetic⁺ group, CD5⁺DC robots exhibit progressive tumor enrichment over time, with sustained signal retention at 12 h. Conversely, the Magnetic⁻ group shows diffuse distribution and rapid clearance, underscoring the pivotal role of magnetic control in boosting tumor targeting efficiency. This targeted accumulation is fundamental to the CD5⁺DC robot's ability to deliver localized immunotherapy. Scale bar, 2 cm.

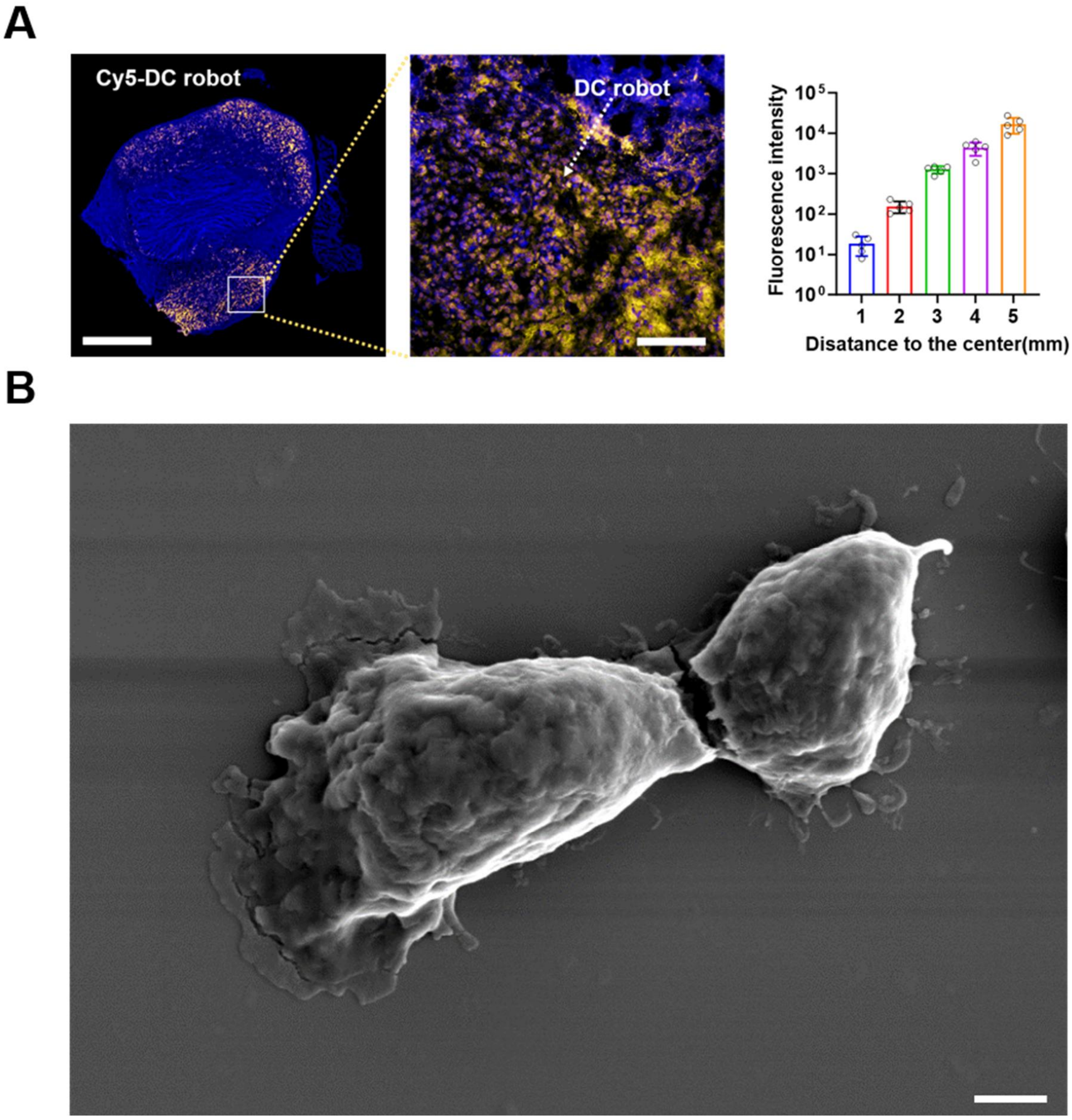

Fig. S22. CD5⁺DC robots infiltrate solid tumors and activate immune cells. (A) Confocal laser scanning microscopy images of thin sections showing $CD5^+$DC robots traversing the extracellular matrix of solid tumors. In the images, DAPI (4', 6-diamidino-2-phenylindole) is used to label cell nuclei, and Cy5 is used to label DC robots. Scale bar (left), 3 mm. Scale bar (right), 100 µm. **(B)** SEM of intercellular communication between $CD5^+$DC robots and immune cells. SEM image depicts intercellular communication between a $CD5^+$DC robot and an immune cell (e.g., $CD8^+$ T cell), characterized by prominent cellular protrusions forming direct contact (scale bar = 10 µm). This structural interaction represents a critical step for $CD5^+$DC robots to present tumor antigens and activate immune cells, aligning with the *in vivo* immunostimulatory effects observed in Fig. 4. The direct cell-cell contact ensures efficient antigen transfer and co-stimulatory signal delivery, underpinning the $CD5^+$DC robot's capacity to orchestrate antitumor immune responses. Scale bar, 2 µm.

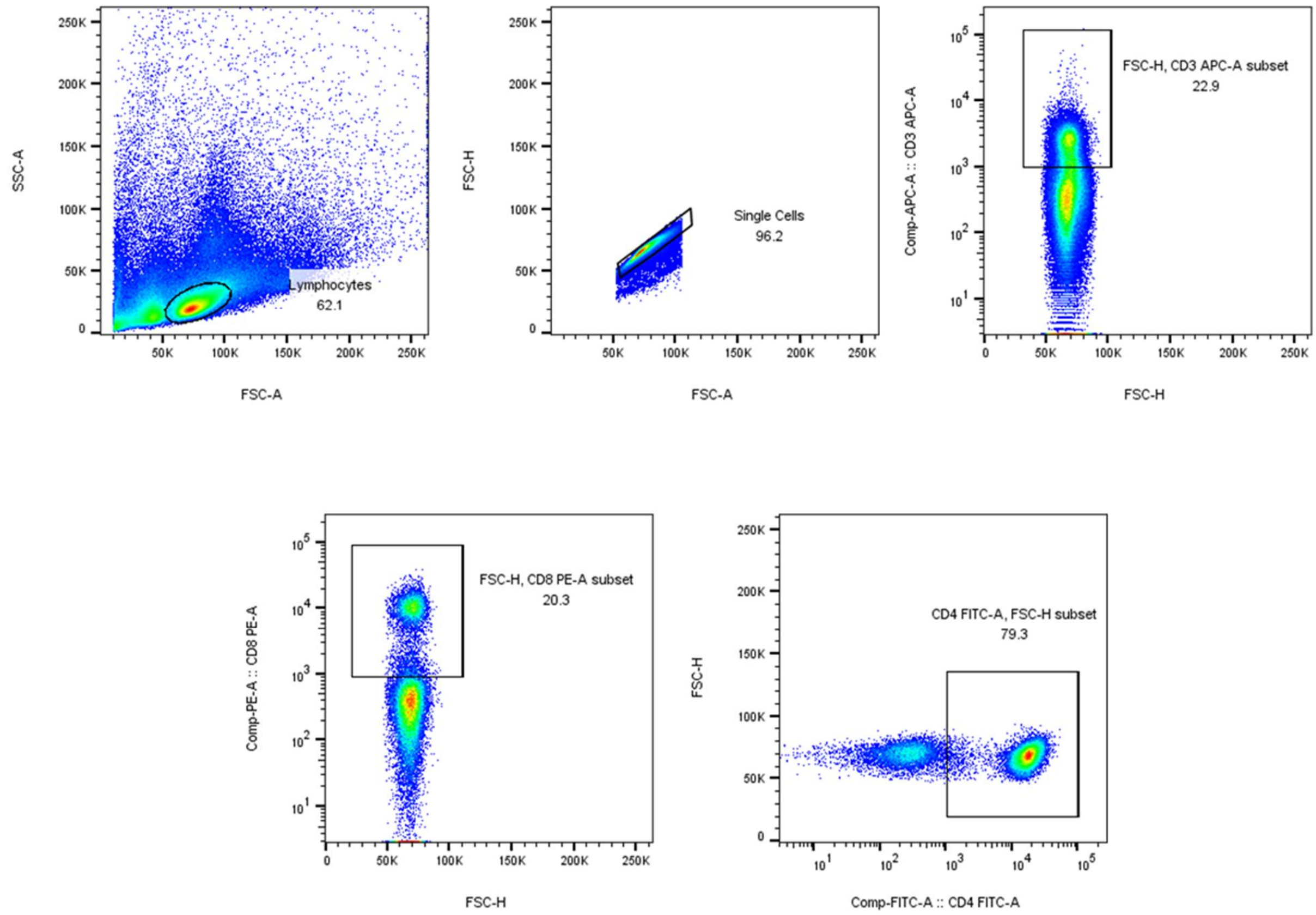

Fig. S23. Gating strategy for flow cytometric analysis of T cell subsets, sequentially showing the gating process for lymphocytes, single cells, CD3⁺ T cells, CD4⁺ T cells, and CD8⁺ T cells. This gating strategy enables precise identification and quantification of T cell subsets (total T cells, CD4⁺ helper T cells, CD8⁺ cytotoxic T cells), which is critical for assessing the immunostimulatory effects of CD5⁺DC robots. By sequentially excluding non-target populations (debris, doublets) and specifically gating on T cell lineage markers (CD3, CD4, CD8), the analysis provides reliable data on T cell composition and activation status—fundamental for interpreting CD5⁺DC robot-mediated antitumor immune responses (consistent with the flow cytometric data in Fig. 4 and Fig. S18).

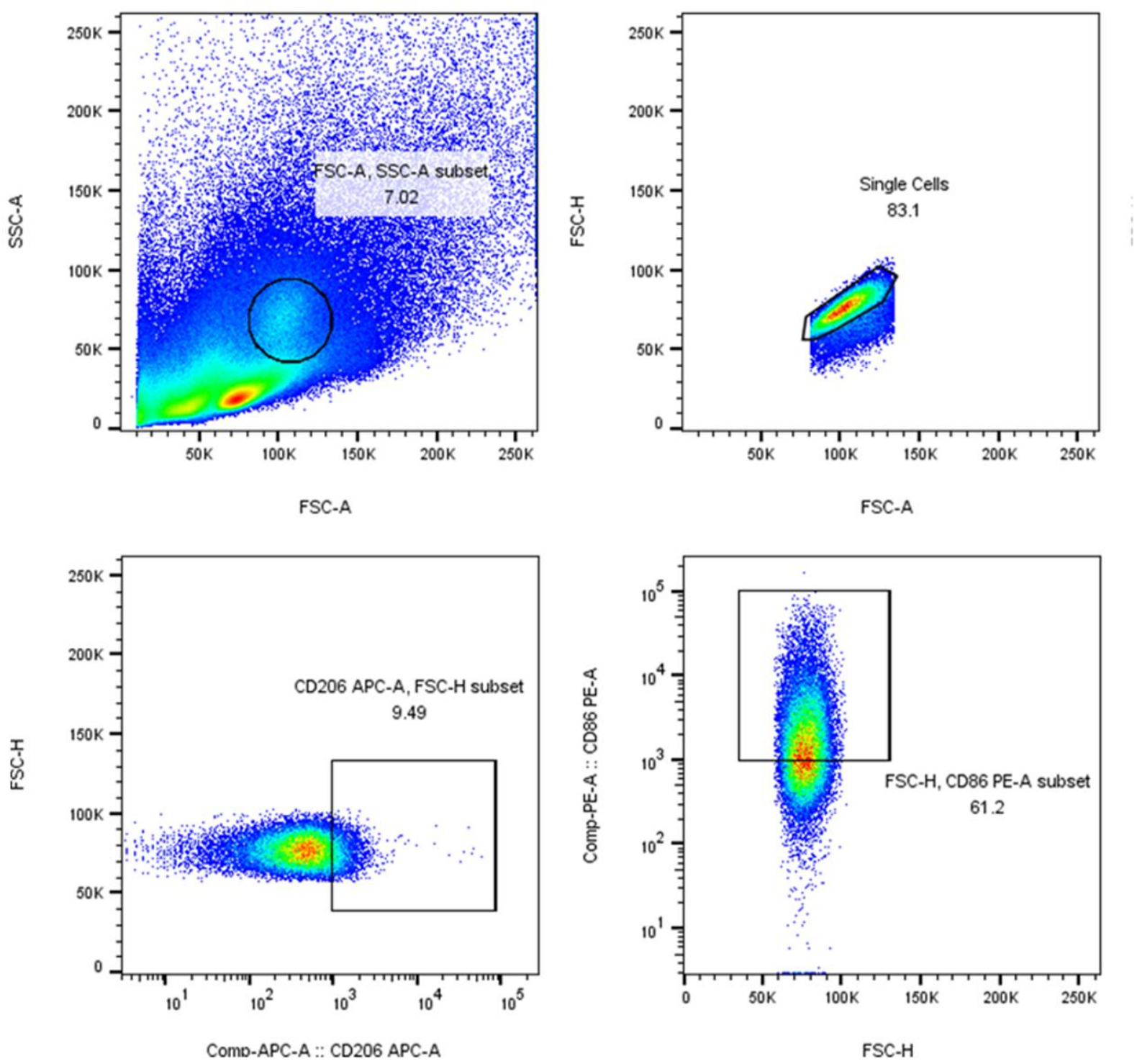

Fig. S24. Gating strategy for flow cytometric analysis of cell subsets, sequentially showing the gating process for monocytes, single cells, CD206⁺ cells, and CD86⁺ cells. This gating strategy ensures accurate identification and quantification of key immune cell subsets involved in $CD5^{+}$DC robot-mediated antitumor immunity. By sequentially excluding non-target populations (debris, doublets) and specifically gating on functional markers (CD206, CD86), the analysis provides reliable data on immune cell composition and activation status—critical for interpreting the immunomodulatory effects of $CD5^{+}$ dendritic cell robots.

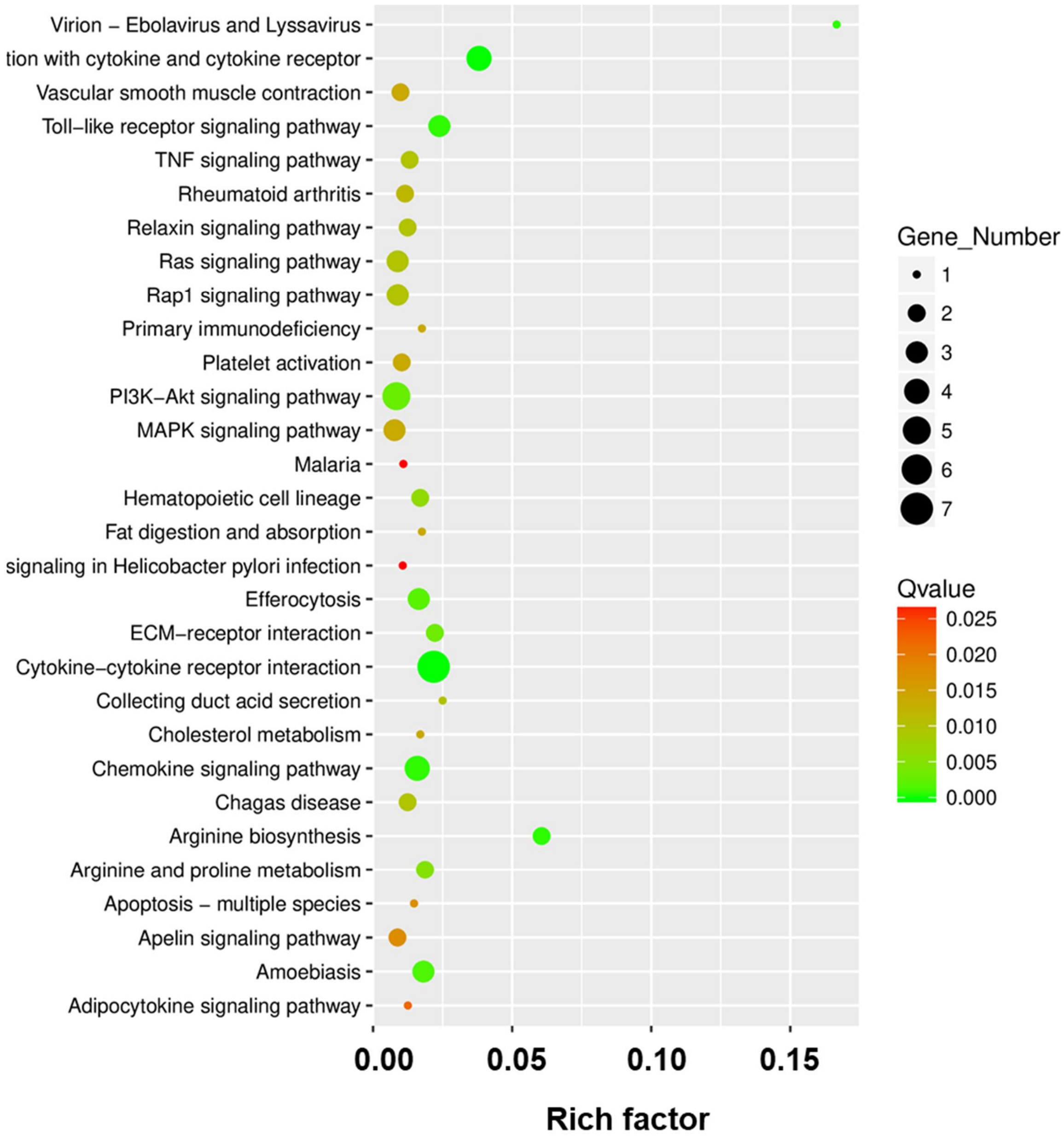

Fig. S25. KEGG pathway enrichment analysis of differentially expressed genes (DEGs) between CD5^{+}DC robot* group and PBS group. Prominent enriched pathways include cytokine-cytokine receptor interaction, Toll-like receptor signaling pathway, and PI3K-Akt signaling pathway—all critical for immune activation and inflammation. These findings align with the immunostimulatory role of CD5^{+}DC robots, as they modulate key signaling cascades to enhance antitumor immunity. The enrichment of immune-related pathways supports the mechanistic rationale for CD5^{+}DC robot-mediated tumor immunotherapy.

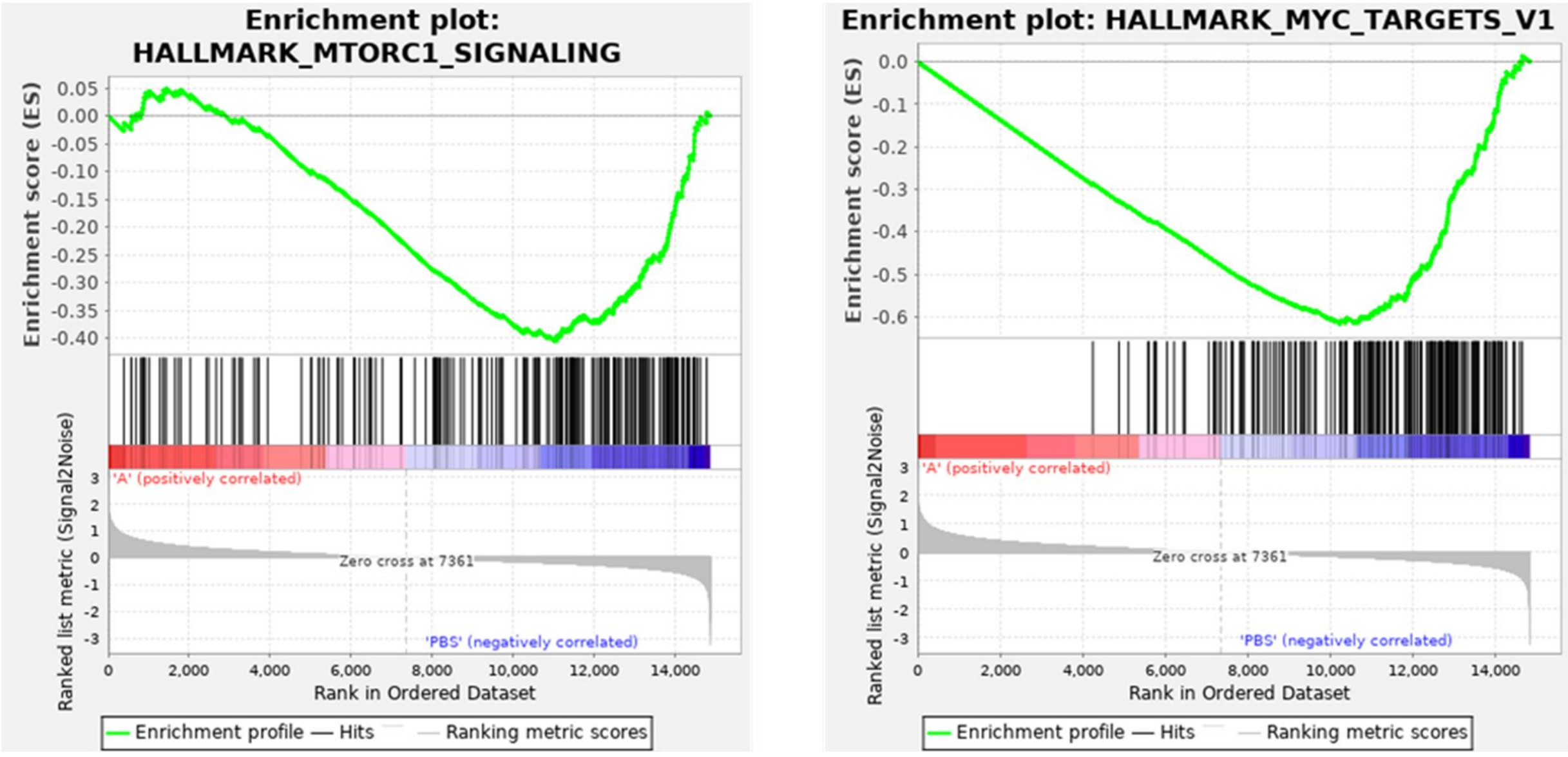

Fig. S26. GSEA analysis showed that gene sets of the MTORC signaling pathway, MYC signaling pathway were enriched in CD5⁺DC robot* group. These findings reveal that CD5⁺DC robots modulate key signaling cascades (MTORC1, MYC) to promote immune cell activation and antitumor responses. The enrichment of these pathways aligns with the functional assays (e.g., flow cytometry, *in vivo* efficacy) and supports the mechanistic basis of CD5⁺DC robot-mediated immunotherapy.

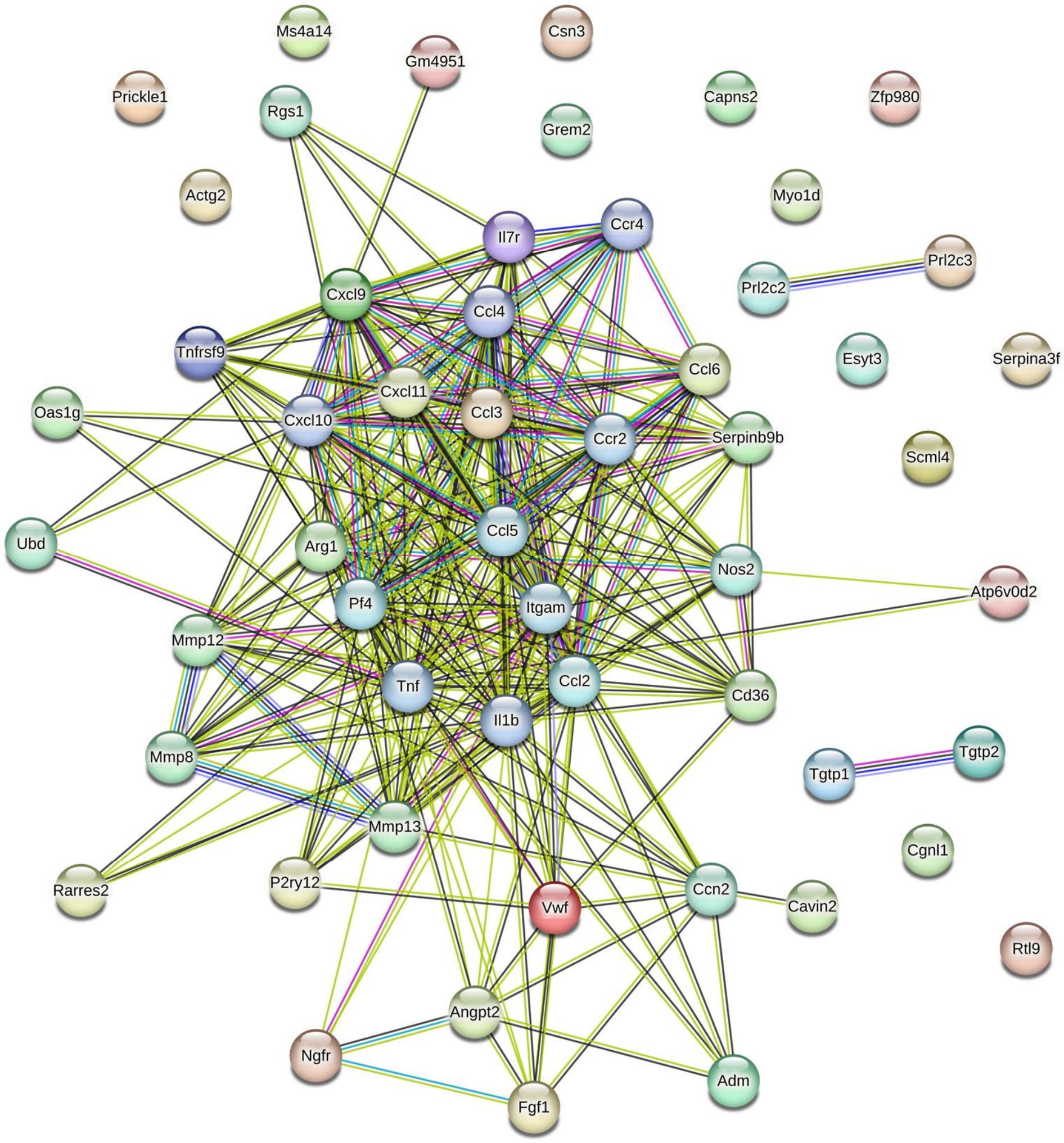

Fig. S27. Protein-protein interaction network corresponding to differentially expressed genes. This PPI network elucidates the molecular interplay underlying CD5^{+}DC robot-mediated antitumor immunity. Core proteins are involved in cytokine signaling, T cell activation, and immune cell chemotaxis, demonstrating that CD5^{+}DC robots modulate these interactions to orchestrate immune responses.

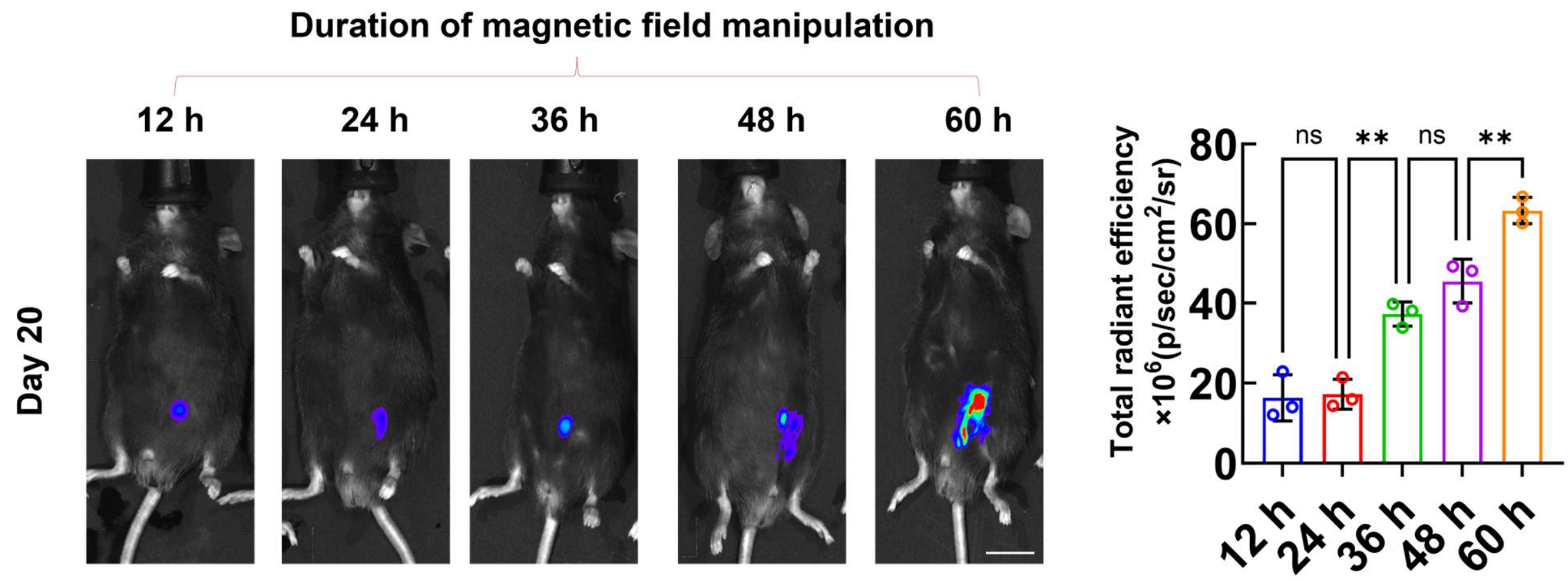

Fig. S28. Tumor fluorescence signal intensity in tumor-bearing mice after different durations of magnetic field manipulation. To investigate whether the duration of magnetic actuation affects the therapeutic efficacy, $CD5^+$DC robots were alternately exposed to an in vitro conical magnetic field (15 mT, 15 Hz) and a rotating magnetic field (20 mT, 10 Hz) for 0, 12, 24, 36, and 48 hours, then intravenously injected into tumor-bearing mice via the tail vein, followed by 12 hours of targeted magnetic actuation. On day 20, tumor fluorescence signals were detected by small animal in vivo fluorescence imaging. We found that the duration of magnetic actuation did affect the therapeutic efficacy, whereas no significant statistical difference was observed between the 0 h and 12 h in vitro magnetic actuation groups. Accordingly, the total duration of magnetic actuation only needs to be controlled within 24 h. Scale bar, 2 cm. $^*P < 0.05$; $^{**}P < 0.01$; $^{***}P < 0.001$, $^{****}P < 0.0001$.

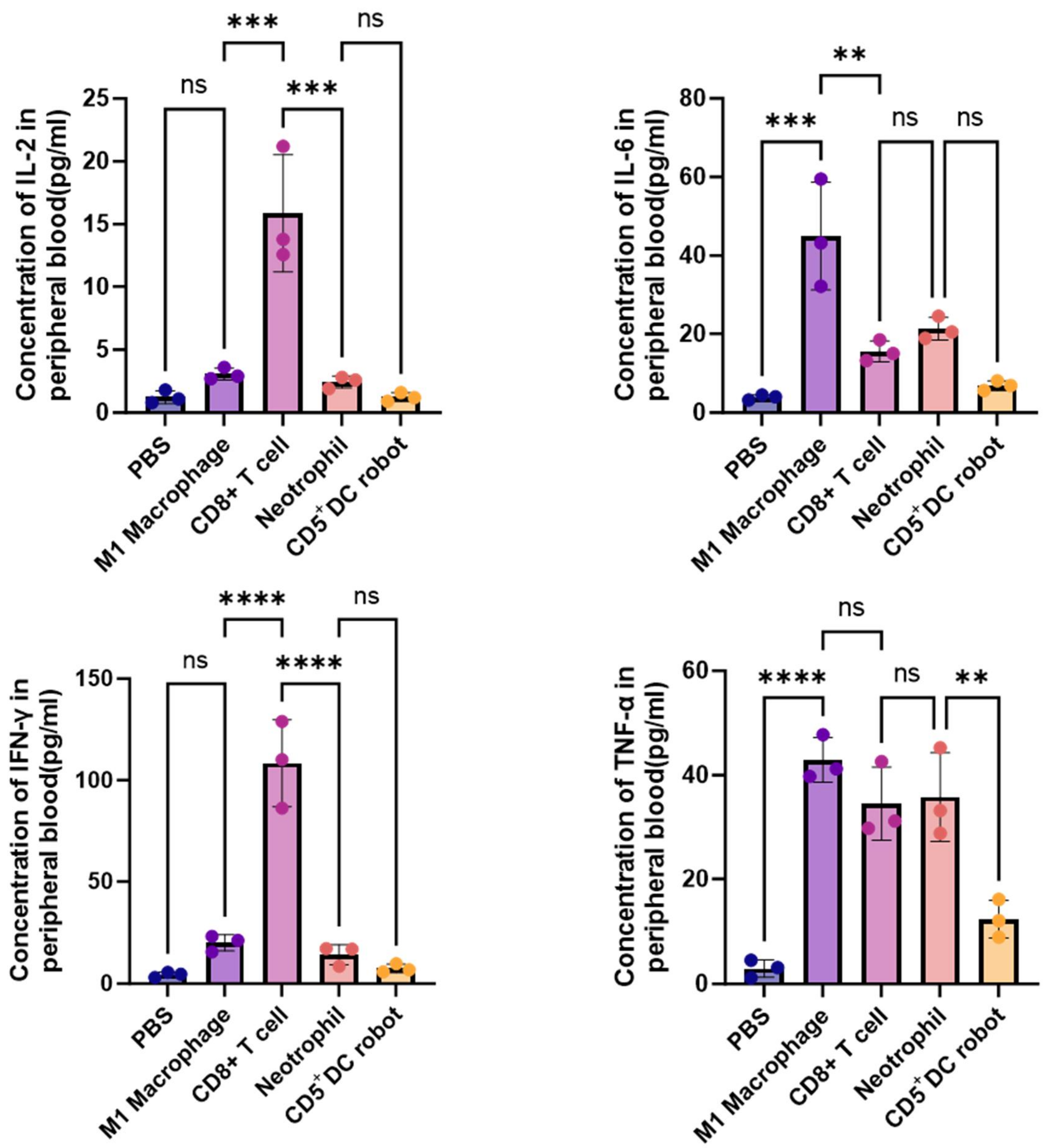

Fig. S29. Cytokine concentrations of IL-2, IL-6, IFN-γ and TNF-α, in peripheral blood of mice after adoptive transfer of different cell types. Collectively, these data demonstrate that CD5$^+$DC robots modulate proinflammatory cytokine secretion to avoid systemic inflammatory reactions—a critical advantage over conventional immune cell therapies. By restraining excessive production of IL-6, TNF-α, and other cytokines linked to systemic inflammation, CD5$^+$DC robots enable targeted tumor immunotherapy while minimizing off-target toxicities. This safety profile aligns with their design as precision immunotherapeutic agents, complementing mechanistic insights from prior molecular (Fig. S22) and pathway (Fig. S20) analyses to validate their role in regulating the tumor immune microenvironment without systemic inflammatory sequelae. *P < 0.05; **P < 0.01; ***P < 0.001, ****P < 0.0001.

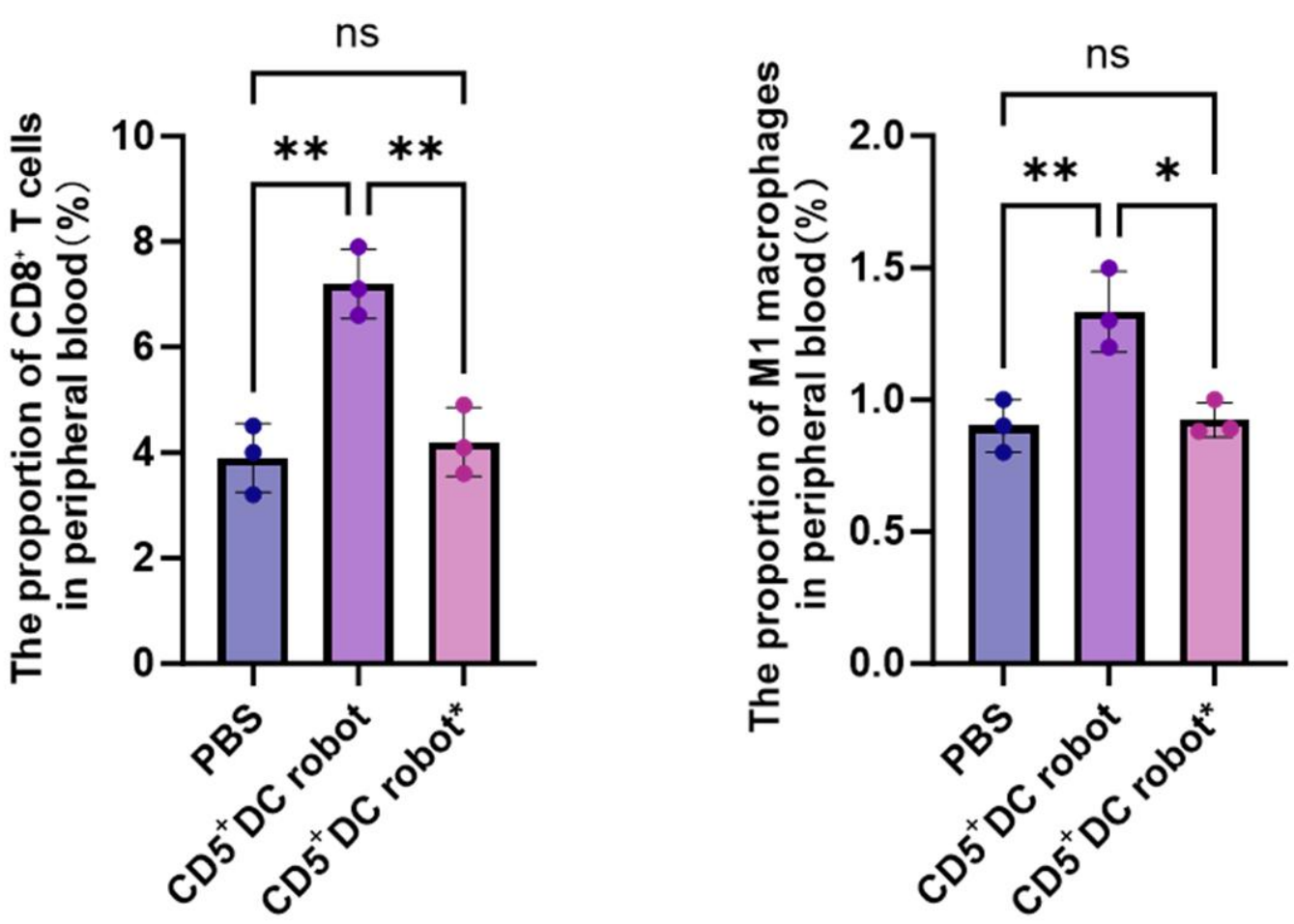

Fig. S30. The proportions of $CD8^+$ T cells and M1 macrophages in the PBS group, $CD5^+$DC robot group, and magnetically actuated $CD5^+$DC robot ($CD5^+$DC robot*) group. The non-magnetically actuated $CD5^+$DC robots indeed increased the proportions of $CD8^+$ T cells and M1 macrophages in peripheral blood, whereas the magnetically actuated $CD5^+$DC robot group showed no significant difference compared with the PBS group, indicating favorable biosafety. *P < 0.05; **P < 0.01; ***P < 0.001, ****P < 0.0001.

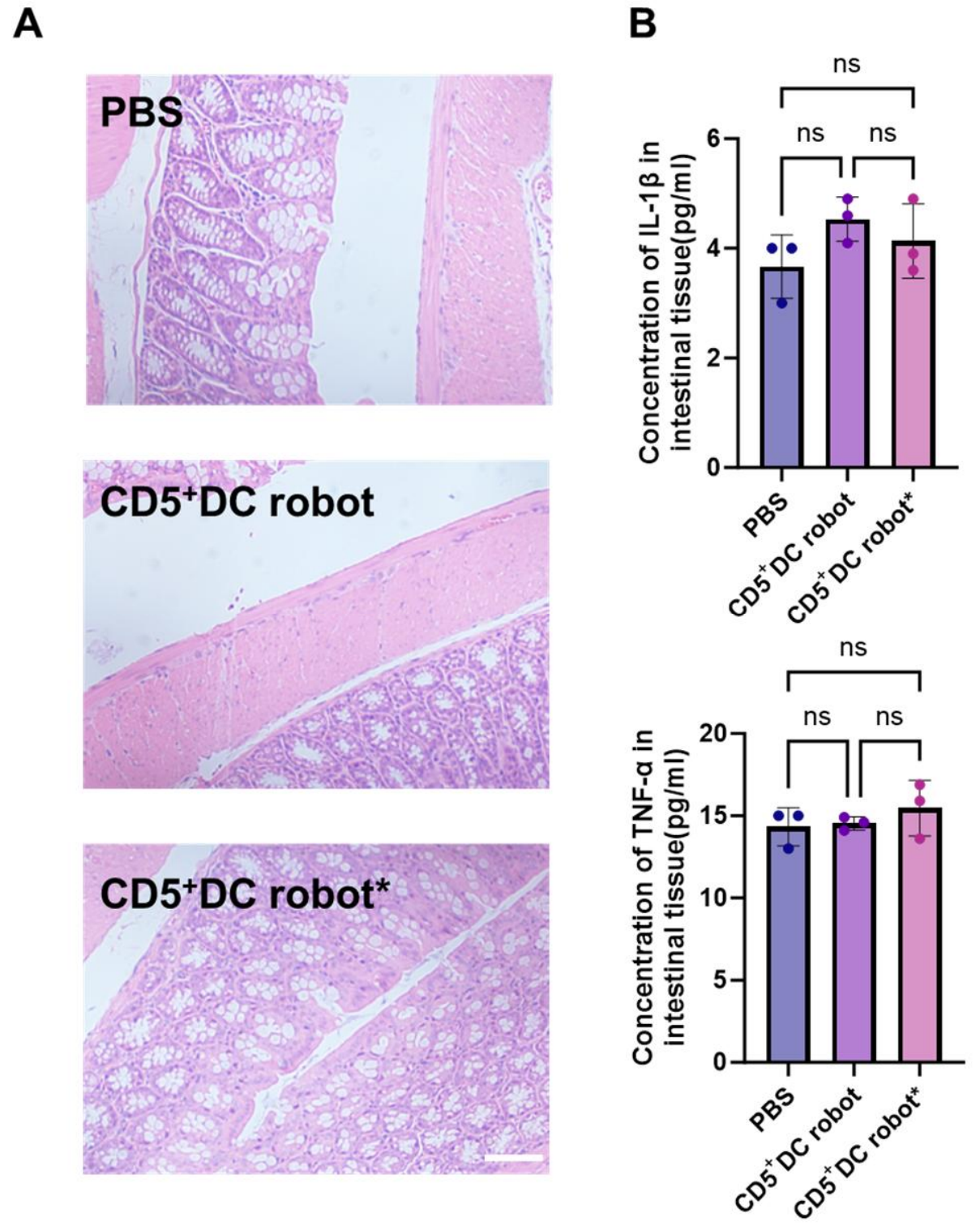

Fig. S31. Inflammatory levels in intestinal tissues after treatment with PBS, $CD5^+$DC robots, and magnetically actuated $CD5^+$DC robots ($CD5^+$DC robot*). Scale bar, 100 μm. **(A)** HE staining images of intestinal tissues for the PBS, $CD5^+$DC robot and $CD5^+$DC robot* groups. **(B)** ELISA results of IL-1β and TNF-α in intestinal tissues from the PBS, $CD5^+$DC robot and $CD5^+$DC robot* groups. *P < 0.05; **P < 0.01; ***P < 0.001, ****P < 0.0001.

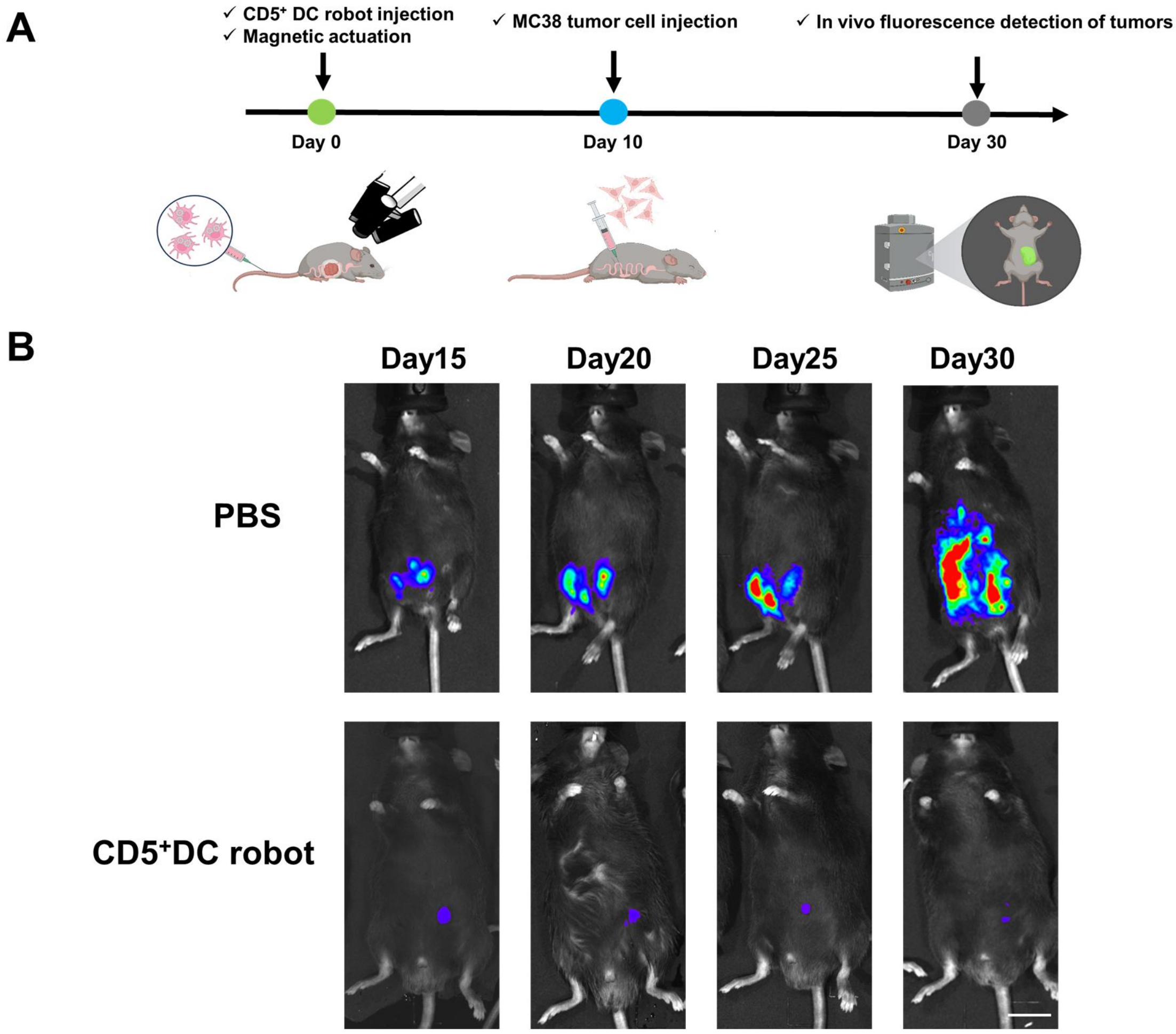

Fig. S32. CD5⁺DC robots exert preventive effects against tumorigenesis *in vivo*. (A) Schematic illustration of the experimental protocol for tumor prevention by CD5⁺DC robots. **(B)** *In vivo* fluorescence imaging results. Scale bar, 2 cm. To verify the promising potential of CD5⁺DC robots in tumor prevention, we conducted supplementary experiments: mice were divided into two groups, one injected with PBS as control and the other administered CD5⁺DC robots with magnetic targeting to the cecum. Ten days later, MC38 tumor cells were inoculated into the mouse cecum, and in vivo tumor fluorescence was monitored every five days. We found that the tumor fluorescence signal in the CD5⁺DC robot group was significantly lower than that in the PBS group, confirming its effective tumor-preventive effect. These findings highlight their application value in early immune surveillance and preventive antitumor vaccination. Schematics created in BioRender. chuanhua, L. (2026) https://BioRender.com/326iwkk.

SUPPLEMENTARY MOVIES

Movies S1 to S14

Movie S1. The CD5$^+$DC robot moves along a predetermined multi-right-angle turning trajectory under a rotating magnetic field.

Movie S2. The CD5$^+$DC robot travels along a preset star-shaped trajectory under a rotating magnetic field.

Movie S3. The CD5$^+$DC robots form a single-chain assembly under a rotating magnetic field.

Movie S4. The CD5$^+$DC robots form a single ribbon-like assembly under a conical magnetic field.

Movie S5. The CD5$^+$DC robots form a single vortex assembly under a rotating magnetic field.

Movie S6. A large number of CD5$^+$DC robots form a multi-chain assembly under a rotating magnetic field.

Movie S7. A large number of CD5$^+$DC robots form multiple ribbon-like assemblies under a conical magnetic field.

Movie S8. The CD5$^+$DC undergo chemotactic movement toward tumor tissue in response to chemokines from it.

Movie S9. The CD5$^+$DC robot undergo chemotactic movement toward tumor tissue in response to chemokines from it.

Movie S10. Clearing treatment enables real-time imaging of the abdominal intestinal blood vessels *in vivo*.

Movie S11. CD5$^+$DC robots exhibit ribbon-like swarming motion *in vivo*.

Movie S12. Chain-like CD5$^+$DC robot swarms adhere to the vascular wall to achieve retrograde movement against blood flow *in vivo*.

Movie S13. Chain-like CD5$^+$DC robot swarm adhere to the vascular wall to enable stable vascular retention against blood flow *in vivo.*

Movie S14. Chain-like CD5$^+$DC robot swarm can traverse narrow microvessels (approximately 20 μm in width) without causing blockage, enabling them to reach distant tumor tissues *via* the systemic circulatory system.
